# Supplementary material for: Measuring the molecular origins of stiffness in organic semiconductors
Source: Nat Commun. 2026 Jan 13;17:1621. doi: 10.1038/s41467-026-68328-0 (PMC12905142; doi:10.1038/s41467-026-68328-0)
Supplement: Supplementary file 1 — Supplementary Information [file 41467_2026_68328_MOESM1_ESM.pdf]

## Supplementary Information

# Measuring the molecular origins of stiffness in organic semiconductors

Ki-Hwan Hwang,[1,2] † Dorothée Brandt,[3] Silvia Cristofaro,[3] Cameron J. Nickerson,[4] Federico Modesti,[5] Mindaugas Gicevičius,[1] Mateo T. R. Cervantes,[1] Martina Volpi,[6] Leszek J. Spalek,[1,7] Luca Muccioli,[8] Per M. Claesson,[2] Ljiljana Fruk,[7] Yves Geerts,[6,9] Guillaume Schweicher,[6] Yoann Olivier,[3] Erin R. Johnson,[4,10,11] Deepak Venkateshvaran[1,7,12]†

[1] *Cavendish Laboratory, University of Cambridge, JJ Thomson Avenue, Cambridge CB3 0HE, United Kingdom*

[2] *KTH Royal Institute of Technology, School of Engineering Sciences in Chemistry, Biotechnology and Health, Department of Chemistry, Division of Surface and Corrosion Science, Teknikringen 30, Stockholm, SE-100 44 Sweden*

[3] *Laboratory for Computational Modeling of Functional Materials, Namur Institute of Structured Matter, University of Namur, Rue de Bruxelles 61, 5000 Namur, Belgium*

[4] *Department of Physics and Atmospheric Science, Dalhousie University, 6310 Coburg Rd, Halifax, NS, B3H 4R2, Canada*

[5] *BASF SE, Carl-Bosch-Strasse 38, 67056 Ludwigshafen am Rhein, Germany*

[6] *Laboratoire de Chimie des Polymères, Faculté des Sciences, Université Libre de Bruxelles (ULB), CP 206/01, Boulevard du Triomphe, 1050 Brussels, Belgium*

[7] *Department of Chemical Engineering and Biotechnology, University of Cambridge, Philippa Fawcett Drive, Cambridge CB3 0AS, United Kingdom*

[8] *Department of Industrial Chemistry "Toso Montanari", University of Bologna, via Gobetti 85, 40129 Bologna, Italy*

[9] *International Solvay Institutes of Physics and Chemistry, Université Libre de Bruxelles (ULB), CP 231, Boulevard du Triomphe, 1050 Brussels, Belgium*

[10] *Yusuf Hamied Department of Chemistry, University of Cambridge, Lensfield Road, Cambridge CB2 1EW, United Kingdom*

[11] *Department of Chemistry, Dalhousie University, 6274 Coburg Road, Halifax, Nova Scotia B3H 4R2, Canada*

[12] *Department of Science Innovation and Technology, UK Government, 100 Parliament Street, London SW1A 2BQ, United Kingdom*

†*email:* kh826@cam.ac.uk, dv246@cam.ac.uk

## Table of Contents


**SI Section 1**
Accompanying mechanical properties of DNTT and its derivatives extracted using the force-distance curves **– page 3**

**SI Section 2**
Photothermal calibration of AFM cantilevers for mechanical measurements **– page 4**

**SI Section 3**
Lack of correlation between topography and stiffness in measurements **– page 6**

**SI Section 4**
Function of molecular densities of DNTT and its derivatives on side chains **– page 10**

**SI Section 5**
Mechanical properties as a function of setpoint, discussion on nuances such as tip shear and plastic deformation in nanomechanical measurements, and influence of contamination of cantilever tip in nanomechanical property measurements **– page 12**

**SI Section 6**
Young's Modulus Calculations (DFT) **– page 57**

**SI Section 7**
Mechanical properties determination employing Molecular Dynamics (MD) simulations **– page 61**

**SI Section 8**
Transfer (linear regime) and output characteristics of field-effect transistors based on DNTT, C8-DNTT-C8, RR-C8*-DNTT-C8* and SS-C8*-DNTT-C8* **– page 79**

**Bibliography** **– page 81**

**SI Section 1**
**Accompanying mechanical properties of DNTT and its derivatives extracted using the force-distance curves**

In the main manuscript, the focus was on the stiffness extracted from the measured force distance curves. In Supplementary Figure 1 below, the accompanying mechanical parameters extracted from the force distance curves are shown. These are the Young's modulus (extracted using the Hertz model), the adhesion force, and the nanoindentation. These measurements are shown to demonstrate consistency and trustworthiness of the stiffness measurements. The adhesion force is very similar for all the samples, showing that the trends in the measurements of stiffness are not skewed by samples being adhesive. Also seen in Supplementary Figure 1 is that the indentation of the DNTT film and its derivatives are in the few nanometres range. A comparable indentation on all samples was maintained. Thus, the trends in the stiffness we report are removed of artefacts arising from either stickiness of the samples or from varying indentation depths.

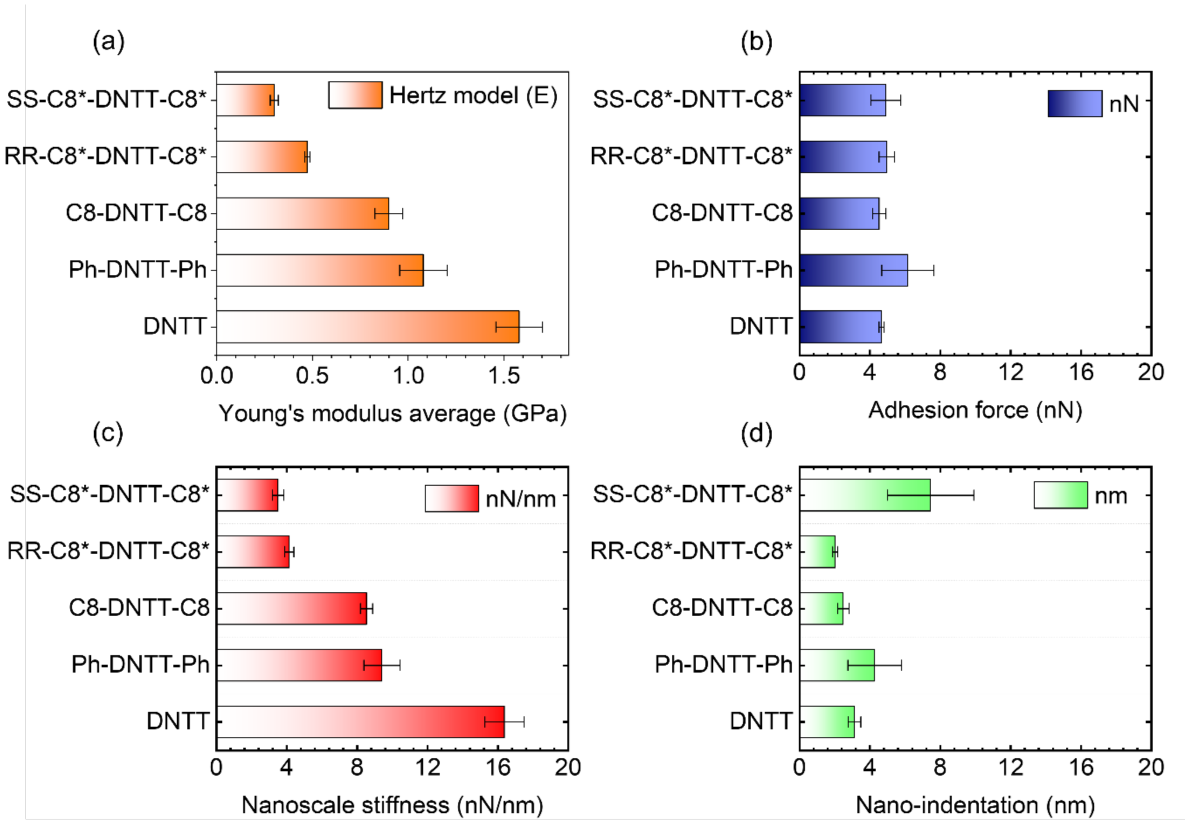


**Supplementary Figure 1** (a) Nanoscale stiffness of DNTT and its derivatives as reported in the main manuscript, (b) Young's Modulus of the films calculated using the Hertz model, (c) Adhesion forces extracted from the force distance curves, (d) Indentation of the surfaces that accompany the estimation of the stiffness.

## SI Section 2
## Photothermal calibration of AFM cantilevers for mechanical measurements

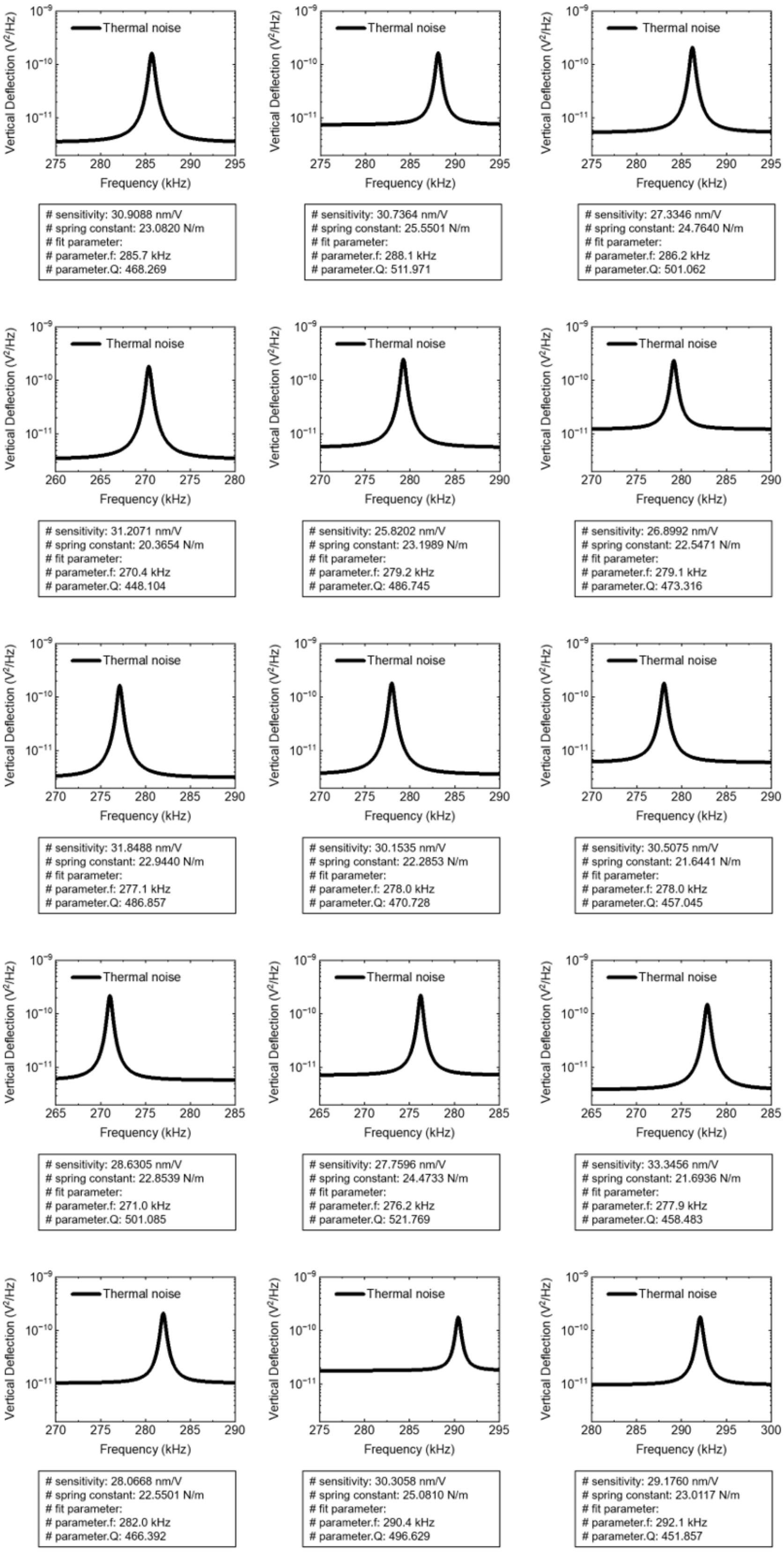

**Supplementary Figure 2** Spring constants of cantilevers using a photothermal excitation calibration (sometimes called a thermal tuning method or a thermal noise method) prior to the measurement of nanomechanics. The figure shows the calibration of all the cantilevers that were used prior to the scans on each sample.

As nanomechanics of samples are measured using a non-resonant cantilever technique, i.e., the AFM cantilever is not under piezo-actuated resonant oscillation during measurement of the force distance curves, calibration of the AFM cantilevers prior to measurements are done using a photothermal excitation or a thermal noise/tune measurement. Supplementary Figure 2 shows the properties extracted from the thermal tuning of each cantilever prior to the nanomechanical measurements. The position of each of the cantilever calibration curves in Supplementary Figure 2 corresponds to the sample measurement shown in Supplementary Figure 3. The top row corresponds to DNTT measurements on the three different areal scans, L, M and S. The second row is that of Ph-DNTT-Ph. The third row is that for C8-DNTT-C8 and the final two rows cover RR-C8*-DNTT-C8* and SS-C8*-DNTT-C8*.

A new AFM cantilever was used for each sample measurement scan. For this reason, each cantilever was calibrated separately prior to each scan. The agreement in the spring constants, sensitivities, first eigen modes, and Q-factors, of all the cantilevers across all scans removes any cantilever dependent artefacts in the measurements of nanomechanics.

## SI Section 3
## Lack of correlation between topography and stiffness in measurements

As explained in the main manuscript, when carrying out nanomechanical measurements of samples, the same force distance curve is used in contact point imaging (CPI) to extract the topography, and the other mechanical properties. A demonstration of the lack of correlation between the measured topography and the measured stiffness of the films is necessary to ensure trustworthiness of the mechanical measurements. Supplementary Figure 3 shows this lack of correlation on all measurements carried out in the main manuscript.

(a)

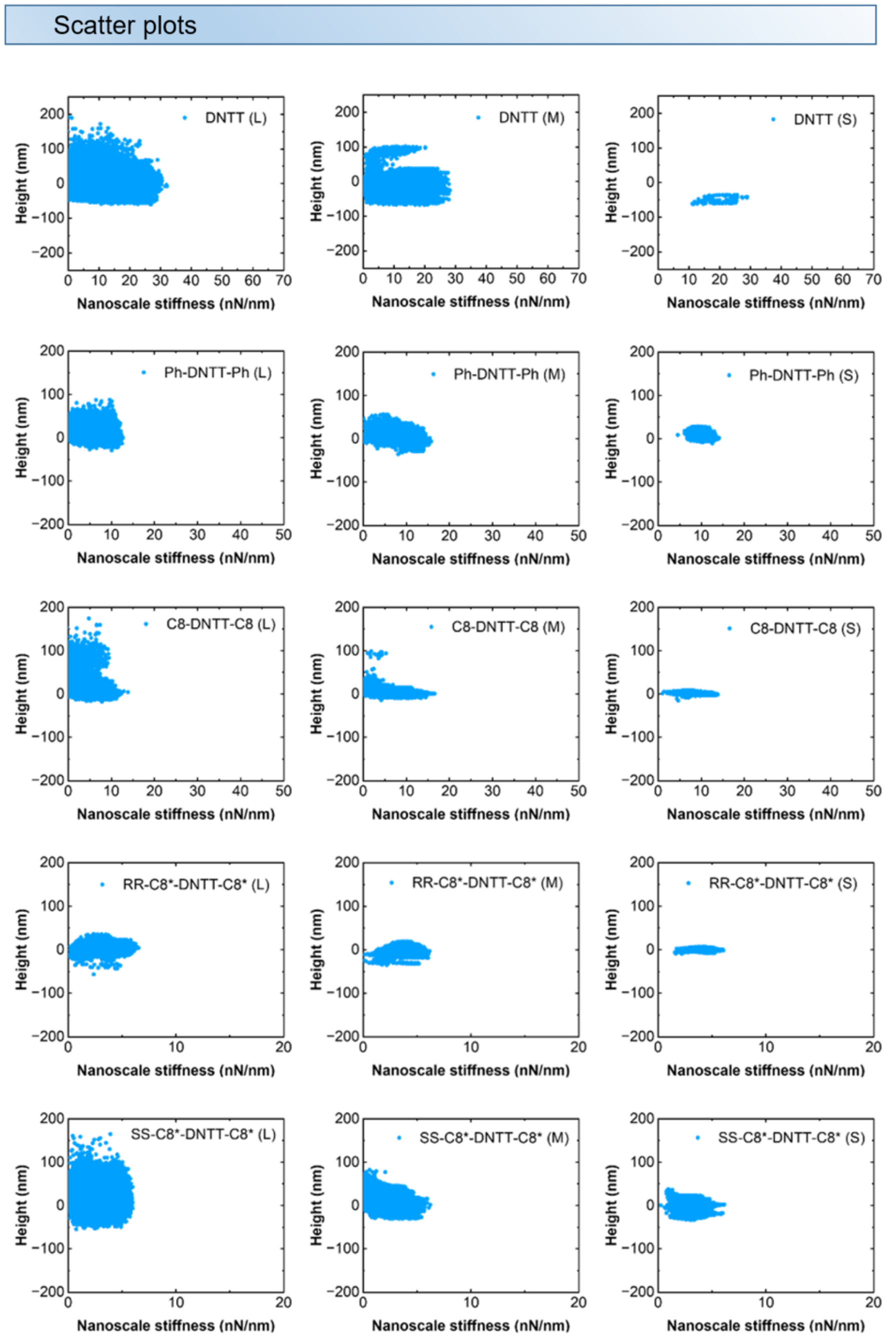

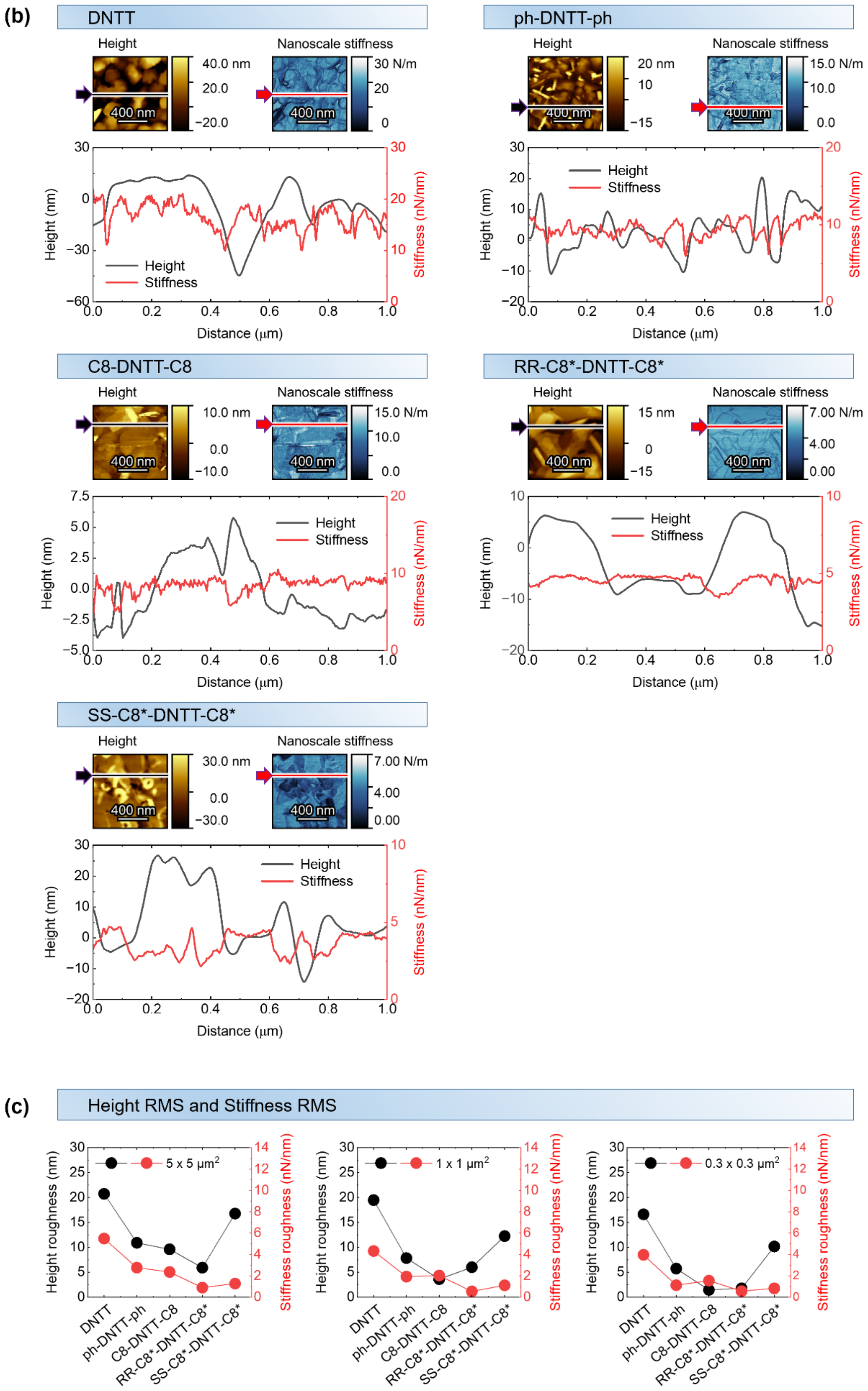


**Supplementary Figure 3** (a) Scatter plots of measured height topography and measured stiffness of the DNTT film and its derivatives. L, M, and S are the measurements on 5 x 5

μm², 1 x 1 μm² and 0.3 x 0.3 μm². (b) Line-scan profiles of the DNTT film and its derivatives. (c) root-mean-square (RMS) roughness graphs of height and stiffness for the DNTT film and its derivatives.

As seen from the scatter plots in Supplementary Figure 3 (a), the topography and the stiffness of the samples remain uncorrelated. If, for example, there are deep crevices in the films, and the AFM tip was unable to go all the way through to the bottom of the crevice to indent it and measure its stiffness, a correlation between topography and stiffness may have been seen. The fact that no correlation is observed in any of our measurements demonstrates again that the measurement of nanomechanics is free from spurious measurement artefacts. This is further confirmed in Supplementary Figure 3 (b) and (c). The line profile in (b) shows that it is difficult to find a correlation between the topography and stiffness. Additionally, Supplementary Figure 3 (c), which compares roughness using RMS—one of the statistical methods for comparing the characteristics of the films—also confirms the lack of correlation. The trends in the RMS values are not the same between the two datasets, and the sequential order of the RMS values for height and stiffness is different. Ultimately, this indicates that there is no direct relationship between height and stiffness.

**Additional analysis based on Pearson correlation coefficients**

In addition to the analysis above, we quantified the correlation between Height and Stiffness maps by computing the sample correlation coefficient, also known as the *Pearson correlation coefficient*, defined as follows:

Given a series of $n$ measurements of the pair $(x_i, y_i) \in X \times Y$ indexed by $i = 1, \ldots, n$, the sample correlation coefficient is defined as

$$r_{xy} := \frac{\sum_{i=1}^{n}(x_i - \bar{x})(y_i - \bar{y})}{(n-1)s_x s_y}$$

where $\bar{x}$ and $\bar{y}$ are the sample means of $X$ and $Y$ , and $s_x$ and $s_y$ are their corrected sample standard deviations.

The value of a correlation coefficient ranges between $-1$ and $+1$. A correlation coefficient of $+1$ indicates a perfect direct (increasing) linear relationship, while a value of $-1$ indicates a perfect inverse (decreasing) linear relationship (anti-correlation). For any other value within the open interval $(-1, 1)$, the coefficient reflects the degree of linear dependence between the variables. As the coefficient approaches zero, the relationship between the variables weakens, indicating less correlation. The closer the coefficient is to $-1$ or $1$, the stronger the correlation. If $X$ and $Y$ are the results of measurements that include measurement error, the realistic limits of the correlation coefficient may fall within a narrower range than $-1$ to $+1$.

In the context of AFM maps, both Height and Stiffness are measured simultaneously at each point. Therefore, we can associate each of these maps with the $X$ and $Y$ data sets, respectively, as defined above. Since each measurement is performed independently, the degree of correlation between them can be quantified using the aforementioned formula. The results of this analysis are presented in Supplementary Figure 4. Notably,

the absolute values of the correlation coefficients are consistently below 0.45, and in most cases below 0.2. This suggests that there is no direct relationship between Height and Stiffness.

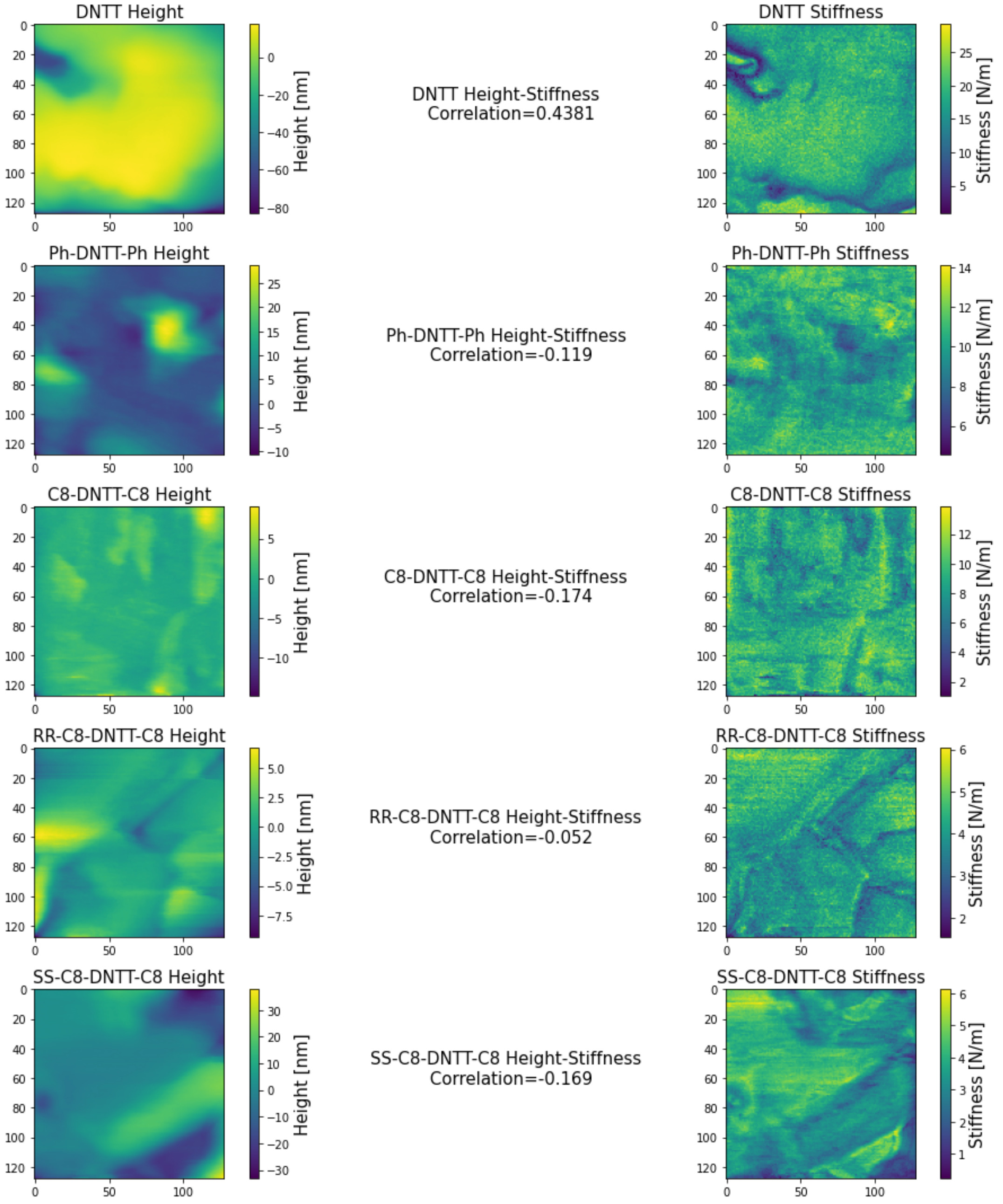


**Supplementary Figure 4** AFM height and stiffness maps of the DNTT film and its derivatives. The height-stiffness correlation is indicated between each pair of maps.

## SI Section 4
## Function of molecular densities of DNTT and its derivatives on side chains

Supplementary Figure 5 shows images of the unit cells of DNTT and its derivatives, and their volumetric expansion upon side chain functionalisation. As the sidechains go from being non-existent in the case of DNTT, through to Ph-DNTT-Ph, and to C8-DNTT-C8, there is an expansion in the unit cell. In going from C8-DNTT-C8 to the chiral versions of SS-C8*-DNTT-C8* or RR-C8*-DNTT-C8*, an area expansion in the ab-plane causes further volumetric expansion. The molecular density reported in the main paper is computed as: $\text{density (molecules per nm}^3) = \frac{\text{number of the molecules}}{\text{unit cell volume}}$.

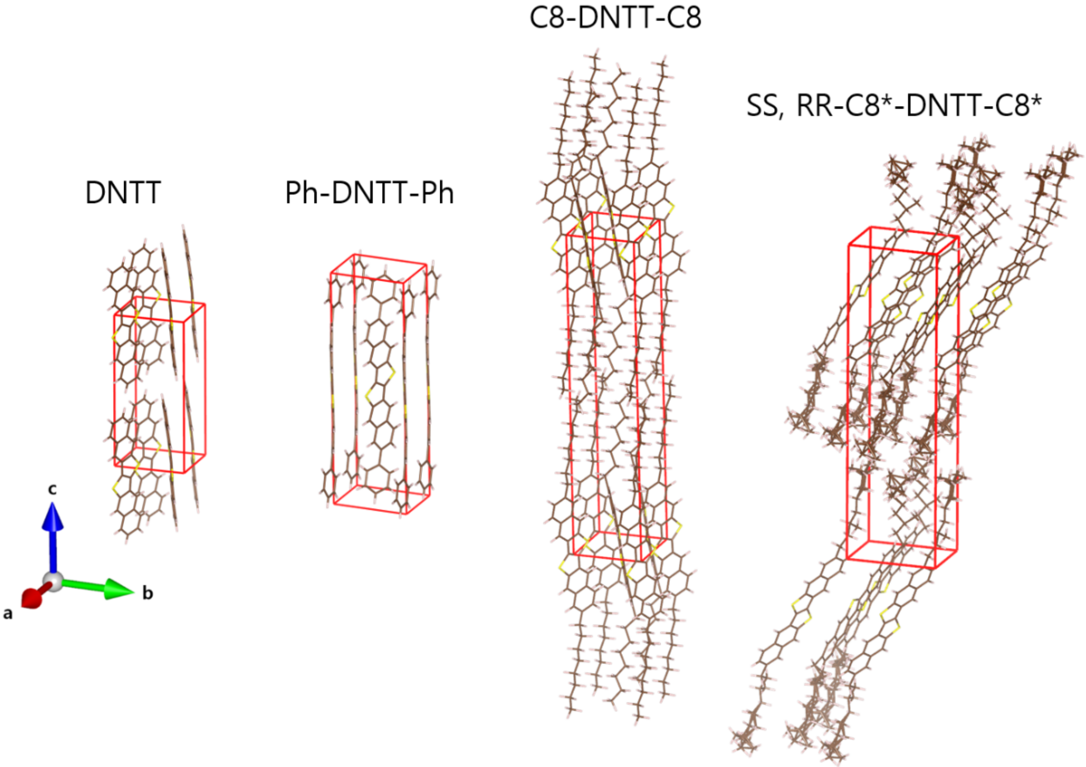


**Supplementary Figure 5** Configuration of unit cells in lattices of DNTT and its derivatives.

| | a (Å) | b (Å) | c (Å) | α (degree) | β (degree) | γ (degree) | Area (ab-plane) | Volume (nm$^3$) |
|---|---|---|---|---|---|---|---|---|
| **DNTT** | 6.19 | 7.66 | 16.21 | 90.00 | 92.49 | 90.00 | 0.47 | 0.77 |
| **Ph-DNTT-Ph** | 6.18 | 7.61 | 24.32 | 90.00 | 85.70 | 90.00 | 0.47 | 1.14 |
| **C8-DNTT-C8** | 5.99 | 7.86 | 34.07 | 90.00 | 99.86 | 90.00 | 0.47 | 1.58 |
| **SS, RR-C8*-DNTT-C8*** | 6.04 | 9.25 | 32.90 | 90.51 | 91.34 | 93.78 | 0.56 | 1.83 |

**Supplementary Table 1** Properties of the unit cells, area in the ab-plane, and volume of the unit cells of DNTT and its derivatives. The area in the ab-plane remains similar for DNTT, Ph-DNTT-Ph, and C8-DNTT-C8. In the case of SS-C8*-DNTT-C8* and RR-C8*-DNTT-C8*, the overall volume expansion beyond what is seen for DNTT comes from expansion in the ab-plane.

**SI Section 5**

**Mechanical properties as a function of setpoint, discussion on nuances such as tip shear, plastic deformation in nanomechanical measurements, and the influence of cantilever tip contamination in nanomechanical property measurements**

In doing atomic force microscopy at high resolution over nanoscale areas, methods such as Quantitative Imaging (QI) mode (from JPK, Bruker), Pinpoint Nanomechanical mode (from Park systems), Peak Force (from Bruker) and so on, allow an integrated analysis of measured topology as well as a statistical approach to nanomechanical properties. These techniques are widely used and are considered very potent. [1], [2], [3], [4], [5], [6]

When measuring the nanomechanical properties of soft organic materials using AFM, only limited information on cantilever bending is obtained via the laser beam alignment system. As such, it is essential to understand how force–distance curves can vary under different experimental conditions and to recognize subtle factors that may influence measurement accuracy. One important consideration is the choice between using the approach or retract force curve, with the approach curve generally being more appropriate for soft organic materials.

Furthermore, we investigate potential sources of error when analyzing materials with strong in-plane orientation, such as DNTT or other liquid-crystalline materials, including measurement artifacts caused by lateral slippage during indentation, and inaccuracies resulting from tip contamination by small molecules. To evaluate these effects, we performed measurements at various setpoints and carried out controlled tip contamination experiments, allowing us to assess the impact of these factors on the accuracy and reliability of nanomechanical measurements.

**I. The influence of setpoint on the measured values of elastic properties**

In Section I, we briefly discussed the application of the Hertz and DMT models to both approach and retract curves. The focus was placed on exploring the measurement anomalies that may arise in well-aligned liquid crystalline materials such as DNTT, along with a discussion of their possible origins.

If we were to explain the method by which we obtain mechanical properties through AFM very simply, it is a process of obtaining elastic information about an unknown spring using a well-characterised spring, as depicted in Supplementary Figure 6.

Methodologically, this can be thought of as being like using a nanoindenter with a pyramidal diamond tip. [7], [8]. However, in this conventional method using indentation for hard materials, a plastic deformation is typically present. Since it is challenging to separate plastic deformation from elastic deformation in the approach curve, the elastic properties are evaluated using the retracting curve with the Sneddon method. [8]

In contrast, measurements based on AFM commonly deal with organic materials that exhibit sufficient elastic deformation at relatively very low forces. Therefore, using the approach curve is beneficial. Additionally, depending on the material being measured, it is known that, if a clear analysis of indentation is possible, analysis with Angstrom-scale

indentation is sufficient. [9] Such studies indicate the utility of the approach curve when measuring mechanical properties based on AFM. However, there is still ongoing discussion about which curve to use when there is a difference in the contact point position between the approach curve and the retract curve even in cases where plastic deformation cannot be confirmed. [10]

In short, when there is no difference in the slopes of the forward and retract curves, there is no argument. However, if there is a noticeable difference in the slopes of the curves depending on the chosen curve, a significant debate can arise because this may lead to substantial variations in the measured values of elastic properties.

To gain a clear understanding and derive accurate measurement results, we conducted experiments on the elastic modulus using various setpoints for the two different curves (approach and retract) as a preliminary assessment. In addition to the experiments on elastic modulus, we also investigated the impact of residual molecules on the tip edge.

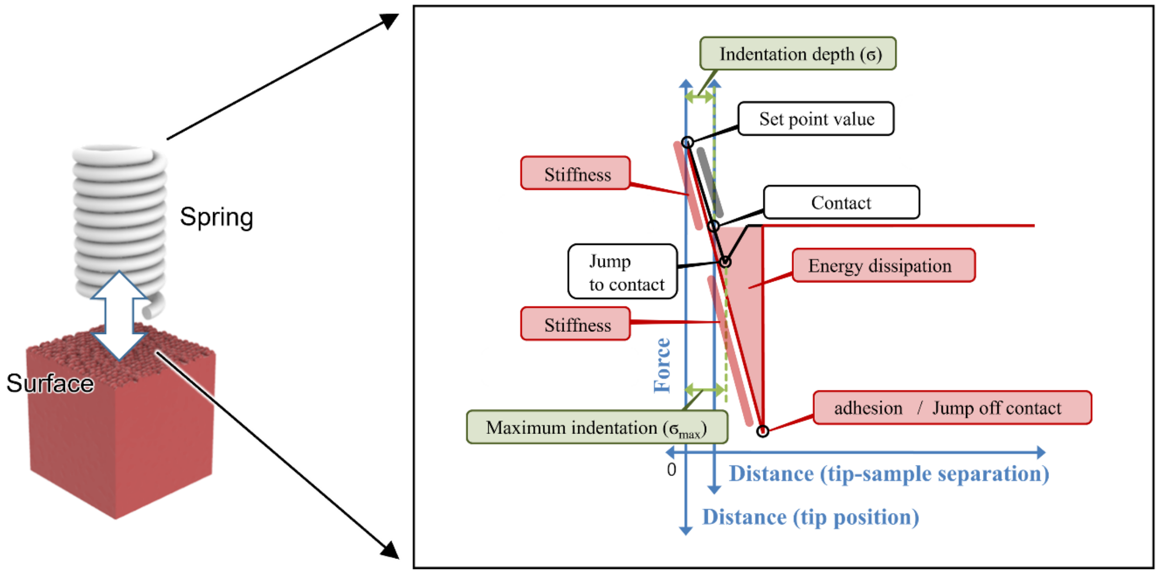


**Supplementary Figure 6** Schematic ideal image for the force-distance curve measured on an AFM system.

Using various setpoints of 20, 30, 50, 70, and 100 nN, we measured the elastic modulus of the DNTT film (i.e., without sidechain modification), as shown in Supplementary Figure 7 (a). In the approach force curves, consistent results were obtained regardless of whether the DMT or Hertz model was used. Similarly, when analyzing retract curves at setpoints below 50 nN, both models yielded comparable values. However, at setpoints of 50 nN and above, significant discrepancies emerged between the two models. These differences can be attributed to the increased adhesion forces in the retract curves, which the DMT model accounts for during fitting. Additionally, for setpoints above 50 nN, the elastic modulus values derived from the approach curves also showed a noticeable increase.

Ideally, Young's modulus, being an intrinsic property, should remain constant regardless of the applied setpoint. However, at the nanoscale, variations in measured values can arise due to increased contact area, tip–sample interactions, or adhesion effects, especially at higher setpoints. In this context, the good agreement observed between the Hertz and DMT models at 20 and 30 nN is noteworthy, whereas significant deviations at higher setpoints suggest additional influencing factors beyond the purely elastic response.

To verify the absence of plastic deformation during indentation, topographical image analysis was conducted. As shown in Supplementary Figure 7 (c), no evidence of plastic indentation was observed at any setpoint. Therefore, it is considered appropriate to analyse the elastic properties of DNTT using the approach curve.

Additionally, the largest image shift occurred at 70 nN, likely due to lateral forces acting at this setpoint. From this perspective, the point where the lateral stress is highest corresponds to the highest setpoint at which the indentation depth no longer increases, exhibiting a behaviour similar to saturation.

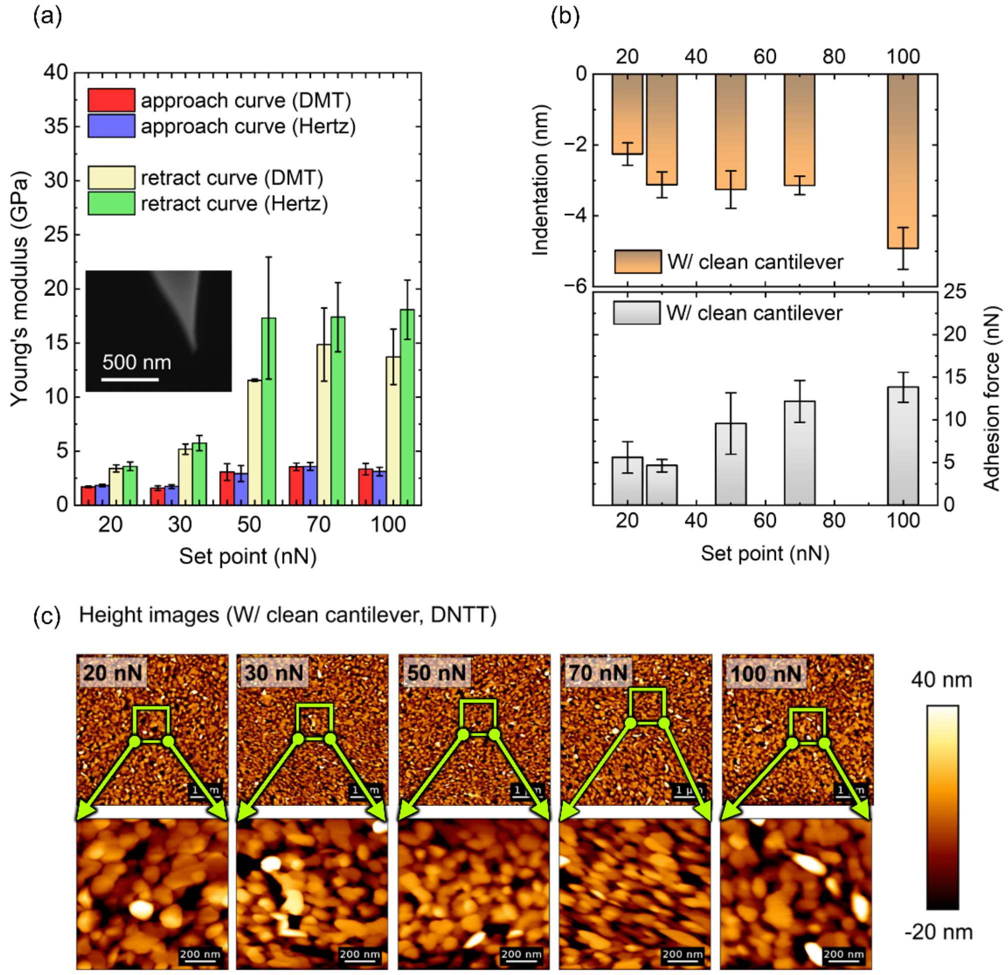


**Supplementary Figure 7** (a) Elastic modulus of DNTT measured at various setpoints, (b) nanoindentation behaviour, and adhesion force. (c) Absence of plastic deformation and penetration features in the height map.

As shown in Supplementary Figure 7 (b), with setpoints exceeding 50 nN, the indentation depth did not increase simultaneously with the increase in setpoints. It showed a similar indentation depth at 30, 50, and 70 nN, but significantly increased at 100 nN, as shown in Supplementary Figure 7 (b). Supplementary Figure 7 (b) shows the adhesion force measured as a function of setpoint. An interesting observation is that, according to contact mechanics, the contact radius can be expressed as $\sqrt{Rd}$, where $R$ is the tip radius and $d$ is the indentation depth. This leads to an estimated contact area of $\pi Rd$, suggesting that the adhesion force should be proportional to the contact area. However, the observed trend deviates from this expectation. Additionally, the indentation depth did not consistently increase with increasing setpoint, which also contradicts the anticipated behaviour.

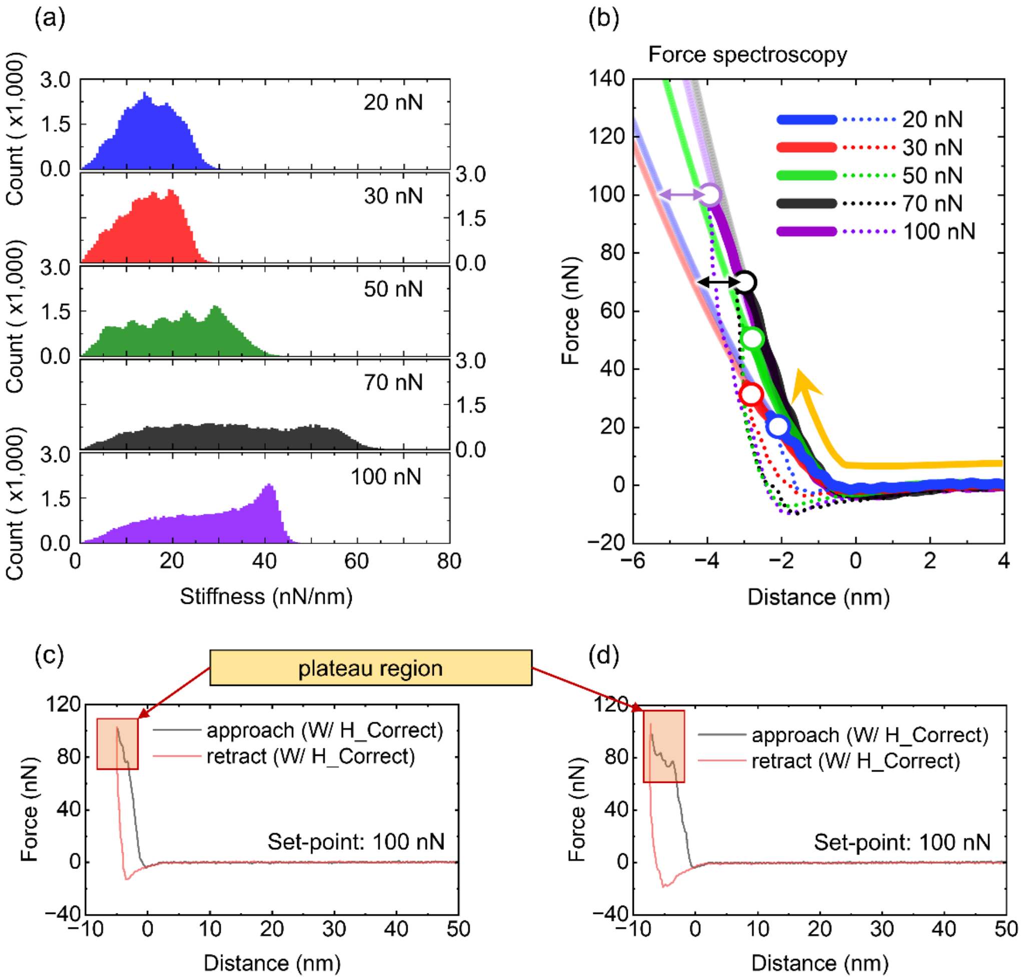


**Supplementary Figure 8** (a) Stiffness histograms by setpoint. (b) Force-spectroscopy analysis based on average Young's modulus. (c) and (d) plateau region at 100 nN setpoint.

In Supplementary Figure 8 (a), the stiffness histogram obtained from the approach curves corresponding to the Hertz model shows that the setpoints of 20 nN and 30 nN exhibit a typical Gaussian distribution. However, this trend breaks down at setpoints above 50 nN, where a tendency toward higher stiffness values emerges. This shift becomes more

pronounced as the setpoint increases from 50 nN to 70 nN. Although the trend appears to diminish at 100 nN, the distribution shows an increase around 40 nN/nm.

In addition, as shown in Supplementary Figure 8 (b), force–distance curve analysis was conducted based on the average Young's modulus. The solid lines represent the approach curves at each setpoint, while the dashed lines correspond to the retraction curves. The lighter lines indicate the fitted curves using the Hertz model. The fitting was based on the following equation:

$$F = \frac{4}{3}E^{*}\sqrt{R}\delta^{3/2}$$

where $F$ is the force, $E^{*}$ is the effective Young's modulus, $R$ is the tip radius, and $\delta$ is the indentation depth.

When using setpoints of 20 and 30 nN, the force–distance curves were highly similar under both conditions, consistent with the Young's modulus analysis shown in Supplementary Figure 7 (a) and 8 (b). In contrast, at 50, 70, and 100 nN, the force–distance curves exhibited much steeper slopes. Interestingly, while a typical densification effect often presents as an initial elastic response at low force followed by a steeper slope at higher forces, this behaviour was not observed in the cases where a higher Young's modulus was measured—namely, the 50, 70 and 100 nN setpoints. Additionally, at the 100 nN setpoint, the force–distance curves exhibited a slight decrease in slope within the high-force region, resembling the characteristic plateau typically associated with plastic deformation, as shown in Supplementary Figures 8 (c) and (d). This observation is highly unusual and contradicts the absence of plastic deformation indicated by the height map analysis in Supplementary Figure 7 (c).

In general, two possible scenarios can be considered. First, when using a low setpoint, the probe may interact only with a very shallow surface region, which may not sufficiently reflect the material's general elastic properties. Second, at high setpoints, effects such as densification, penetration, or plastic deformation may occur, making it difficult to isolate the elastic response. However, if the observed deviations were merely due to measurement errors at low setpoints, they would not account for the consistent indentation depths seen in Supplementary Figure 7 (b) and 8 (b), where the indentation depth remains nearly unchanged between 30 and 70 nN. Furthermore, the shift toward higher stiffness values in the histogram shown in Supplementary Figure 8 (a) indicates that the apparent increase in modulus at higher setpoints may not be solely due to elastic stiffening. In particular, the absence of a characteristic densification-related curvature in the force–distance curves showing high modulus at high setpoints suggests that there may be additional factors at play that warrant further consideration.

To gain deeper insight, additional force-spectroscopy analysis was performed using the Python script described in Section VI. The Hertz model was applied to both the full and partial slope regions, as changes in slope (or Young's modulus) directly reflect variations in mechanical properties. This is particularly useful for identifying nonelastic behaviours such as plastic deformation or densification, where regional slope differences can indicate elastic limits, structural changes, or internal stress distributions.

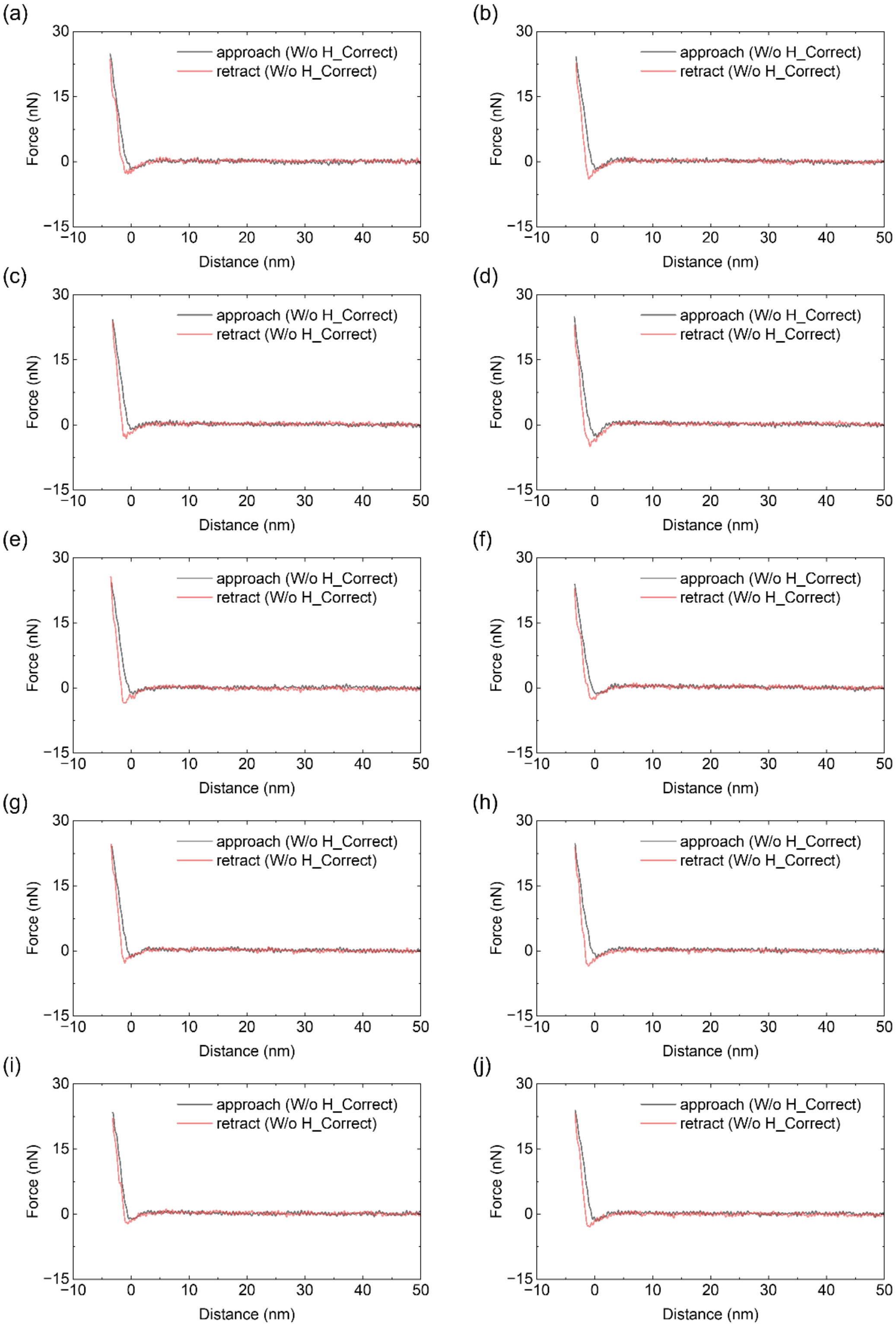


**Supplementary Figure 9** Force–distance curves at a setpoint of 20 nN (without height correction).

The force–distance curves obtained at a setpoint of 20 nN are shown in Supplementary Figure 9. A slight hysteresis was observed between the approach and retraction curves, but it was not significant. In these measurements, the 'distance' represents the

displacement of the z-directional step motor relative to the jump-to-contact point, with the maximum indentation used to construct the force–distance curve. During this process, the 'height' must be corrected for cantilever bending to accurately reflect the actual tip position.

To aid understanding, Supplementary Figure 10 provides an additional explanation of the 'height correction' process. In a typical AFM system, determining the actual tip height during indentation involves identifying the 'jump-to-contact' or 'contact' point ($z_0$ or $z_0'$), as illustrated in panels (a) and (b) of Supplementary Figure 10. This point is set as the zero height, and subsequent movement of the z-motor (or the z-direction movement of the AFM system) is corrected for cantilever bending, which occurs as the tip is indented into the surface until the predefined setpoint force is reached. This correction allows for accurate determination of the actual tip position during indentation.

The distance values in the force–distance curves shown in Supplementary Figure 11 were corrected accordingly. Height correction, which compensates for cantilever bending relative to the movement of the cantilever holder, was automatically performed using the JPK SPM Data Processing Software v.6.1.102.

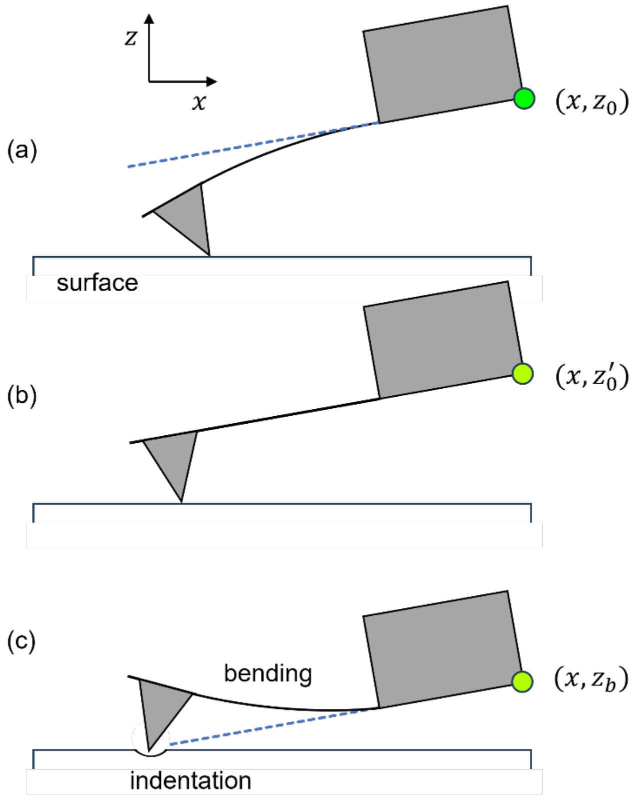


**Supplementary Figure 10** Schematic of ideal height correction with exaggerated deformation to illustrate the effect.

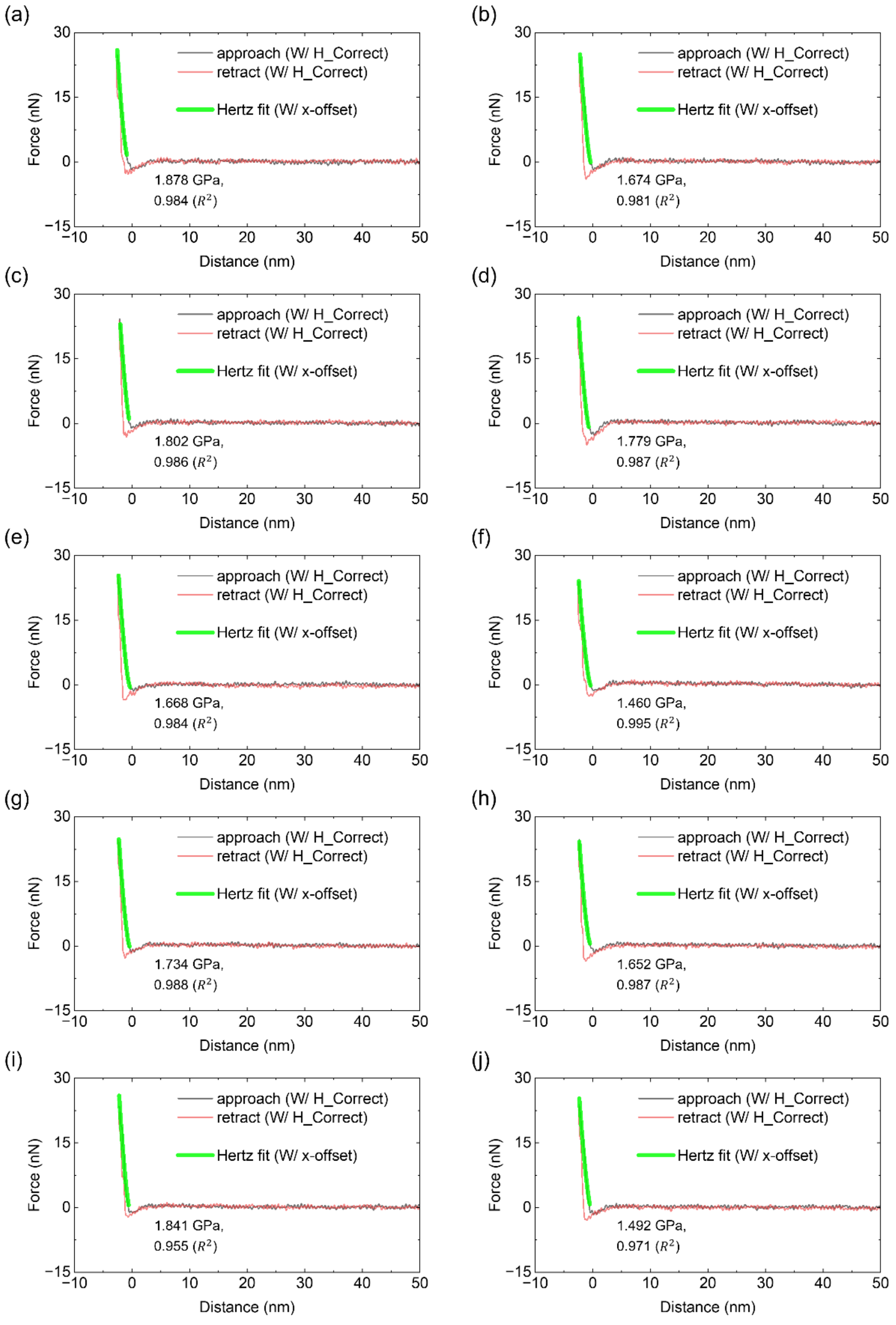


**Supplementary Figure 11** Force–distance curves at a setpoint of 20 nN (with height correction).

The force-distance curves in Supplementary Figure 11 (a)~(j) correspond to the respective curves in Supplementary Figure 9 (a)~(j) after undergoing the "Height correction" process. The regions highlighted in light green indicate the portions where fitting was performed using the aforementioned Python script.

Each curve shows a Young's modulus ranging from approximately 1.46 to 1.88 GPa, consistent with the average values obtained through QI mapping. As clearly illustrated in Supplementary Figure 11, no typical signs of plastic deformation were observed during the indentation process.

To assess the quality of the fitting, the coefficient of determination factor ($R^2$) was used. The $R^2$ is a statistical measure used in regression analysis to evaluate how well a model explains the variability of the observed data. Its value typically ranges from 0 to 1. An $R^2$ value of 1 indicates that the model perfectly fits the data, meaning all data points lie exactly on the regression line. An $R^2$ value of 0 means the model fails to explain any of the variability in the data, essentially performing no better than using the mean as a predictor. Values between 0 and 1 indicate that the model explains part of the variation in the data, with higher values reflecting a better fit. This $R^2$ is defined as $R^2 = 1 - \frac{SS_{res}}{SS_{tot}}$ where $SS_{res}$is the sum of squared residuals (i.e., the difference between the observed and predicted values, $SS_{res} = \sum(y_i - \hat{y_i})^2$), $SS_{tot}$ is the total sum of squares (i.e., the variance of the observed data relative to its meaning, $SS_{tot} = \sum(y_i - \bar{y})^2$). This formulation quantifies the proportion of the total variation in the data that is captured by the model.

Fitting was conducted by varying the x-offset around the contact point, with adjustments made in steps of approximately 0.05 nm to achieve the best match. The table below illustrates how the fitting results change with and without applying an x-offset, showing the effect of contact point modification. In this case, the contact point was shifted in increments of approximately 0.16 nm during the fitting process.

| W/o x-offset function | | | W/ x-offset function | | |
|---|---|---|---|---|---|
| contact point modification (nm) | Relative change from the unmodified case (%) | coefficient of determination ($R^2$) | contact point modification (nm) | Relative change from the unmodified case (%) | coefficient of determination ($R^2$) |
| 0.32 | 70.88 | 0.98 | 0.32 | 90.36 | 0.99 |
| 0.16 | 86.54 | 0.99 | 0.16 | 90.36 | 0.99 |
| 0 | 100.00 | 0.98 | 0 | 100.00 | 0.98 |
| -0.16 | 107.04 | 0.98 | -0.16 | 100.00 | 0.98 |
| -0.32 | 133.15 | 0.93 | -0.32 | 98.24 | 0.98 |

**Supplementary Table 2** Analysis of fitting accuracy with respect to x (distance)

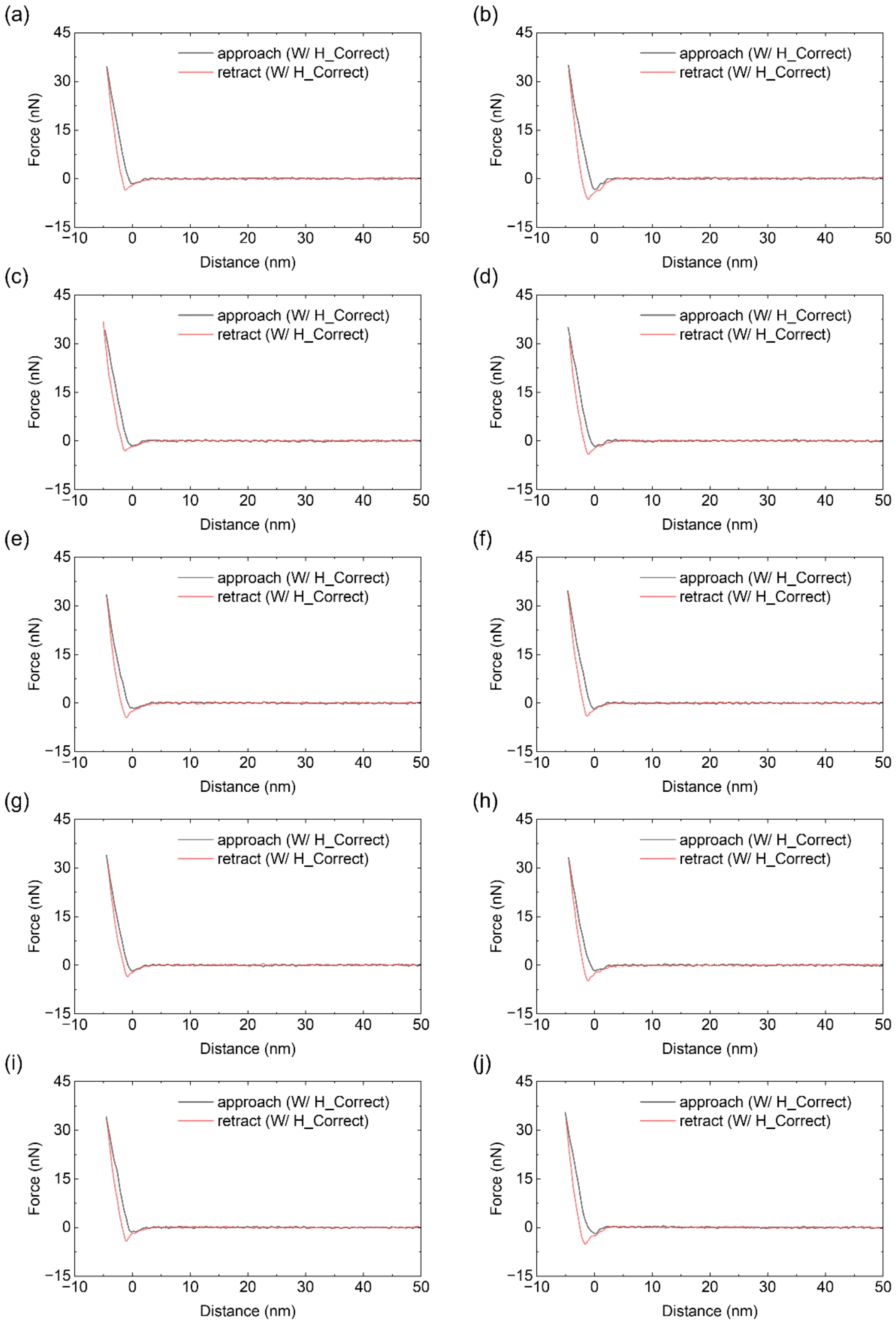


**Supplementary Figure 12** Force–distance curves at a setpoint of 30 nN (without height correction).

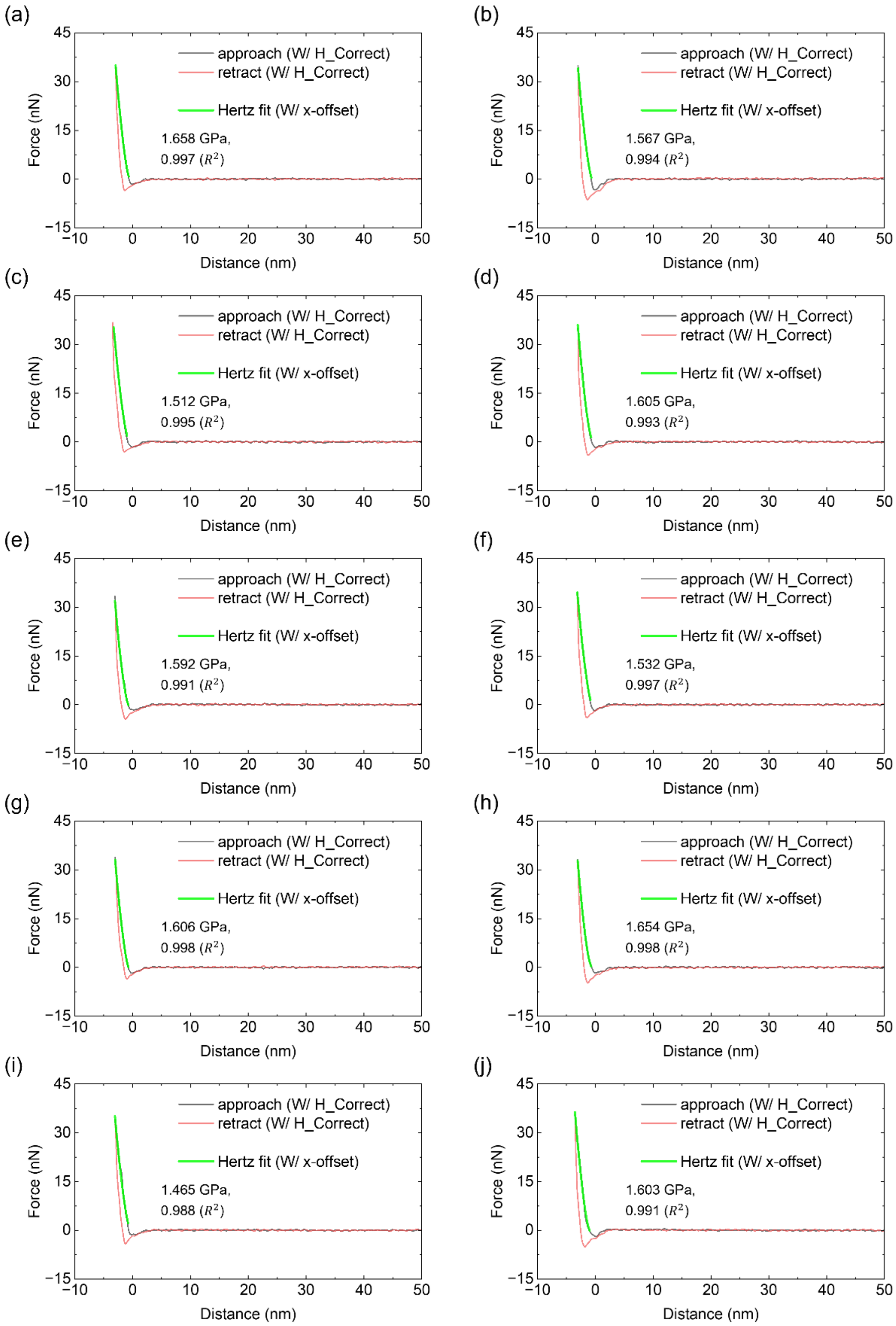


**Supplementary Figure 13** Force–distance curves at a setpoint of 30 nN (with height correction).

For a setpoint of 30 nN, the graphs without and with height correction are shown in Supplementary Figures 12 and 13, respectively. In both figures, panels (a) through (j) correspond to each other. No slope indicative of plastic deformation was observed in either figure. The Young's modulus ranged from approximately 1.47 to 1.66, and the $R^2$ values were between 0.99 and 1.00, which are slightly higher than those obtained from the force-distance curve fitting using the 20 nN setpoint.

The Hertz model fitting was performed in the same manner as for the 20 nN setpoint curves. Additionally, the accuracy of the fitting was evaluated by applying and not applying the x-offset. The force-distance curve used for this analysis corresponds to Supplementary Figure 13 (a). When the x-offset was not applied, the results showed up to a 33% difference with the 20 nN setpoint, whereas with the 30 nN setpoint, the difference was reduced to approximately 21%, indicating a slight decrease in variation.

Based on the Hertz model fitting of the force-distance curves obtained at 20 and 30 nN setpoints, the slight differences observed in the statistical QI analysis may be attributed to fitting errors. This is possibly due to the relatively smaller indentation depth at the 20 nN setpoint, which results in a narrower fitting range compared to the 30 nN case.

| W/o x-offset function | | | W/ x-offset function | | |
|---|---|---|---|---|---|
| contact point modification (nm) | Relative change from the unmodified case (%) | coefficient of determination ($R^2$) | contact point modification (nm) | Relative change from the unmodified case (%) | coefficient of determination ($R^2$) |
| 0.32 | 85.82 | 1.00 | 0.32 | 94.15 | 1.00 |
| 0.16 | 95.41 | 0.99 | 0.16 | 100.00 | 1.00 |
| 0 | 100.00 | 0.98 | 0 | 100.00 | 1.00 |
| -0.16 | 109.38 | 0.97 | -0.16 | 104.28 | 1.00 |
| -0.32 | 121.37 | 0.96 | -0.32 | 108.81 | 0.99 |

**Supplementary Table 3** Analysis of fitting accuracy with respect to x (distance)

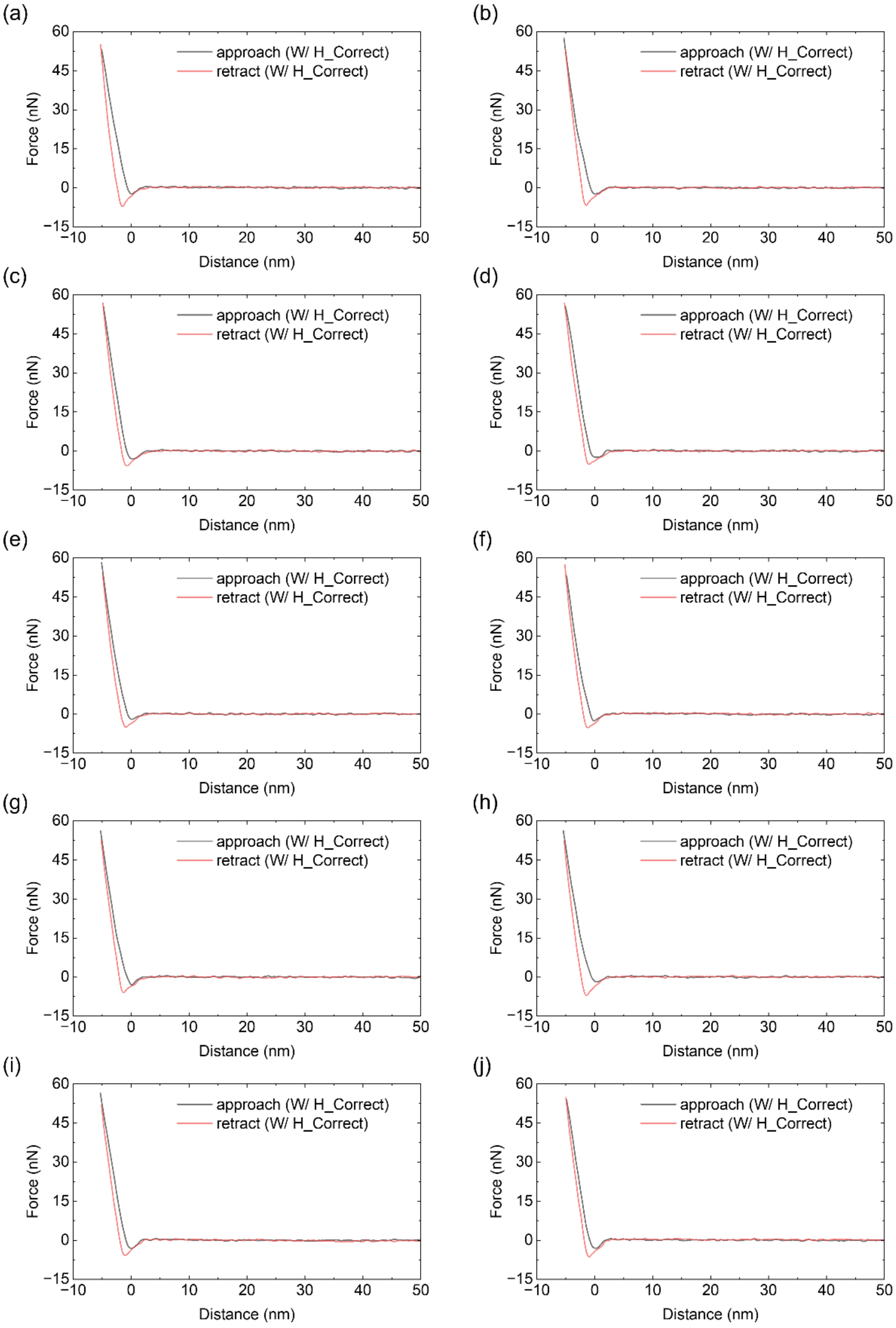


**Supplementary Figure 14** Force–distance curves showing high modulus at a setpoint of 50 nN (without height correction).

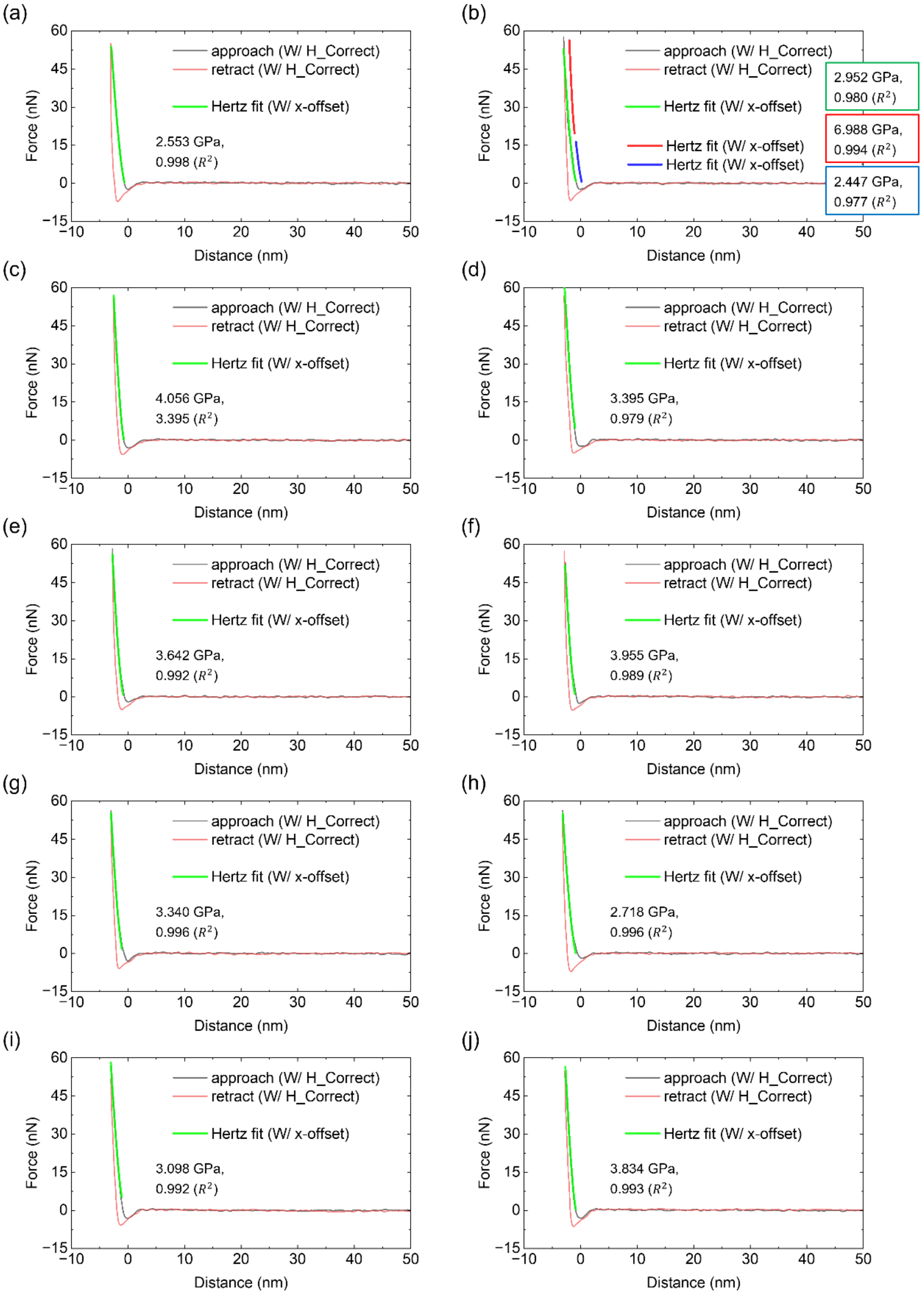


**Supplementary Figure 15** Force–distance curves showing high modulus at a setpoint of 50 nN (with height correction).

As with the previous force-distance curves, Supplementary Figures 14 and 15 are based on essentially the same data, differing only in whether height correction was applied. Each panel from (a) to (j) in Supplementary Figure 14 corresponds one-to-one with panels (a) to (j) in Supplementary Figure 15.

Supplementary Figures 14 and 15 present the force-distance curves obtained at a setpoint of 50 nN. In this case, the measured Young's modulus values were generally higher than those obtained at 20 and 30 nN setpoints. None of the curves showed typical features of plastic deformation.

Interestingly, Supplementary Figure 15 (b) showed a slope somewhat resembling densification with an $R^2$ of 0.980, a tendency also seen in Supplementary Figure 14 (b). Although the deformation is subtle and not definitively attributed to densification, partial fitting was performed for careful analysis. In this case, Hertz model fitting of the overall slope yielded a Young's modulus of about 2.96 GPa. In the lower blue region—where intrinsic modulus would be expected if densification occurred—the modulus remained relatively high at around 2.45 GPa, while the following red region showed a significant increase to about 6.99 GPa. The similarity between the overall and blue region values suggests that, despite slight visual deformation at 15 nN and 20 nN loading, the deformation is unlikely to be strongly influenced by densification.

Overall, the force-distance curves at the 50 nN setpoint, despite showing high modulus values, did not exhibit the typical behavior expected from densification. Instead, most curves displayed slope characteristics similar to those of an elastic material.

The Hertz model fitting at the 50 nN setpoint was performed using the same method as in the previous cases. To further assess the accuracy of the fitting and to gain additional insights into the slope characteristics, a summary table was provided. From Supplementary Figures 15 (a), without applying the x-offset, the maximum fitting error reached approximately 27%. However, when the x-offset was applied, the $R^2$ values were consistently close to 1, with minimal variation.

| W/o x-offset function | | | W/ x-offset function | | |
|---|---|---|---|---|---|
| contact point modification (nm) | Relative change from the unmodified case (%) | coefficient of determination ($R^2$) | contact point modification (nm) | Relative change from the unmodified case (%) | coefficient of determination ($R^2$) |
| 0.32 | 89.95 | 1.00 | 0.32 | 100.00 | 1.00 |
| 0.16 | 100.00 | 0.99 | 0.16 | 100.00 | 1.00 |
| 0 | 100.00 | 0.99 | 0 | 100.00 | 1.00 |
| -0.16 | 114.64 | 0.97 | -0.16 | 107.68 | 1.00 |
| -0.32 | 127.42 | 0.96 | -0.32 | 107.68 | 1.00 |

**Supplementary Table 4** Analysis of fitting accuracy with respect to x (distance)

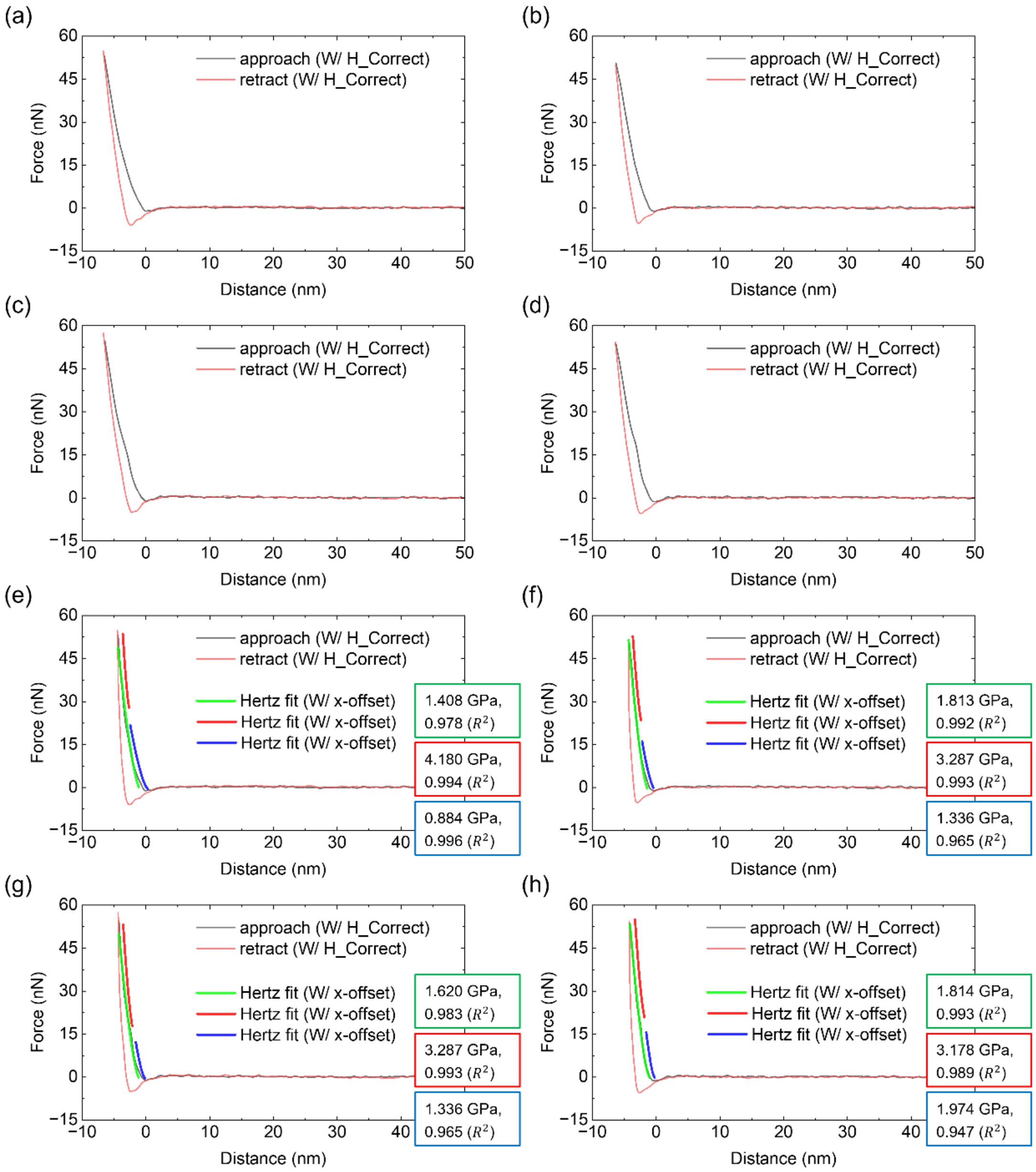


**Supplementary Figure 16** Force–distance curves showing a modulus of approximately 1.4~1.9 GPa at a setpoint of 50 nN: (a)~(d) without height correction, (e)~(f) with height correction.

To approach the analysis more cautiously, the possibility and indications of densification effects were examined by analysing slopes in the range of approximately 1.4~1.9 GPa at a 50 nN setpoint, as shown in Supplementary Figure 16. Panels (a) through (d) correspond to panels (e) through (h), respectively, with the only difference being the application of height correction. Since multiple fittings are involved in this analysis, both sets of graphs are presented to allow for a clearer comparison of slope trends.

While the overall slope fitting yielded high $R^2$ values, all above 0.97, slight variations were observed, prompting a more comprehensive analysis. In particular, the difference between the lower region and the full slope region was investigated, as it may represent

the most significant variation. Segmental analysis is critical for evaluating where the deformation begins and how it progresses—insights that cannot be fully captured by a single $R^2$ value or average modulus. This approach is especially useful for detecting subtle structural changes that may indicate early signs of densification.

The differences between the full slope region and the lower region (the range in which Young's modulus is expected to be accurate before any densification occurs) were 0.52, 0.70, 0.28, and 0.16 GPa, respectively. These relatively small differences make it difficult to confidently attribute the slope variations to densification effects. Moreover, since the forces at which densification is presumed to occur are below 30 nN, it is hard to justify the appearance of densification in the force–distance curves measured at a 50 nN setpoint, especially given that no such behaviour was observed in the curves shown in Supplementary Figures 12 and 13.

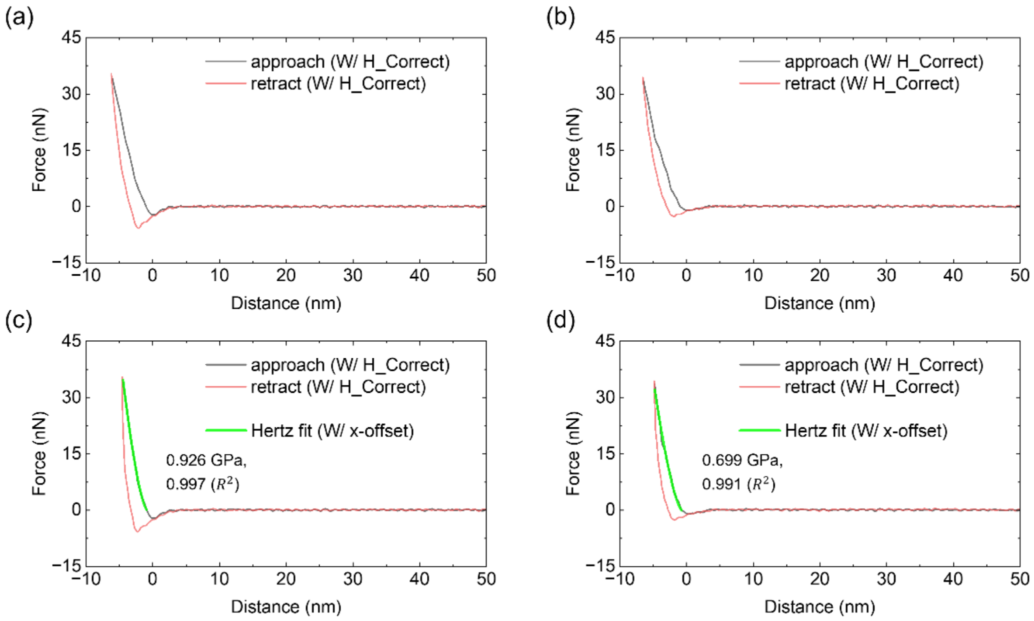


**Supplementary Figure 17** Force–distance curves showing a modulus of approximately 0.7~0.9 GPa at a setpoint of 30 nN: (a) and (b) without height correction, (c) and (d) with height correction.

The force–distance curve obtained at a 30 nN setpoint, as shown in Supplementary Figure 17, exhibited relatively low modulus values. If the curves discussed in Supplementary Figure 17 were indeed influenced by densification, one would expect to observe precursor signs—such as slight plastic deformation—starting at around 15~20 nN and becoming evident by 30 nN. However, no such features were detected at the 30 nN setpoint, making it difficult to attribute the changes observed at 50 nN to densification.

It is possible that such distinct features may not appear clearly in non-porous materials. However, in the force–distance curve shown in Supplementary Figure 15, measured at a 50 nN setpoint, even in the low loading force region (0 to 20 or 30 nN) starting from the contact point, the slope—and consequently the modulus value—are already higher

compared to those measured at 20 or 30 nN setpoints in Supplementary Figures 9, -5, -6, and -7. This is a particularly unusual and noteworthy observation.

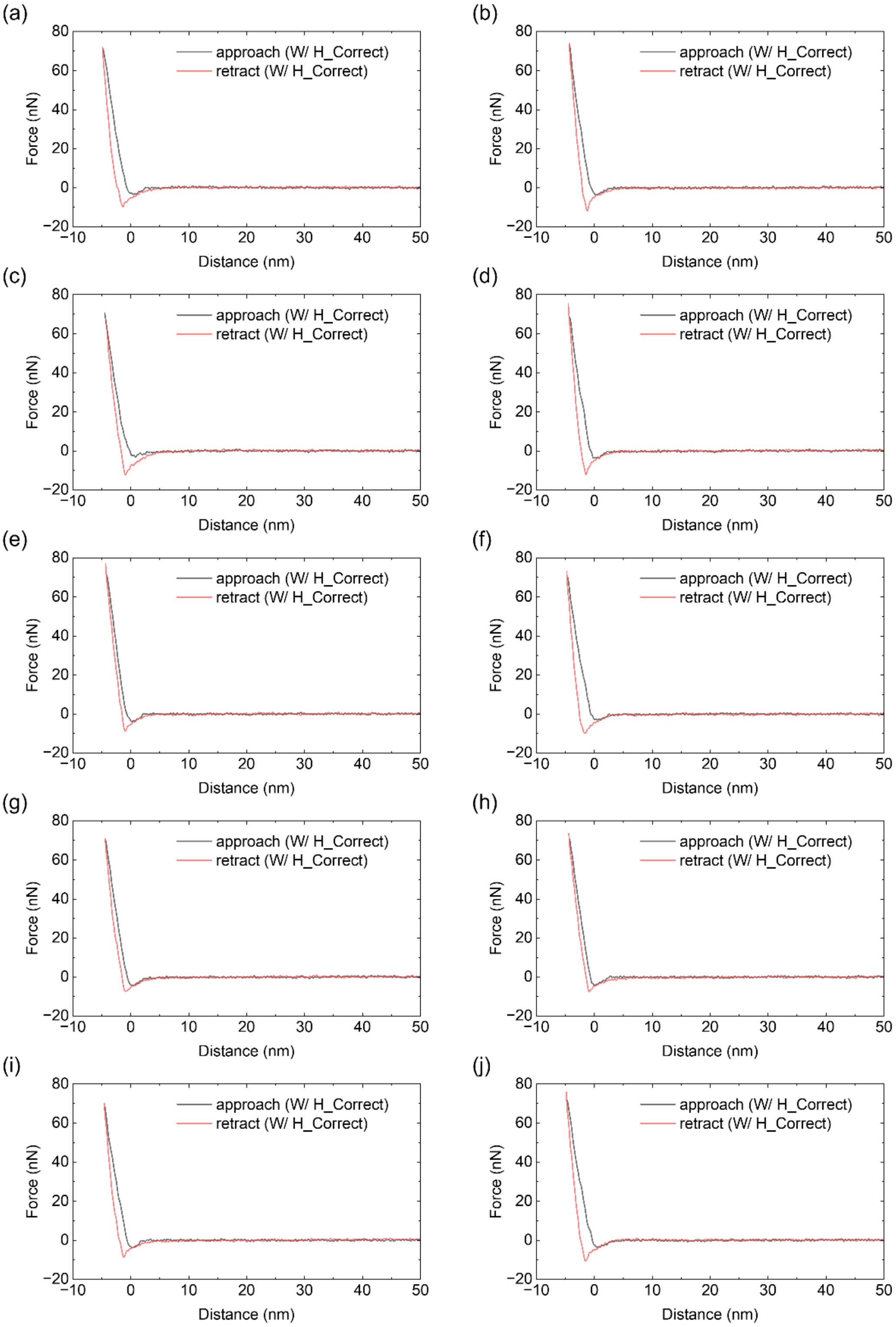


**Supplementary Figure 18** Force–distance curves showing high modulus at a setpoint of 70 nN (without height correction).

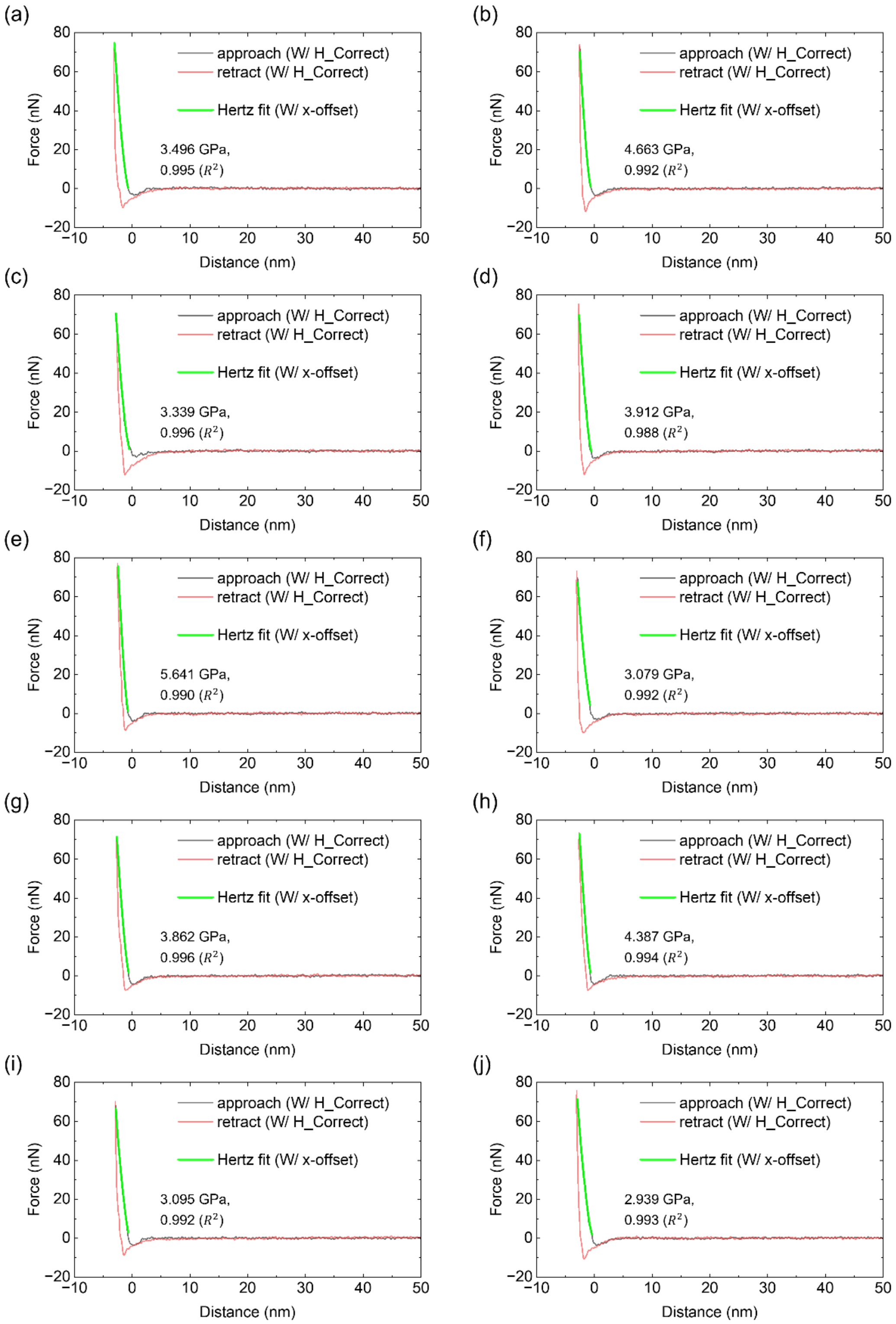


**Supplementary Figure 19** Force–distance curves showing high modulus at a setpoint of 70 nN (with height correction).

Supplementary Figures 18 and 19 show the force–distance curves obtained at a 70 nN setpoint, exhibiting Young's modulus values ranging from approximately 2.9 to 5.6 GPa. In panels (b), (f), and (j) of each figure, slight fluctuations in slope were observed at forces below 30 nN. However, as with the analysis of the force–distance curves at the 50 nN setpoint, these variations were not sufficient to suggest the presence of densification or similar effects.

As with the measurements at the 70 nN setpoint, the absence of typical deformation features—such as those associated with densification—combined with the elastic-like shape of the force–distance curves, presents a particularly interesting result.

Additional slope evaluations were conducted using Supplementary Figure 19 (a) obtained at a 70 nN setpoint, and the results are summarized in the following table. When the x-offset was not applied, the maximum percentage change observed was approximately 19%. When the x-offset was applied, the fittings consistently yielded very high $R^2$ values across the board.

| W/o x-offset function | | | W/ x-offset function | | |
|---|---|---|---|---|---|
| contact point modification (nm) | Relative change from the unmodified case (%) | coefficient of determination ($R^2$) | contact point modification (nm) | Relative change from the unmodified case (%) | coefficient of determination ($R^2$) |
| 0.32 | 81.19 | 1.00 | 0.32 | 96.57 | 1.00 |
| 0.16 | 81.19 | 1.00 | 0.16 | 96.57 | 1.00 |
| 0 | 100.00 | 0.99 | 0 | 100.00 | 1.00 |
| -0.16 | 100.91 | 0.99 | -0.16 | 109.61 | 0.99 |
| -0.32 | 117.15 | 0.97 | -0.32 | 109.87 | 0.99 |

**Supplementary Table 5** Analysis of fitting accuracy with respect to x (distance)

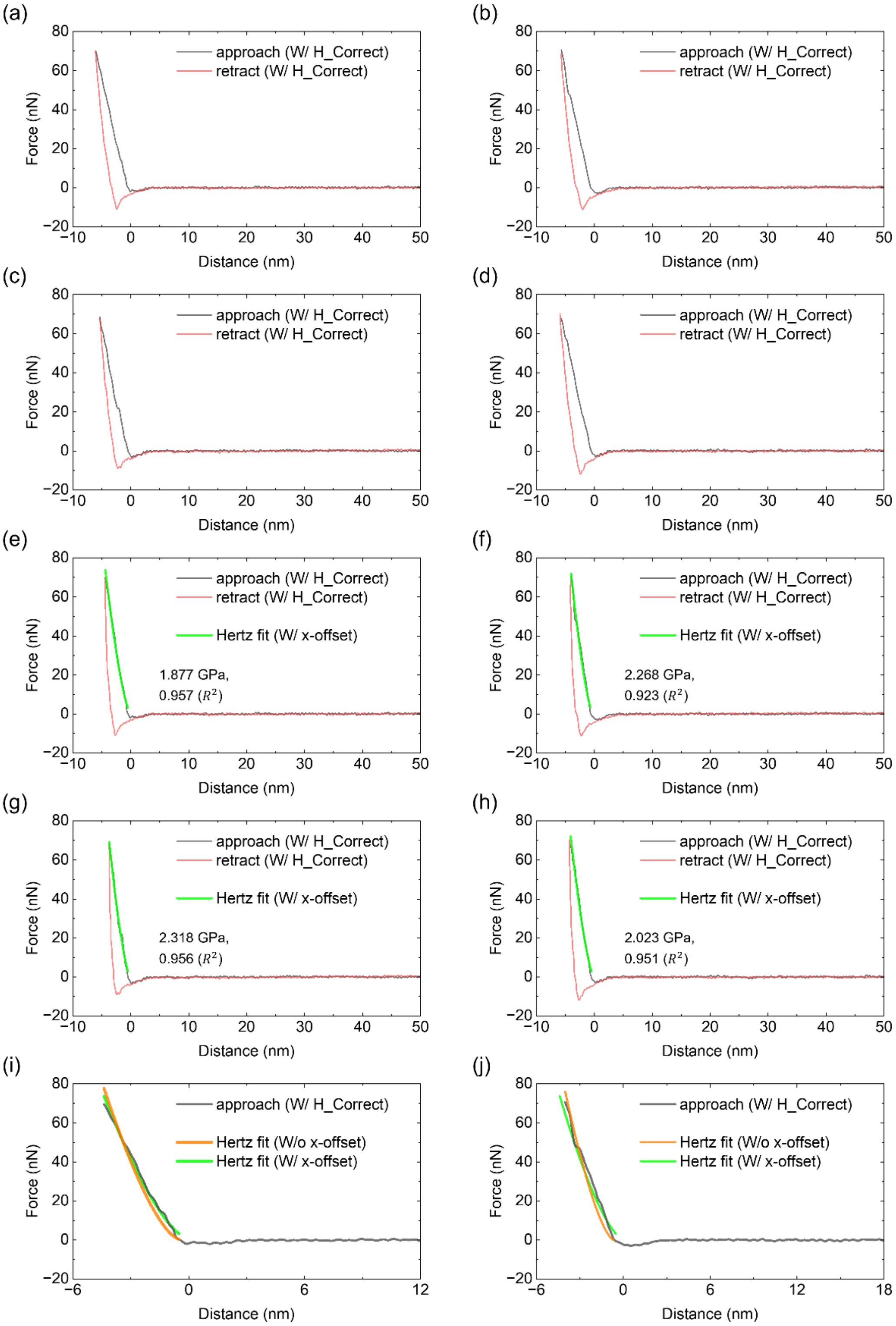


**Supplementary Figure 20** Force–distance curves showing a modulus of approximately 1.5~2.0 GPa at a setpoint of 70 nN: (a)~(d) without height correction, (e)~(h) with height correction. (i) and (j) are rescaled versions of the graphs shown in (e) and (f), which include fittings performed without x-offset adjustment.

In Supplementary Figure 20, the force–distance curves measured at a 70 nN setpoint exhibited distinctly unusual behavior, with Young's modulus values ranging from approximately 1.9 to 2.3 GPa. The overall slope of the curves appeared highly linear. Although the $R^2$ values ranged from 0.92 to 0.96, suggesting that Hertz model fitting was moderately feasible, the actual fitting accuracy was relatively low. This discrepancy was especially evident in the low-force region, where the curves maintained linearity in contrast to the expected nonlinearity characteristic of the Hertz model. This raises concerns about the reliability and appropriateness of the fitted values.

This linearity is more clearly demonstrated in panels (i) and (j), which are magnified views of panels (e) and (f), respectively. These panels compare fittings performed with and without x-offset correction. In the green curves (with x-offset correction), the x-values (distance) were adjusted to improve overall fitting accuracy, resulting in better curve alignment. However, this also caused the initial contact point to be significantly displaced from the actual curve. On the other hand, the orange curves (without x-offset correction) aligned well with the initial contact point, but the fitting diverged rapidly after that point due to the linear shape of the force–distance curves, which deviates from the typical nonlinear behavior expected from the Hertz model.

In panels (b), (c), and (d), slight slope variations were observed around 50 and 20 nN. However, due to the curve's overall linearity, the similarity of slopes across both low and high force regions, and the relatively poor fitting accuracy, partial fitting analysis was not suitable in this case.

Notably, this fitting behavior contrasts with the results from previous fittings performed at 20, 30, and 50 nN setpoints, where the $R^2$ values ranged from 0.98 to as high as 1.00, indicating much higher fitting accuracy. Furthermore, as demonstrated in Table 2 through 5 and Supplementary Figures 11, 13, 15, 16, and 19, x-offset fitting generally yielded curves that closely followed the expected shape. The case of Supplementary Figure 20, however, highlights the limitations of applying the Hertz model when the experimental data significantly deviate from its assumptions.

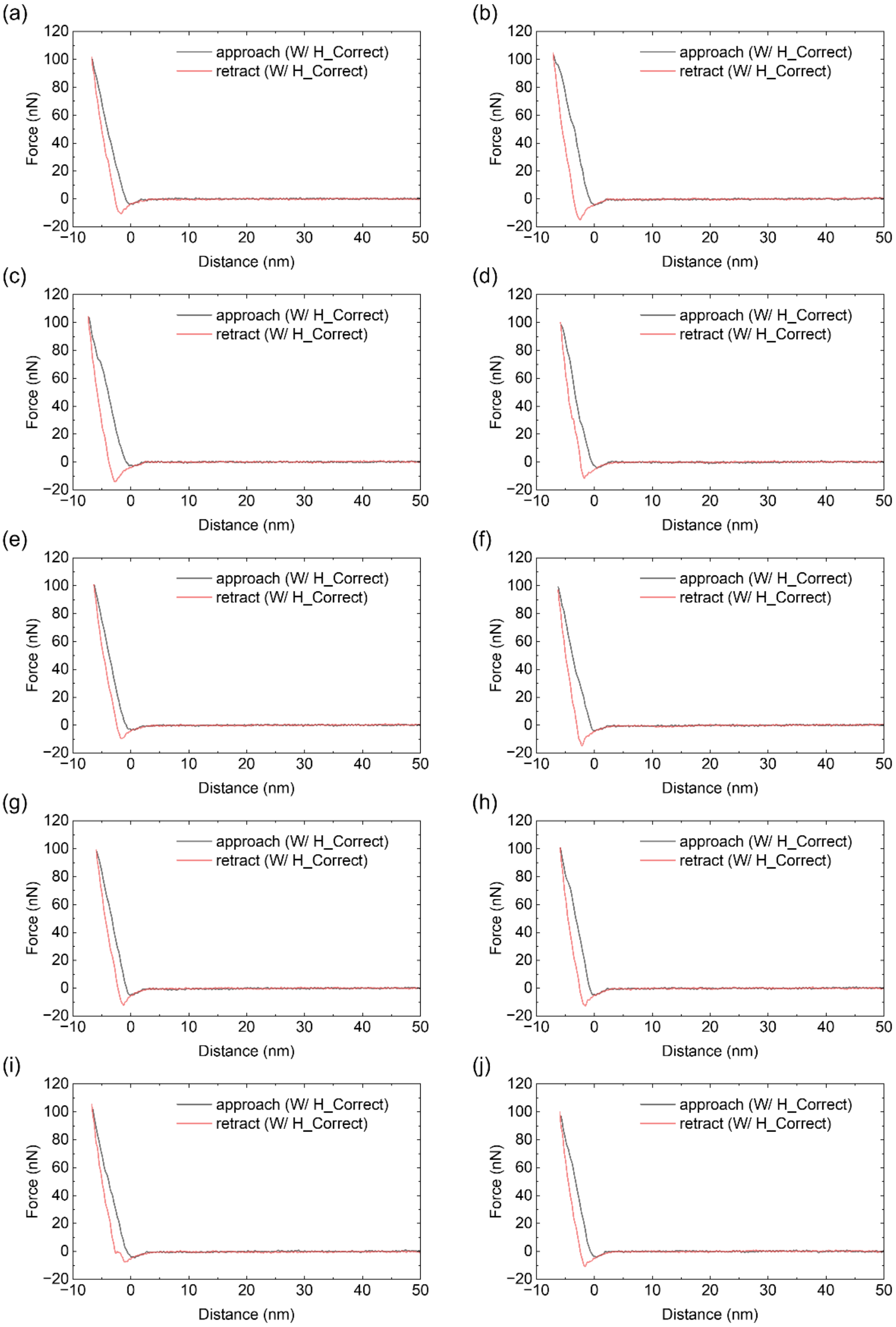


**Supplementary Figure 21** Force–distance curves showing high modulus at a setpoint of 100 nN (without height correction).

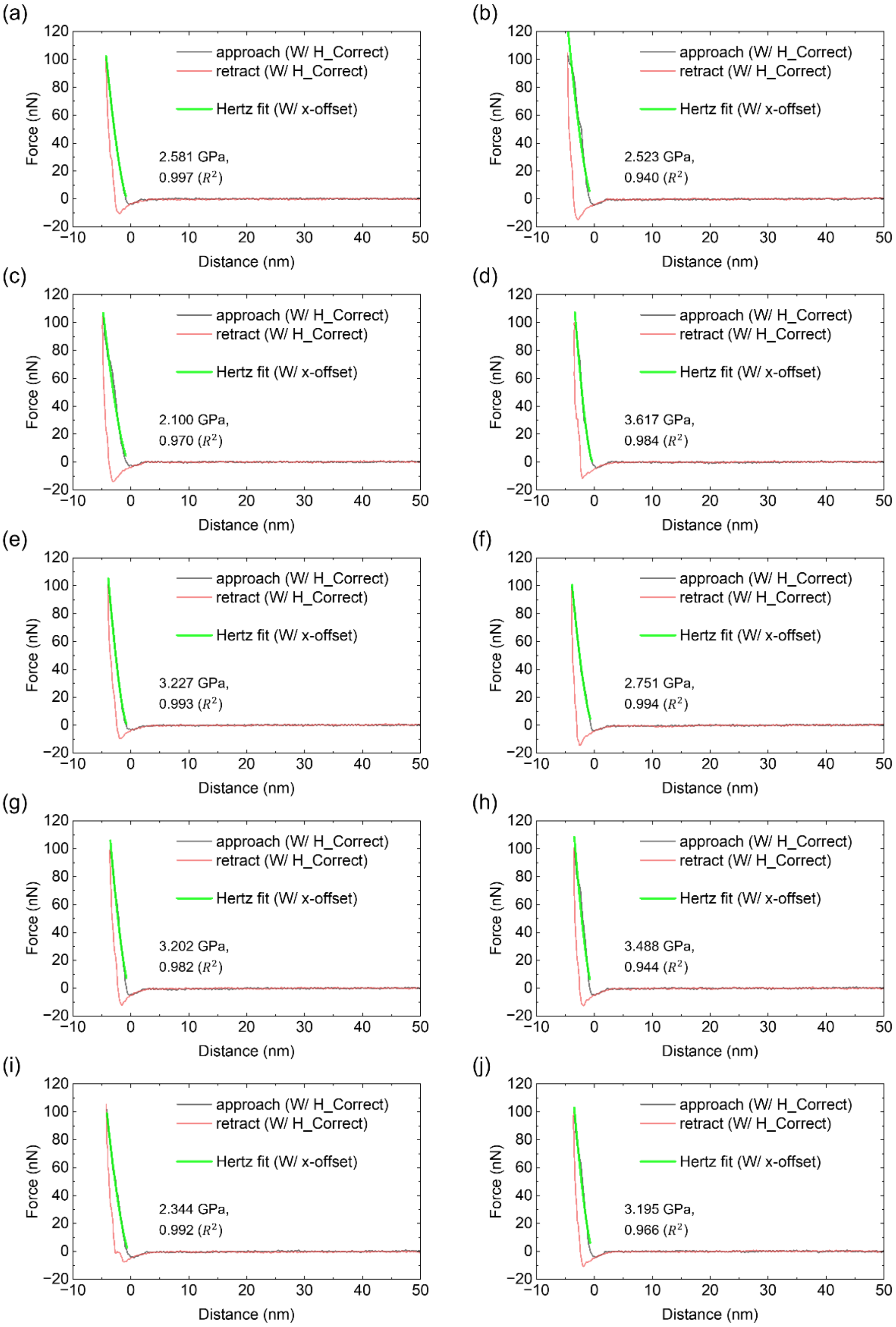


**Supplementary Figure 22** Force–distance curves showing high modulus at a setpoint of 100 nN (with height correction).

In Supplementary Figures 21 and 22, the force–distance curves obtained at a 100 nN setpoint exhibit Young's modulus values ranging from approximately 2.1 to 3.6 GPa. As in the previous analyses, the quality of the Hertz model fitting was assessed using the coefficient of determination ($R^2$). In the case of Supplementary Figure 22 (a), the fitting was found to be satisfactory, as shown in Supplementary Table 6.

However, as indicated in Supplementary Table 7, panels (b), (c), (h), and (j) showed $R^2$ values below 0.97, suggesting poor fitting accuracy. These cases, clearly visible in Supplementary Figure 21, exhibited significant fluctuations in distance beyond a certain force level in the force–distance curves. Although panel (f) also showed some fluctuation, appearing as if two segments were joined, it still resulted in a relatively high $R^2$ value, likely because the Hertz model is inherently nonlinear and accommodates such trends more easily.

An interesting observation across nearly all the graphs is that, although the degree varies, there appears to be a region in the high-force range where the distance increases disproportionately with the same rate of change in the applied loading force. This behavior resembles plastic deformation occurring within the same slope range. Such a trend is also evident in Supplementary Figure 23 (c) and (d). The cases of (b) and (h) are further discussed in Supplementary Figure 23.

Moreover, although the measured modulus values in this setpoint condition are higher compared to those at 20 and 30 nN setpoints, it was still very difficult to identify clear indications of densification—signals typically expected to influence such modulus increases.

| W/o x-offset function | | | W/ x-offset function | | |
|---|---|---|---|---|---|
| contact point modification (nm) | Relative change from the unmodified case (%) | coefficient of determination ($R^2$) | contact point modification (nm) | Relative change from the unmodified case (%) | coefficient of determination ($R^2$) |
| 0.32 | 84.44 | 0.96 | 0.32 | 96.90 | 1.00 |
| 0.16 | 92.01 | 0.97 | 0.16 | 97.48 | 1.00 |
| 0 | 100.00 | 0.98 | 0 | 100.00 | 1.00 |
| -0.16 | 82.92 | 0.99 | -0.16 | 105.77 | 1.00 |
| -0.32 | 86.17 | 1.00 | -0.32 | 108.83 | 1.00 |

**Supplementary Table 6** Analysis of fitting accuracy with respect to x (distance)

| | (a) | (b) | (c) | (d) | (e) | (f) | (g) | (h) | (i) | (j) |
|---|---|---|---|---|---|---|---|---|---|---|
| coefficient of determination **($R^2$)** | 1.00 | 0.94 | 0.97 | 0.98 | 0.99 | 0.99 | 0.98 | 0.94 | 0.99 | 0.97 |

**Supplementary Table 7** Coefficient of Determination ($R^2$) with X-Offset Adjustment

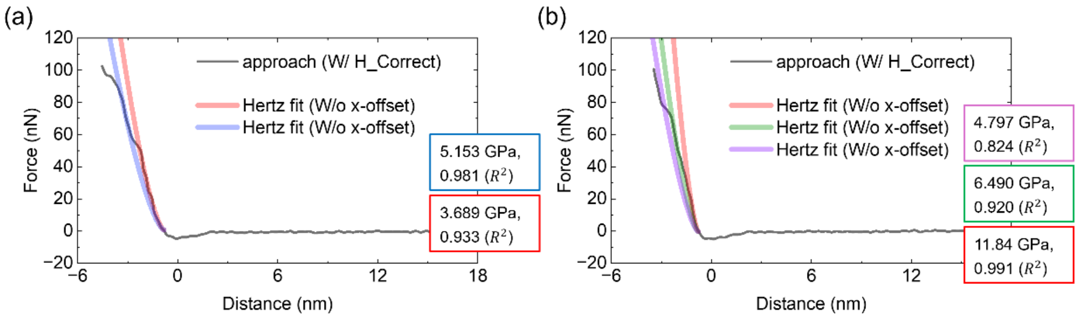


**Supplementary Figure 23** (a) and (b) are rescaled versions of Supplementary Figure 22 (b) and (h), respectively.

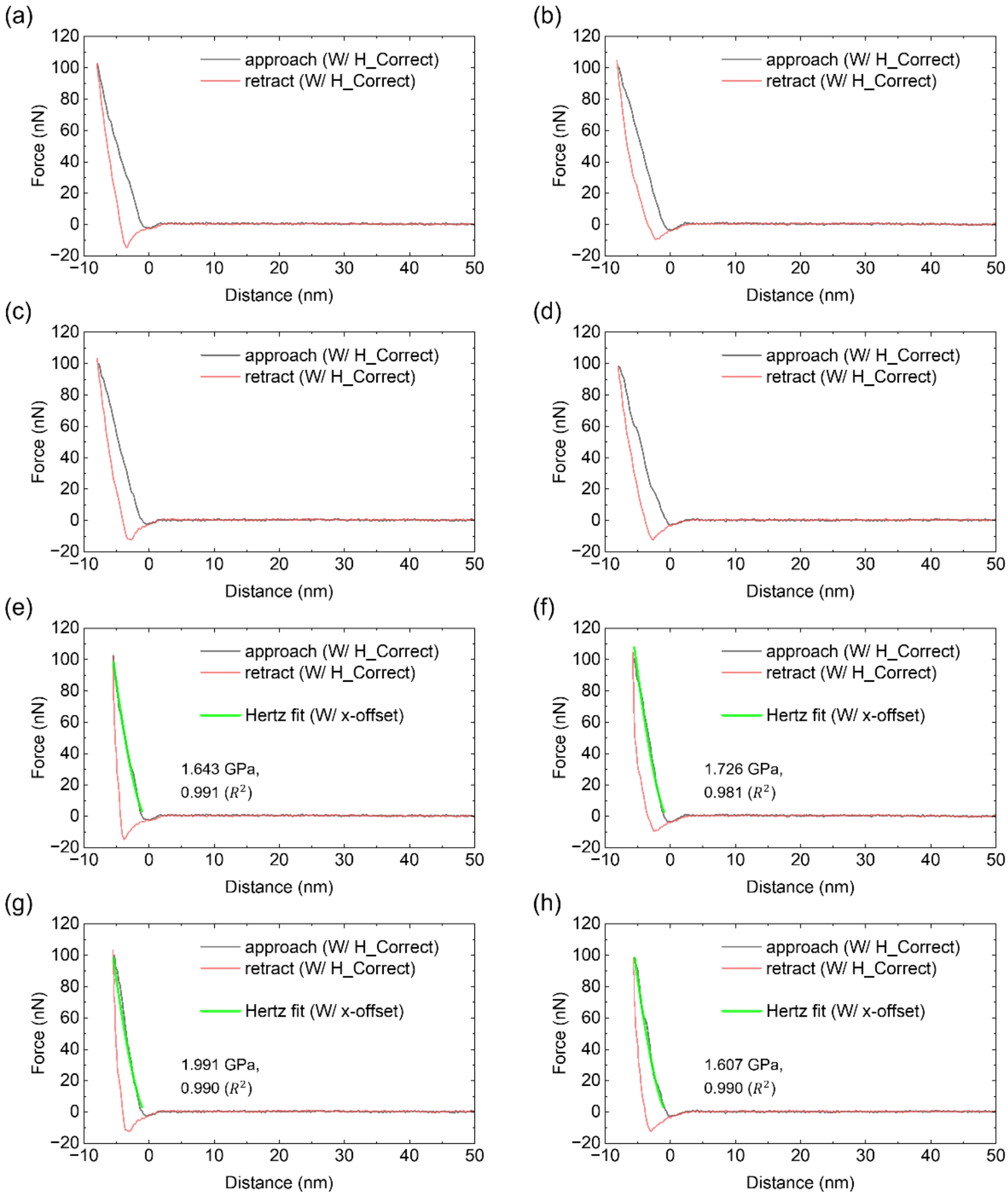


**Supplementary Figure 24** Force–distance curves showing a modulus of approximately 1.5~2.0 GPa at a setpoint of 100 nN: (a)~(d) without height correction, (e)~(f) with height correction.

Supplementary Figures 23 (a) and (b) are rescaled versions of Supplementary Figures 22 (b) and (h), respectively. In these figures, Hertz model fitting was applied to specific segments without using x-offset correction, and the fitting was anchored at the contact point. This approach revealed a trend of decreasing Young's modulus after the region where the rate of distance change increases relative to the applied force.

Interestingly, the Young's modulus values obtained from the first fitted segments near the contact point were already significantly higher than the average values observed at lower setpoints (20 and 30 nN). This pattern was consistently observed across multiple measurements with higher setpoints.

From Figures 9 to 24, the force-distance curves showing high apparent moduli generally did not exhibit the typical slope change associated with densification. Instead, the slopes were consistently steep throughout the curves. In contrast, Figure 25 displays force-distance curves with lower moduli that clearly show the characteristic shape of densification. At setpoints of 50, 70, and 100 nN, force-distance curves indicative of penetration or, in rare cases, showing a decrease in slope suggestive of plastic deformation were observed. This plastic deformation–like behaviour, as shown in Supplementary Figure 6–19 (c), is characterized by a decrease in modulus at greater indentation depths compared to the modulus at the initial indentation depth. Although curves with similar penetration depths were identified, it is likely that slip, penetration, plastic deformation, and densification occur simultaneously, making it difficult to clearly distinguish and interpret these effects individually. Additionally, considering Supplementary Figures 23 and 25 together, the force-distance curves exhibit patterns typically associated with penetration and plastic deformation during indentation. Interestingly, however, no pits, marks, or other indications of penetration or plastic deformation are observed in the height maps shown in Supplementary Figure 7 While this could potentially be attributed to a self-healing effect of the molecular crystals, the overall trend of increasing measured modulus with higher loading setpoints suggests that these observations are unlikely to reflect typical vertical penetration or plastic deformation.

Supplementary Figure 24 presents the force–distance curves obtained at a setpoint of 100 nN. Similar to the observations in Supplementary Figures 16 and 20, slight fluctuations were observed in certain slope regions. However, the overall fitting yielded very high $R^2$ values ranging from 0.98 to 0.99. This suggests that, rather than being significantly influenced by densification effects, the curves likely exhibited minor local fluctuations. Therefore, it is cautiously interpreted that these variations are not indicative of densification but rather reflect small deviations within otherwise well-behaved data.

In Supplementary Figure 8 (a), increasing the setpoint from 50 nN to 70 nN resulted in a clear shift in the stiffness histogram toward higher stiffness values. At 100 nN, high stiffness was still observed, although the values were slightly reduced compared to those at 70 nN. This suggests that a certain influencing factor increases the measured stiffness as the setpoint rises, but its effect may begin to diminish at very high forces like 100 nN.

Supplementary Figure 7 (b) further supports this trend, showing a decrease in indentation depth at higher setpoints. While Young's modulus values remained similar at 20 nN and 30 nN, a significant increase in modulus was observed at higher setpoints. Notably, even at 30 nN, the indentation depth had already reached approximately 2 nm, and similar depths were recorded at 50 and 70 nN. This implies that the measurements at low forces were not merely capturing superficial surface effects, but rather reflecting bulk material properties. Therefore, the sudden increase in modulus at higher setpoints is not simply due to a deeper probing of the material.

Interestingly, no clear signs of densification were observed in the force–distance curves associated with high modulus values. This contrasts with the data in Supplementary Figure 25, where only curves with extremely low modulus displayed the characteristic nonlinear slope changes associated with densification. While this observation is intriguing, the primary objective remains to understand the cause behind the generally high moduli seen in most measurements.

These high modulus values observed under high setpoint conditions are likely not representative of intrinsic material properties. Instead, they are suspected to result from external factors or measurement artifacts. One strong possibility is that the measured force is overestimated due to lateral (in-plane) motion of the AFM tip during loading. Such motion could create the illusion of increased stiffness or modulus.

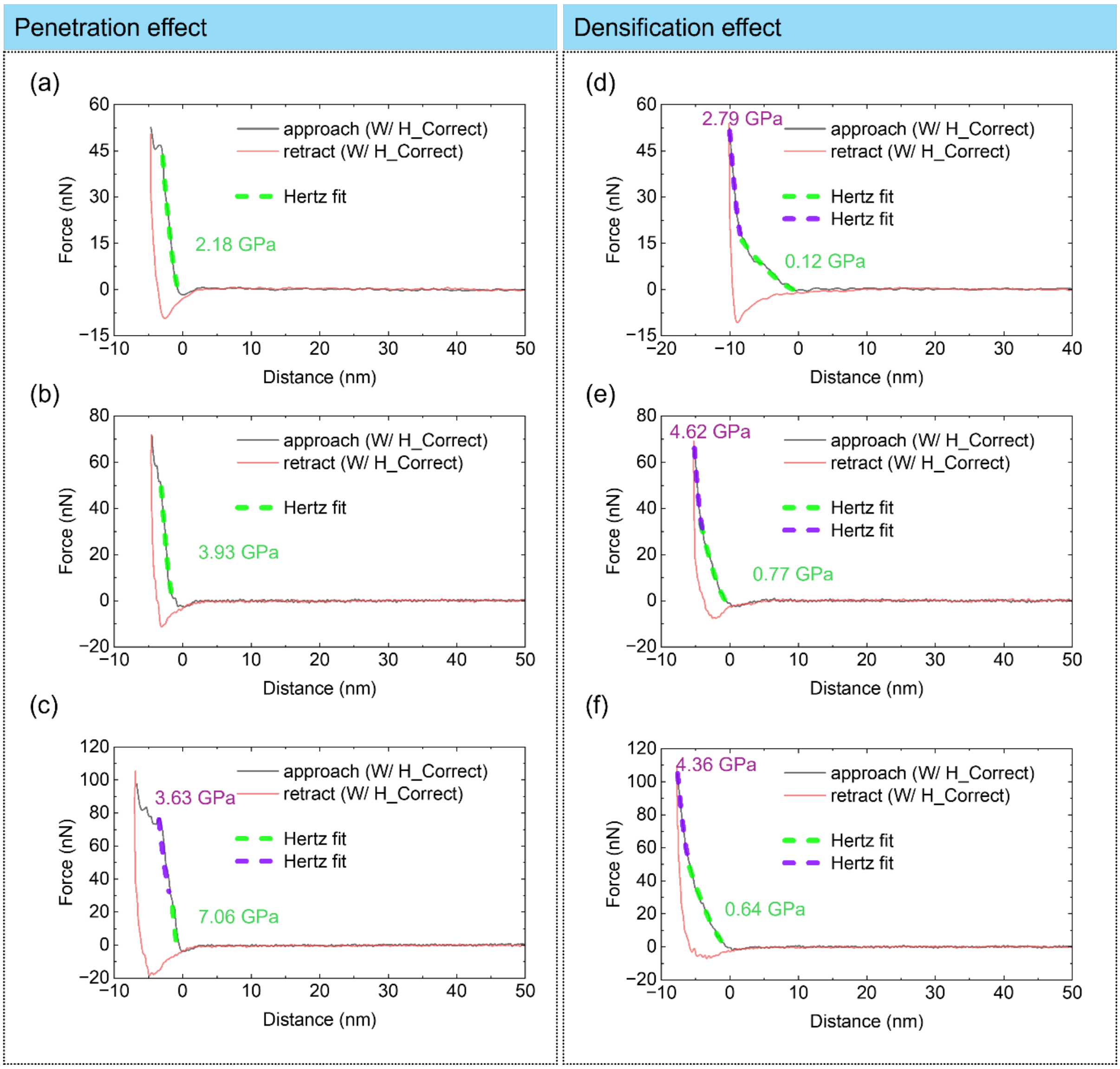


**Supplementary Figure 25** Force-distance curves showing penetration phenomenon and densification effect at 50, 70, and 100 nN setpoints

**II. Lateral shear of AFM tip upon increasing the normal force (i.e., the setpoint)**
Generally, the JPK AFM system has a 10-degree tilted cantilever on the glass block-cantilever holder which may introduce lateral stress to the tip. To assess the factors of lateral stress in relation to the elastic modulus and setpoint, Finite Element Method (FEM) analysis using Ansys software (v2021 R1) was employed. For FEM analysis, as shown in the Supplementary Figure 26 (a), we conducted modelling by analysing the dimensions of the cantilever through SEM image analysis. The Rhino program (v7.0) was used for 3D modelling as shown in Figure 26 (b).

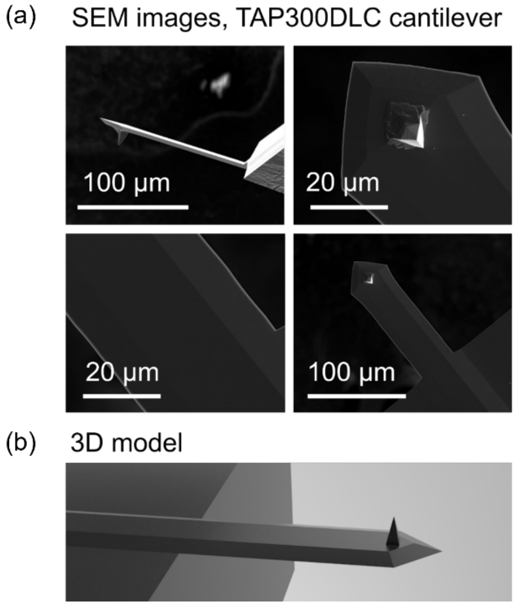


**Supplementary Figure 26** Information on the TAP300DLC AFM tip used, (a) SEM image, and (b) 3D modelling image.

Under ideal conditions, it is assumed that the AFM tip does not move laterally on the sample surface during measurement. In typical tapping mode, this assumption holds because the contact area is minimal and the attractive forces dominate, making lateral movement unlikely. However, this may not be the case during force–distance curve measurements.

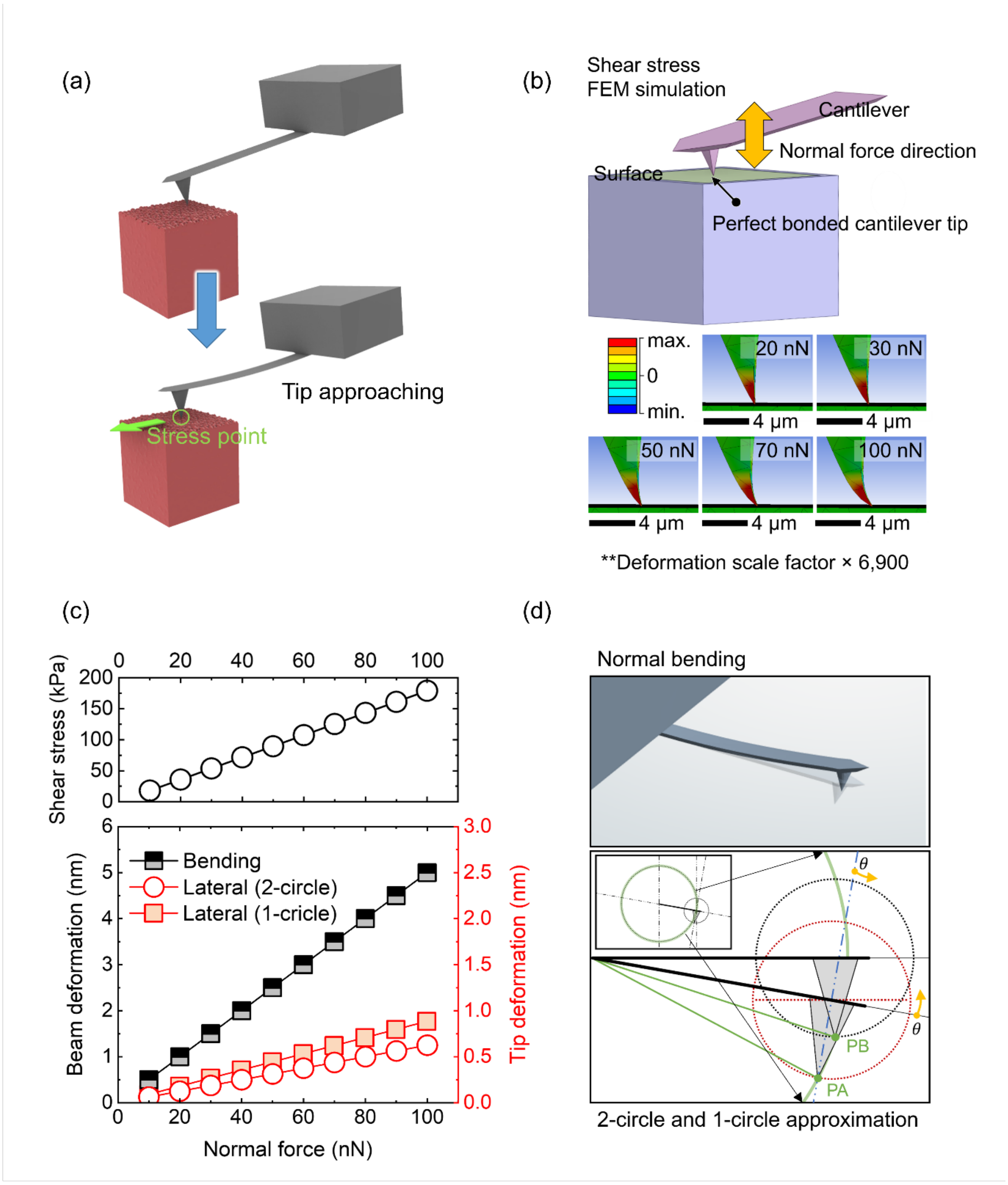


**Supplementary Figure 27** (a) Initial configuration for the FEM simulation; (b) Shear stress distribution and correlation between cantilever bending in the normal force direction and lateral tip movement; (c) Graphs showing shear stress and lateral tip displacement under various normal forces; (d) Model for calculating lateral displacement based on single-circle rotation or a dual rotating-circle mechanism.

As previously mentioned, Supplementary Figure 27 (a) schematically illustrates the generation of lateral (in-plane) stress during the AFM tip's approach and indentation processes. This stress arises from the structural misalignment of the cantilever, which is not perfectly vertical during contact. To investigate the resulting in-plane stress, FEM simulations were performed under the assumption that the AFM tip is fully fixed to the

surface, as shown in Supplementary Figure 27 (b). While the resulting deformation may resemble bending of the tip, in reality, the adhesion force between the tip and the surface is unlikely to be strong enough to induce actual bending. Instead, under certain conditions, slip motion is more likely to occur.

In Supplementary Figure 27 (c), the upper graph shows a linear increase in lateral stress with increasing loading force. The lower graph presents a simple calculation of the lateral movement of the tip, based on the simplified geometric model shown in Supplementary Figure 27 (d). Two approaches were used for this estimation. The first assumes the cantilever undergoes rotation rather than pure bending, with the rotation angle matching that of the tip. Based on this, the lateral displacement of the tip was estimated using the equation: $Cantilever\ movement_{bending} \times \frac{length_{tip\ height}}{length_{cantilever}} = Tip\ movement_{horizontal}$. This approach helps in understanding the relationship between cantilever bending and tip displacement. However, it is important to note that increasing the cantilever length can significantly alter the spring constant, which must be considered in such calculations. The second method uses the circle equation $x^2 + y^2 = r^2$ to estimate the tip's lateral displacement by modeling the rotation at the tip apex, where the radius $r$ is determined by both the tip height and cantilever length.

The key point demonstrated in Supplementary Figure 27 (c) is that even angstrom-scale lateral displacements on the surface can result in significant cantilever bending. Therefore, during force–distance curve measurements, any slight lateral movement of the tip may cause an overestimation of the measured force due to additional cantilever bending.

### III. Hypothesis and discussion on the appearance of the plateau region in force–distance curves at high setpoints despite the absence of plastic deformation in forward force curve measurements with increasing indentation in DNTT.

As shown in Supplementary Figure 27, stress along a specific in-plane direction, arising from the structural characteristics of the AFM system, may be present during the indentation process. In well-aligned liquid crystals such as DNTT, which exhibit orientation along a specific direction, the likelihood of slip or movement along the in-plane direction may be higher. This hypothesis is explored in more detail in this section.

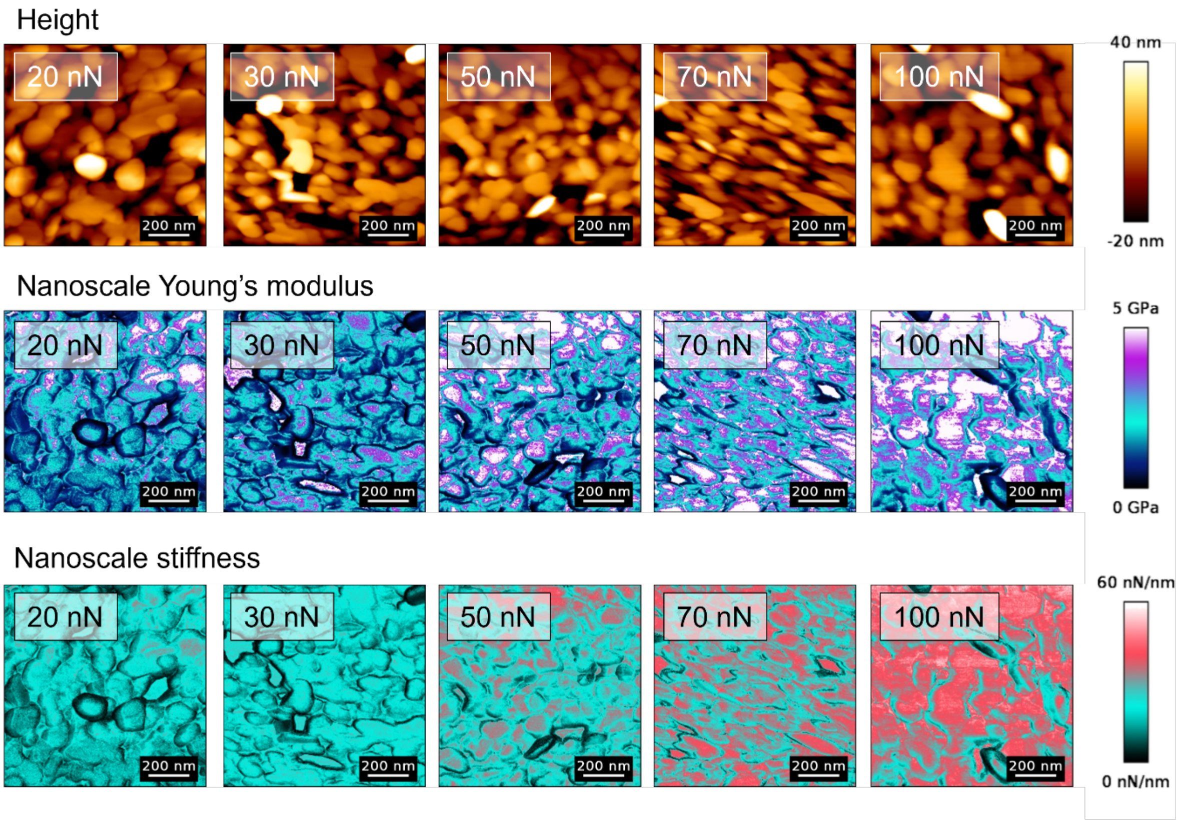


**Supplementary Figure 28** Height, Young's modulus, and nanoscale stiffness maps according to setpoint

As observed in the Young's modulus map of Supplementary Figure 28, regions exhibiting high modulus values are predominantly distributed within the interior of DNTT grains rather than along the grain boundaries, particularly in areas with greater height. This trend is also evident in the stiffness map and becomes more pronounced when a higher setpoint is applied—i.e., when the in-plane stress increases.

Supplementary Figure 29 presents a line profile taken from the height map. In the case of DNTT and its derivatives, it is well known that they form layered structures with lateral intermolecular interactions [11]. Furthermore, XRD analysis confirmed that the unit cells

of DNTT and its derivatives match well with the observed diffraction angles, indicating that the molecules are well-aligned along the z-direction, as illustrated.

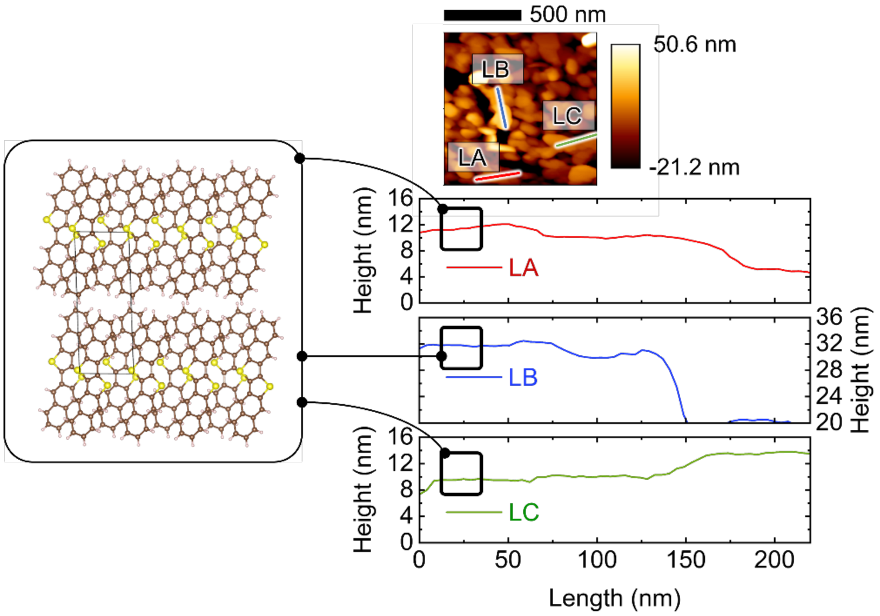


**Supplementary Figure 29** Line profile images at 1 by 1 sq-μm and DNTT crystal structure

The AFM force-distance curve is fundamentally based on a z-directional approach, as illustrated in Supplementary Figure 30 (a). Due to the vertically stacked molecular arrangement of DNTT, it is expected to exhibit anisotropic elastic properties. As shown in Supplementary Figure 30 (b), strong intermolecular interactions such as π–π stacking exist in the xy-plane, resulting in high lateral connectivity. In contrast, the molecular interactions along the z-direction are relatively weak. This directional dependence of bonding, depicted in Supplementary Figure 30 (c), suggests that when lateral or in-plane stress is applied, the weak z-directional interactions may lead to an increased likelihood of slippage during indentation. This scenario is further illustrated in Supplementary Figure 30 (d). In particular, under bending-based measurements, the probability of slippage is expected to be even higher, as such conditions involve not only compressive (z-directional) stress but also lateral and shear stresses. These additional stresses can more easily overcome the weak vertical bonding in organic crystals like DNTT, thereby promoting slip behaviour.

These possibilities are supported by previous findings. As described at the end of Section 5.I, "The influence of setpoint on the measured values of elastic properties", Supplementary Figure 8 (a) shows that increasing the setpoint from 50 to 70 nN shifts the stiffness histogram toward higher values, with a slight decrease at 100 nN. This trend suggests the involvement of external factors that enhance the measured stiffness at higher setpoints, although their influence may decline at very high forces. Supplementary Figure 7 (b) further supports this, demonstrating a decrease in indentation depth with increasing setpoint. While Young's modulus values were similar at 20 and 30 nN, a marked increase was observed at higher setpoints. Importantly, indentation depths of around 2 nm at 30 nN and above indicate that the measurements reflect bulk properties rather than superficial effects.

Additionally, no signs of plastic deformation were observed in high-modulus force–distance curves, nor were any indications of densification—which typically manifests as nonlinear slope changes—found in the curves showing high Young's moduli (around 1.5~2 GPa). In contrast, such densification features were only observed in low-modulus curves, as shown in Supplementary Figure 24.

Supplementary Figure 27 highlights that higher setpoints may induce in-plane stress. Even small lateral displacements of the tip can lead to significant cantilever bending, resulting in an overestimation of the applied force. This aligns with observations in Supplementary Figure 28, where regions with greater height and located within grain boundaries exhibited increased stiffness and modulus, whereas no significant changes were seen in the grain boundary regions themselves. Furthermore, Supplementary Figure 29 and XRD analyses confirm that DNTT forms layered structures with well-aligned molecular orientation, primarily along the z-direction.

Taken together, these findings suggest that at lower setpoints (20~30 nN), in-plane stress is relatively weak and indentation depths are moderate, minimizing measurement artifacts. Consequently, Young's modulus values measured at these setpoints are consistent and likely represent the intrinsic properties of DNTT. This is further supported by the Gaussian distribution of stiffness values under these conditions.

AFM force–distance curves are generated based on z-directional loading, which inherently involves cantilever bending. DNTT, with its stacked layered structure and directional anisotropy, exhibits weak interlayer bonding along the z-direction. When the AFM tip approaches structural discontinuities such as defects or grain boundaries, which disrupt this stacking, z-directional indentation becomes unfavourable. As a result, lateral (in-plane) motion may be induced. If the applied load is sufficient to overcome such structural barriers, z-directional indentation can resume, leading to a sudden transition. This simultaneous interplay between lateral motion and forced z-indentation can appear as a plastic-like deformation in force–distance curves and may also explain the slight drop in Young's modulus after its peak at intermediate setpoints.

This hypothesis is derived from above dataset analysis and is believed to be plausible to a certain extent. Based on this above careful experimental analysis on the setpoints, we would like to propose one possible approach for measuring the elastic properties of DNTT series using AFM. In the typical procedure of measuring nanoscale mechanical properties using AFM, the indentation depth needs to be sufficient to contain information about nano-mechanical properties. Therefore, a certain indentation depth is required, but careful consideration is needed depending on the characteristics of the sample.

It has been reported that for soft organic materials, organic substances can be transferred to the AFM tip edge. So, the second consideration is the situation involving the fine damage to the thin film due to tapping or indentation, along with the potential scenario where material from the sample surface may adhere to it. We measured the elastic modulus at various setpoints using a cantilever that had been used previously for

map scanning with deep indentation. Data from these measurements are shown in Supplementary Figure 31 to 34.

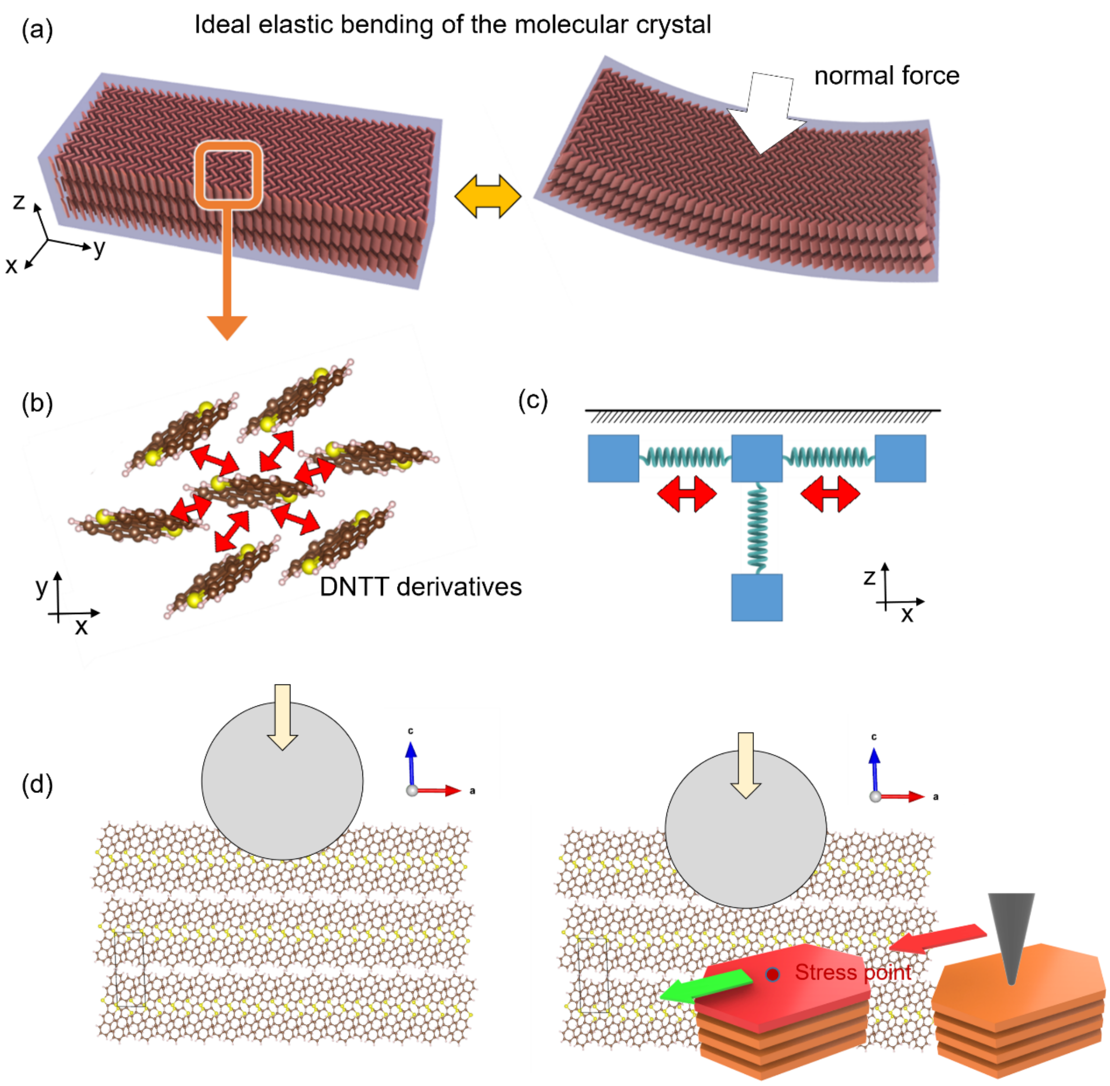


**Supplementary Figure 30** Schematics of a) ideal elastic bending under normal stress; (b) intermolecular interactions in the xy-plane; (c) spring model illustrating anisotropic interactions in the x- and z-directions; (d) indentation near an interlayer boundary in a layered DNTT structure.

## IV. Skewed nanomechanical measurements when using contaminated AFM tips

As shown in Supplementary Figure 31 (a), the overall values of elastic modulus were higher when a contaminated tip was used compared to those obtained using a pristine tip edge surface shown earlier. The inset in Supplementary Figure 31 (a) shows the SEM image of the contaminated tip, which, similar to the 3D model, exhibits an irregular geometry that makes it difficult to accurately determine its radius. The indentation depth showed similar average values at 20, 30, 50, and 70 nN, and significantly increased at 100 nN as shown in Supplementary Figure 31 (b). As shown in Supplementary Figure 31 (c),

the adhesion force was generally higher than when using a clean cantilever tip (See Supplementary Figure 7 for measurements using clean cantilever tips), and it showed an overall increasing trend depending on the setpoint.

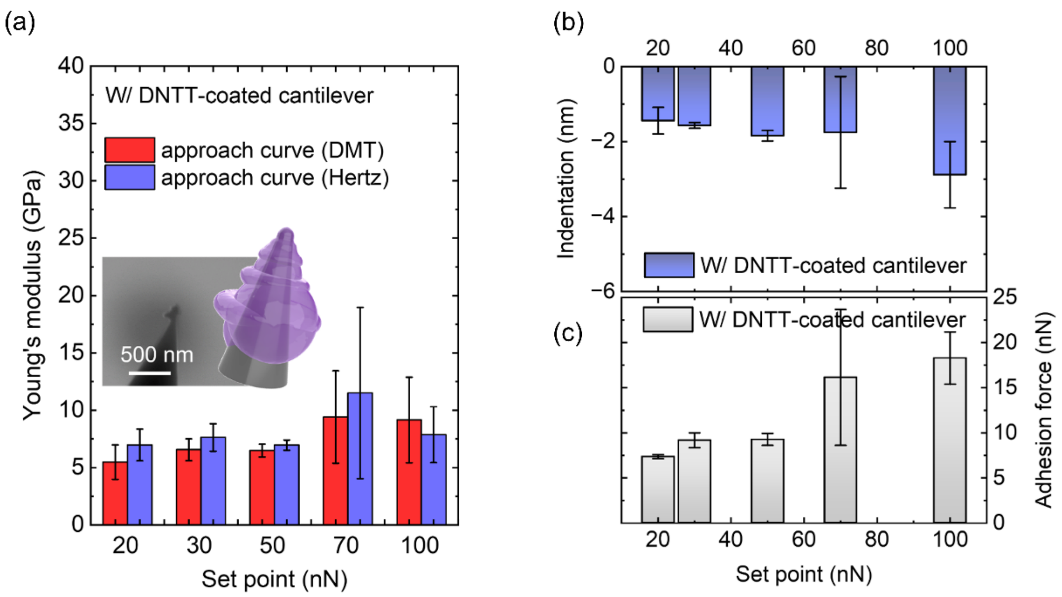


**Supplementary Figure 31** When using a contaminated cantilever tip, (a) correlation between setpoint and elastic modulus, (b) indentation, and (c) adhesion force.

At setpoints of 20, 30, and 100 nN, Supplementary Figure 32 (a) shows the height map, while Supplementary Figure 32 (b) presents the Young's modulus map obtained from the approaching curve. Calculation of Young's modulus from the retraction curve was not performed because large portions of the force-distance curve were unsuitable for Hertzian fitting. Supplementary Figure 32 (c) displays the nanoscale stiffness analysis based on the retraction curve, where a highly unusual phenomenon was observed. This nanoscale stiffness map shows regions where the sign of the stiffness is opposite to the usual pattern. The area highlighted in white in Supplementary Figure 32 (c) represents 'negative stiffness'. This phenomenon is difficult to explain physically, and we would like to suggest that it may be caused by unintended lateral movement of the tip. Essentially, in the data processing stage, a 'height correcting step' needs to be performed to obtain elastic information from the tip bending force and the actual tip-sample separation distance. By applying correction values for bending to the force-distance curve, as shown in Supplementary Figure 33 (a), we can obtain the correct tip-sample height.

If the tip edge is contaminated with a material similar to, or having sufficient adhesion with, the sample being measured, this material may transfer between the tip edge and the sample surface during AFM mapping. [12], [13], [14] This was confirmed by observing changes in the adhesion force during multiple measurements of the force-distance curves, as shown in Supplementary Figure 34 (a). Additionally, in cases where this phenomenon occurred, the presence of a negative slope in the retraction curve was further confirmed through the force-distance curves, as shown in Supplementary Figure 34 (b). As described earlier, when a normal loading force is applied, lateral stress exists.

In this case, if there is material movement, like a 'fluid flow', between surface A (cantilever tip edge) and surface B (sample surface) as shown in Supplementary Figure 33 (b), it can reduce friction, leading to a higher likelihood of lateral movement. If lateral movement increases, incorrect results such as high elastic modulus and negative stiffness may be obtained.

The measurement of negative stiffness is unphysical and is an artifact of using previously used cantilevers. Such measurements of nanoscale stiffness should not be considered in any analysis and reinforces why we changed the cantilever prior to measuring each sample.

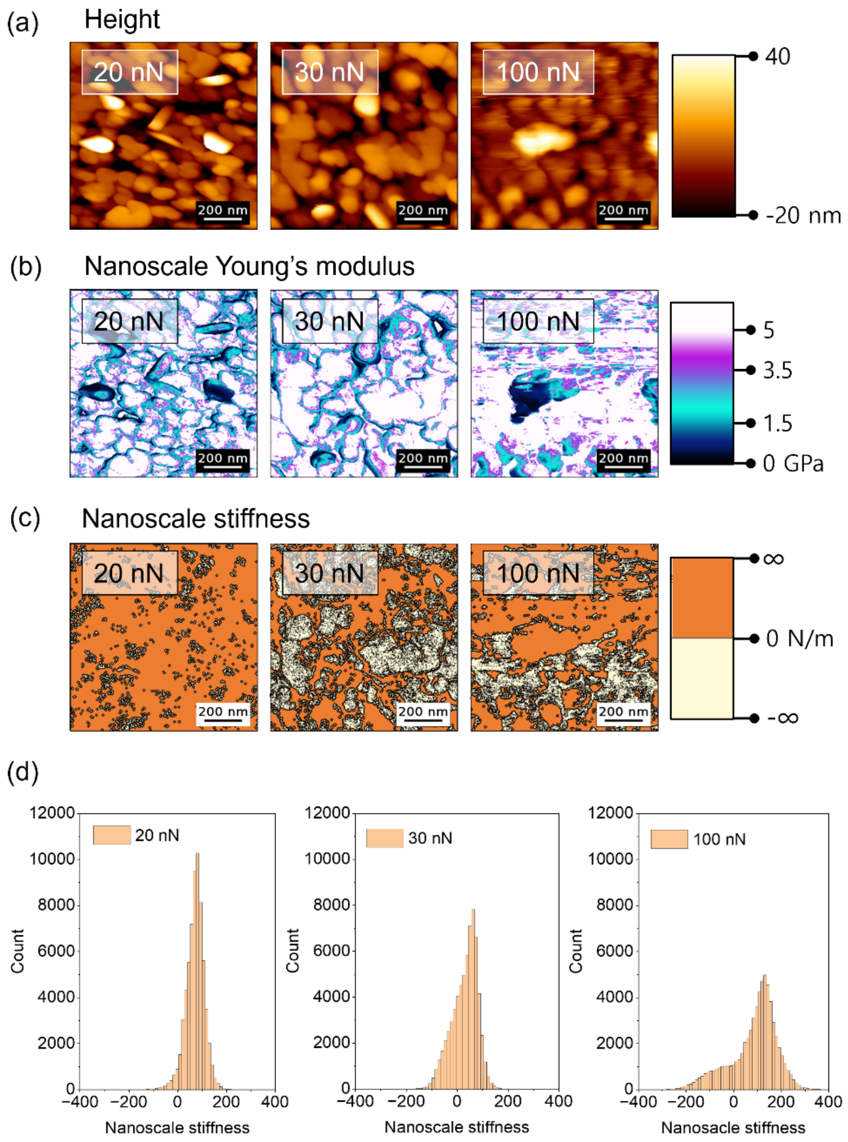


**Supplementary Figure 32** When using a contaminated cantilever tip, (a) height, (b) nanoscale Young's modulus with approaching curve, (c) nanoscale stiffness and (d) its histogram with retract curve.

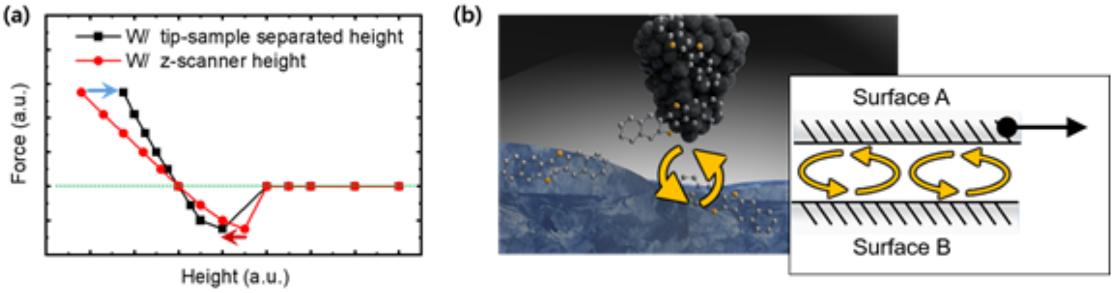


**Supplementary Figure 33** (a) Ideal graph of the height correcting step and (b) Schematic diagram of the flow of deformable material between surfaces A and B.

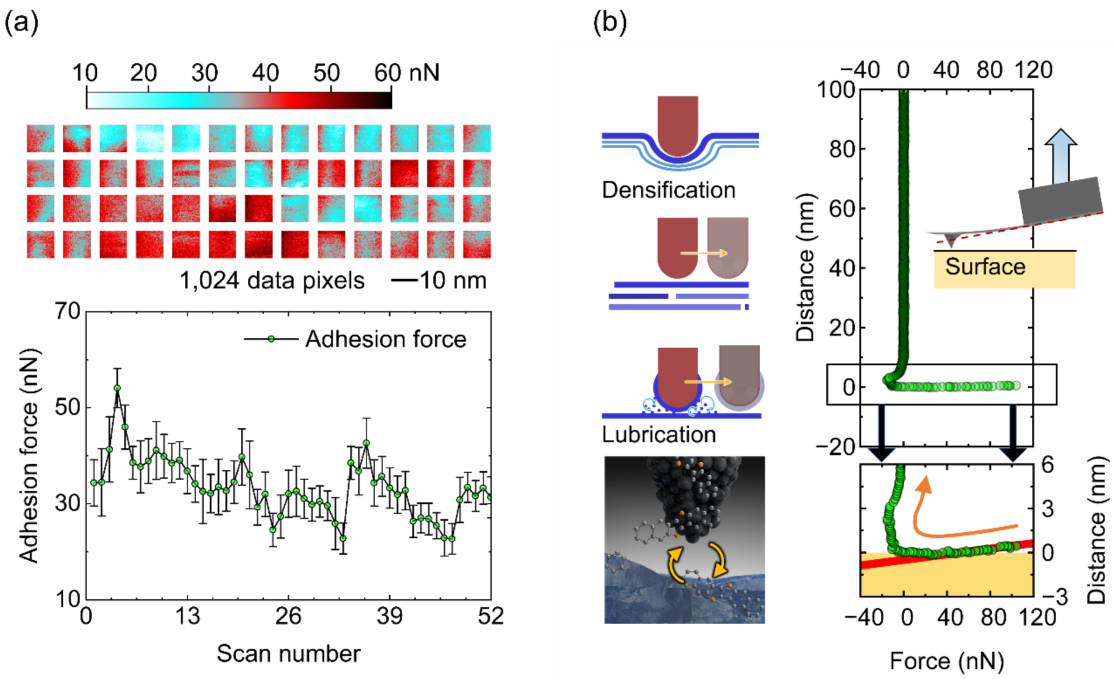


**Supplementary Figure 34** (a) Variation in adhesion force during multiple force–distance measurements, and (b) negative stiffness observed in the retraction curves**.**

Once again, it is emphasized that even a very small lateral movement of the tip can cause the cantilever to experience significant bending in the normal force direction. Therefore, it could be understood that the overall elastic modulus is higher when measured using an unclean surface tip.

Overall, our suggestion is that careful attention be given to the interface between the AFM tip edge and sample surface under investigation to obtain accurate nanomechanical properties. It is important to be vigilant for abnormal changes, including cases where adhesion force varies with increasing setpoint, or where stiffness shows a negative sign during mapping.

**V. Take home message on preforming careful nanomechanical measurements**

In the measurements that we report within the main paper of our work, we have taken care to ensure that the above spurious effects do not contribute. Considering the length of the side chains, our measurements were conducted with indentation depths of 2 nm or more to make sure that we indeed measured the properties of complete molecular layers. All measurements were performed using a new, clean cantilever tip for each scan, and it should be noted that the measurements were carried out under conditions where significant fluctuations in the maps of the nanomechanical properties, such as in the adhesion force, and stiffness, were not present. Spurious fluctuations mean that physical values measured in the mapped image of AFM show a striped pattern when the scanner moves line by line during measurement, or that data values for the same sample appear inconsistently when measured multiple times.

To increase the reliability of the data reported in the main text of the paper, DNTT, Ph-DNTT-Ph, RR-C8*-DNTT-C8*, and SS-C8*-DNTT-C8* were each measured three times at multiple points. Based on all the analysis, the samples of DNTT, Ph-DNTT-Ph, and SS-C8*-DNTT-C8* were measured using a setpoint of 30 nN, C8-DNTT-C8 was measured with a setpoint of 20 nN, and RR-C8*-DNTT-C8* was measured with a setpoint of 10 nN. These values were chosen to compare the measurements of each sample in a sufficiently reliable elastic regime, aiming to prevent unexpected rapid and drastic changes in nanomechanical characteristics that could form lines or stripes representing errors in the data at specific boundaries during measurement mapping. In all cases, an indentation depth of 2 nm or more was observed and was sufficient indentation to be considered as being reliable measurements.

Through this section, we aimed to obtain experimental values from the indentation process in the regime of elastic deformation without errors. Naturally, data that do not pertain to elastic deformation are not suitable as control groups. We used approximately 80 clean cantilevers to find conditions deemed highly reliable, prioritizing reliability over the aesthetic appeal of experimental conditions. In other words, we excluded non-elastic deformation states and aimed to measure the nanoscale mechanical properties of each material by minimizing measurement errors and uncertainties.

Although the content of this SI section does a deep dive into practical experimental methods, we hope that these findings contribute to the advancement of related research measurements in the future. Measurement modes on modern AFMs make it seemingly simple to carry out nanomechanical property estimation with a fast turnaround time, but sufficient care needs to be taken to genuinely understand what these measurements mean. In most cases, if care and diligence are not considered, aesthetic nanomechanical images can be reported in publications which are ridden with artefacts, and which will inadvertently skew the consistency of the literature over time. As ours is one of the first articles to measure nanomechanical properties of organic molecular semiconductors, we have conducted our measurements with at most care, over several years, and using multiple AFMs from JPK to Park Systems, to understand what we are really measuring on the AFM. Our comprehensive work has given us high confidence in the trends that we report within this article on DNTT and its derivatives.

## VI. Python Script Used for Hertz Model Fitting in Force Spectroscopy Analysis

The following Python script was used to perform the force spectroscopy analysis, with a summary of important details provided below.

The relationship between indentation depth and force is inherently nonlinear, and force-spectroscopy analysis was conducted using the Hertz contact model. However, accurately determining the point of contact (i.e., the zero point of indentation) in AFM force data is inherently challenging. An incorrect estimation of this contact point ($x_0$) can significantly degrade the quality of the Hertz model fitting as shown above.

To minimize fitting errors, an automatic offset correction and optimization procedure was implemented. Specifically, a range of possible contact point candidates was tested within $\pm$0.5 nm around the position of minimum force, at intervals of 0.05 nm. For each candidate, the Hertz model was fitted to the data, and the corresponding coefficient of determination ($R^2$) was calculated. The candidate yielding the highest $R^2$ was automatically selected as the optimal x-offset, thereby enabling accurate and robust contact point correction.

The formula for calculating the coefficient of determination is included in the description of Supplementary Figure 11. The Python script for the fitting without using an x offset is available upon request.

```
import numpy as np
import matplotlib.pyplot as plt
from scipy.optimize import curve_fit
from matplotlib.widgets import SpanSelector
import tkinter as tk
from tkinter import filedialog
import pandas as pd

# Input #
radius_nm = float(input("Enter tip radius in nm: "))
poisson_ratio = float(input("Enter Poisson's ratio (0 ~ 0.5): "))
radius_m = radius_nm * 1e-9  # Convert nm to meters

# File Selection #
root = tk.Tk()
root.withdraw()
file_path = filedialog.askopenfilename(title="Select TXT file", filetypes=[("Text files", "*.txt")])

# invert x to make indentation positive #
data = np.loadtxt(file_path)
x_data_raw = data[:, 0]
y_data = data[:, 1]
```

```
x_data = -x_data_raw

# Hertz Model #
def hertz_model(x, E):
    E_eff = E / (1 - poisson_ratio ** 2)
    return (4/3) * E_eff * np.sqrt(radius_m) * x ** 1.5

# Plot Setup #
fig, ax = plt.subplots()
ax.plot(x_data_raw, y_data, label='Raw Data')
ax.set_title("Select fitting range for Hertz model with the mouse")

# Fitting Function #
def onselect(xmin, xmax):
    global df_result, E_result, x0, y0, mask

    mask = (x_data_raw >= xmin) & (x_data_raw <= xmax)
    x_raw = x_data[mask]
    y_raw = y_data[mask]

    if len(x_raw) < 5:
        print("Too few data points selected.")
        return

    # Reference point: minimum y-value #
    min_idx = np.argmin(y_raw)
    x_min_raw = x_raw[min_idx]
    y_min = y_raw[min_idx]

    best_r2 = -np.inf
    best_fit = None

    for delta_nm in np.arange(-0.5, 0.5 + 0.05, 0.05):
        x0_candidate = x_min_raw + delta_nm * 1e-9
        x_fit = x_raw - x0_candidate
        y_fit = y_raw - y_min

        valid = (x_fit >= 0) & (y_fit >= 0)
        x_fit_valid = x_fit[valid]
        y_fit_valid = y_fit[valid]

        if len(x_fit_valid) < 5:
            continue

        try:
            popt, _ = curve_fit(hertz_model, x_fit_valid, y_fit_valid, p0=[1e9])
            y_pred = hertz_model(x_fit_valid, *popt)
```

```
            ss_res = np.sum((y_fit_valid - y_pred) ** 2)
            ss_tot = np.sum((y_fit_valid - np.mean(y_fit_valid)) ** 2)
            r_squared = 1 - (ss_res / ss_tot)

            if r_squared > best_r2:
                best_r2 = r_squared
                best_fit = (x_fit_valid, y_fit_valid, y_pred, popt[0], x0_candidate, valid)

        except RuntimeError:
            continue

    if best_fit is None:
        print("Fitting failed for all attempts.")
        return

    x_fit_valid, y_fit_valid, y_pred, E_result, x0, valid = best_fit
    y0 = y_min

    print(f"\n Young's modulus: {E_result:.3e} Pa")
    print(f" R²: {best_r2:.4f}")

    x_plot_shifted = x_fit_valid + x0
    x_plot_original = -x_plot_shifted
    y_plot = y_pred + y0

    ax.plot(x_plot_original, y_plot, 'r--', label=f'Hertz fit (R²={best_r2:.3f})')
    ax.text(0.05, 0.95, f"E = {E_result:.2e} Pa\nR² = {best_r2:.4f}",
            transform=ax.transAxes, fontsize=10, verticalalignment='top',
            bbox=dict(boxstyle='round', facecolor='white', alpha=0.8))
    ax.legend()
    plt.draw()

    df_result = pd.DataFrame({
        'x_original': x_plot_original,
        'y_fitted': y_plot,
        'x_raw': -x_raw[valid],
        'y_raw': y_raw[valid]
    })

    # Calculate slope of top 30% y-values #
    sorted_indices = np.argsort(-y_fit_valid)
    x_sorted = x_fit_valid[sorted_indices]
    y_sorted = y_fit_valid[sorted_indices]
    n_top30 = int(len(y_sorted) * 0.3)

    if n_top30 >= 2:
```

```
        x_top = x_sorted[:n_top30]
        y_top = y_sorted[:n_top30]
        slope, _ = np.polyfit(x_top, y_top, 1)
        print(f"Slope of top 30% y-values: {slope:.3e}")
    else:
        print("Too few top 30% points to compute slope.")

# Range Selection #
span = SpanSelector(ax, onselect, 'horizontal', useblit=True,
                    props=dict(alpha=0.5, facecolor='red'), interactive=True)
plt.show()
```

**SI Section 6**
**Young's Modulus Calculations**

A. Theory of elasticity

The theory of elasticity for crystals is explained in "Physical properties of crystals: their representation by tensors and matrices" by J. F. Nye. [15] Here, we will provide a quick summary of the concepts and equations.

Hooke's Law is the observation that for sufficiently small stresses, $\sigma$, the amount of strain, $\epsilon$, in a material is proportional to the applied stress:

$$\sigma = c\epsilon.$$

In three dimensions, stresses and strains are represented by second-rank tensors and the general form of Hooke's Law is

$$\sigma_{ij} = c_{ijkl}\epsilon_{kl},$$

where the indices each run from 1 to 3 (*x, y, z*). Notice that instead of a simple spring constant, $c$, we now have a fourth-rank tensor, $c_{ijkl}$, which has 81 components. Luckily, we can reduce the number of independent terms by symmetry arguments. Since $\sigma_{ij}$ and $\epsilon_{ij}$ are symmetric tensors, and since $c_{ijkl}$ is symmetric in the first two and the last two suffixes, we can rewrite $\sigma_{ij} = c_{ijkl}\epsilon_{kl}$ as a matrix equation,

$$\sigma_i = c_{ij}\epsilon_j .$$

The indices now run from 1 to 6 according to the following scheme:

| Tensor | 11 | 22 | 33 | 23, 32 | 31, 13 | 12, 21 |
|---|---|---|---|---|---|---|
| Matrix | 1 | 2 | 3 | 4 | 5 | 6 |

There are also factors of two introduced in the strain tensor (but not in the stress tensor) when changing between vector and tensor notations:

$$[\epsilon_{ij}] = \begin{bmatrix} \epsilon_1 & \frac{1}{2}\epsilon_6 & \frac{1}{2}\epsilon_5 \\ \frac{1}{2}\epsilon_6 & \epsilon_2 & \frac{1}{2}\epsilon_4 \\ \frac{1}{2}\epsilon_5 & \frac{1}{2}\epsilon_4 & \epsilon_3 \end{bmatrix}$$

The elastic compliance tensor $s_{ijkl}$relates the stress and strain by

$$\epsilon_{ij} = s_{ijkl}\sigma_{kl}.$$

In the matrix notation described above, the elastic compliance is just the matrix inverse of the elastic stiffness,

$$[s_{ij}] = [c_{ij}]^{-1}.$$

We reported in Table 1 both the stiffness and compliance matrices:

Table 1: Stiffness and compliance matrices for DNTT and C8-DNTT-C8 and their mechanical properties.

| | | DNTT | C8-DNTT-C8 |
|---|---|---|---|
| ***C*** (GPa) | | $\begin{bmatrix} 13.11 & 8.95 & 6.39 & 0.00 & -1.57 & 0.00 \\ 8.95 & 10.33 & 2.08 & 0.00 & -0.019 & 0.00 \\ 6.39 & 2.08 & 30.53 & 0.00 & 3.95 & 0.00 \\ 0.00 & 0.00 & 0.00 & 0.20 & 0.00 & -0.82 \\ -1.57 & -0.019 & 3.95 & 0.00 & 4.02 & 0.00 \\ 0.00 & 0.00 & 0.00 & -0.82 & 0.00 & 5.99 \end{bmatrix}$ | $\begin{bmatrix} 11.86 & 6.72 & 8.87 & 0.00 & -1.32 & 0.00 \\ 6.72 & 9.96 & 2.52 & 0.00 & -0.58 & 0.00 \\ 8.87 & 2.52 & 25.73 & 0.00 & -6.07 & 0.00 \\ 0.00 & 0.00 & 0.00 & 0.80 & 0.00 & -0.37 \\ -1.32 & -0.58 & -6.07 & 0.00 & 8.86 & 0.00 \\ 0.00 & 0.00 & 0.00 & -0.37 & 0.00 & 3.88 \end{bmatrix}$ |
| ***S*** (GPa$^{-1}$) | | $\begin{bmatrix} 0.30 & -0.25 & -0.07 & 0.00 & 0.18 & 0.00 \\ -0.24 & 0.30 & 0.05 & 0.00 & -0.14 & 0.00 \\ -0.07 & 0.05 & 0.05 & 0.00 & -0.08 & 0.00 \\ 0.00 & 0.00 & 0.00 & -57.60 & 0.00 & -7.87 \\ 0.19 & -0.14 & -0.08 & 0.00 & 0.40 & 0.00 \\ 0.00 & 0.00 & 0.00 & -7.87 & 0.00 & -0.91 \end{bmatrix}$ | $\begin{bmatrix} 0.19 & -0.12 & -0.06 & 0.00 & -0.02 & 0.00 \\ -0.12 & 0.17 & 0.03 & 0.00 & 0.01 & 0.00 \\ -0.06 & 0.03 & 0.07 & 0.00 & 0.04 & 0.00 \\ 0.00 & 0.00 & 0.00 & 1.31 & 0.00 & 0.12 \\ -0.02 & 0.01 & 0.04 & 0.00 & 0.16 & 0.00 \\ 0.00 & 0.00 & 0.00 & 0.12 & 0.00 & 0.27 \end{bmatrix}$ |
| $E$ (GPa) | **x** | 3.3 | 5.2 |
| | **y** | 3.3 | 5.8 |
| | **z** | 18.3 | 14.5 |
| $\upsilon$ | xy | 0.82 | 0.67 |
| | xz | 1.3 | 0.90 |
| | yx | 0.82 | 0.60 |
| | yz | -0.91 | -0.39 |
| | zx | 0.23 | 0.31 |
| | zy | -0.17 | -0.15 |

The reciprocal of Young's Modulus, $E$, in the direction of the unit vector $l_i$ for a triclinic system is given by Nye (pg. 144), [15]

$$\begin{aligned}\frac{1}{E} = {} & l_1^4 s_{11} + 2l_1^2 l_2^2 s_{12} + 2l_1^2 l_3^2 s_{13} + \\ & + \left(2l_1^2 l_2 l_3 s_{14}\right) + 2l_1^3 l_3 s_{15} + \left(2l_1^3 l_2 s_{16}\right) + \\ & + l_2^4 s_{22} + 2l_2^2 l_3^2 s_{23} + \left(2l_2^3 l_3 s_{24}\right) + \\ & + 2l_1 l_2^2 l_3 s_{25} + \left(2l_1 l_2^3 s_{26}\right) + l_3^4 s_{33} + \\ & + \left(2l_2 l_3^3 s_{34}\right) + 2l_1 l_3^3 s_{35} + \left(2l_1 l_2 l_3^2 s_{36}\right) + \\ & + l_2^2 l_3^2 s_{44} + \left(2l_1 l_2 l_3^2 s_{45}\right) + 2l_1 l_2^2 l_3 s_{46} + \\ & + l_1^2 l_3^2 s_{55} + \left(2l_1^2 l_2 l_3 s_{56}\right) + l_1^2 l_2^2 s_{66}\,.\end{aligned}$$

B. Methods

The experimental crystal structures were fully relaxed using the FHI-aims software package, [16], [17], [18], [19] which uses numeric atom-centred orbitals. The calculations employed the B86bPBE density-functional, [20], [21] the XDM dispersion correction, [22], [23] the "light" or "tier1" basis sets, "tight" or "tier2" integration grids (with l_hartree = 4), and the atomic ZORA scalar relativity correction. [16] The XDM damping parameters were set to $a_1$ = 0.6913 and $a_2$ = 1.5747 Å. The numbers of $k$-points were determined according to

$$n_i = \text{int}\left[\max\left(1, R_k\,|b_i| + \frac{1}{2}\right)\right]$$

where $n_i$ is the number of $k$-points in the direction $i$, $|b_i|$ is the length of the $i$th reciprocal lattice vector, and $R_k$ was chosen to be 50.0 Bohr.

After the structures were optimized, we generated a series of strained unit cells for each structure by transforming the optimized lattice vectors according to the prescription in Ref. [24], which provides six "universal linearly independent coupling strains" that are designed to probe the entire elastic constant matrix, $c_{ij}$. The six strain vectors are given in the accompanying Table, which is reproduced from Ref. [24]. These strains are chosen because they span the entire strain space and, since there are no zero components, they ensure that all stress components and elastic constants are coupled.

Table 8: The six "universal linear-independent coupling strains" given in Ref. [24].

| | $\epsilon_1$ | $\epsilon_2$ | $\epsilon_3$ | $\epsilon_4$ | $\epsilon_5$ | $\epsilon_6$ |
|---|---|---|---|---|---|---|
| $U_1$ | 1 | 2 | 3 | 4 | 5 | 6 |
| $U_2$ | -2 | 1 | 4 | -3 | 6 | -5 |
| $U_3$ | 3 | -5 | -1 | 6 | 2 | -4 |
| $U_4$ | -4 | -6 | 5 | 1 | -3 | 2 |
| $U_5$ | 5 | 4 | 6 | -2 | -1 | -3 |
| $U_6$ | -6 | 3 | -2 | 5 | -4 | 1 |

Because of the monoclinic symmetry of DNTT and C8-DNTT, only the first five of these strains are needed to calculate all elements of the elastic stiffness tensor. For each of the first five vectors given in the Table, we generated eight different magnitudes of the strain by multiplying the vector by eight numbers ranging from -0.003 to 0.003. The equilibrium lattice vectors, $\boldsymbol{r}_{eq}$, are then transformed according to

$$\boldsymbol{r}_i = \boldsymbol{r}_{eq,i} + \epsilon_{ij}\boldsymbol{r}_{eq,j},$$

where the strain tensor, $\epsilon_{ij}$, is generated by one of the (5 × 8 =) 40 strain vectors.

For each of the distorted unit cells, we placed the atoms in their initial fractional coordinates and performed another geometry optimization using the same computational methods described above, but with the cell vectors fixed. The "compute_analytical_stress .true." option in FHI-aims was used to calculate the stress on the cell. [19]

With all of the stress calculations done for the various strains, we performed a linear fit to find the components of the elastic constant matrix, $c_{ij}$, which minimize the error in the equation $\sigma_i = c_{ij}\epsilon_j$. We can then calculate the Young's modulus for any direction by application of the computed $s_{ij}$ and the reciprocal of the Young's Modulus, $1/E$, shown earlier. Inparticular, we calculated the value of Young's modulus along the $(001)$ direction to mimic the experiments as best as possible.

**SI Section 7**
**Mechanical Properties determination employing Molecular Dynamics (MD) simulations**

## Modeling strategy

All the MD simulations were performed using the Nanoscale Molecular Dynamics (NAMD) package [25] with a Force Field (FF) derived from the generalized amber force field (GAFF) [26] where the torsional and the van der Waals parameters were tuned according to the literature [27]. The atomic charges were also taken from the literature [27].
The initial coordinates of DNTT and C8-DNTT-C8 extracted from their crystal structure [28] [29] were replicated four times in the x and y directions and two times in the z-direction, and the lattice parameters were increased accordingly. Using periodic boundary conditions (PBCs), DNTT and C8-DNTT-C8 supercells were replicated in the three dimensions to create an infinite system, and the electrostatic energy of the periodic system was calculated using the Particle Mesh Ewald (PME) method [30]. The mechanical strain was investigated for DNTT by applying uniaxial strain with the following protocol : (i) we performed a constant pressure simulation (NPT, T = 298 K and P = 1 atm) of the unstrained system, allowing for anisotropic relaxation of the lattice parameters, until the volume of the system was stable at the target atmospheric pressure, (ii) during the successive 10 ns NVT simulation (T = 298 K), we evaluated the pressure tensor $\boldsymbol{P}$ as a function of time and we averaged it over simulation time, (iii) we applied a uniaxial strain ($\varepsilon$, up to ±1.0% with 0.5 % interval) by accordingly scaling the x, y and z axes and the intermolecular distances and we calculated the pressure tensor $\boldsymbol{P}'$ by averaging it on a 10 ns NVT simulation at 298 K similar to (ii), (iv) from the stress tensor ($\boldsymbol{\sigma} = \boldsymbol{P}' - \boldsymbol{P}$) it was derived the stiffness tensor $\boldsymbol{C}$ in Voigt notation following Hooke's law [31]. The same approach was used for C8-DNTT-C8, with doubled simulation times. Indeed, the increase in simulation time is required to improve the configurational sampling of the alkyl chains.

## Mechanical properties

This section provides further details on how uniaxial deformation is applied to the two systems, as mentioned at the end of point (ii) of the modeling strategy paragraph of the main paper. The crystalline systems were modified by changing the size of the supercell and the intermolecular distances along the direction where the strain is applied (either along the x-, y- or z-axis) so that the dimensions of the cell and the intermolecular distances in the other two cartesian directions remained unaltered. These deformations were made with increments of 0.5% to obtain deformations in the range of -1.0% to 1.0% in the case of DNTT. For C8-DNTT-C8, the deformations were performed with increments of 0.5% for contractions and with increments of 0.25% for elongations, thus providing a final range from -1.00% to 0.50%. This choice was made because a change in the crystal organization of the C8-DNTT-C8 supercell was observed when the strain increment was 0.50%. Indeed, by expanding the C8-DNTT-C8 supercell by a 0.50% strain increment, we observe a tilt of the molecules with respect to the z-axis so that they are no longer aligned with the z-axis of the supercell. In this case, we might have accessed a C8-DNTT-C8 crystalline polymorph only accessible upon mechanical constrain.
The normal stresses are extracted from the 4 simulations carried out in the case of the two systems, giving the stress $\sigma$ relative to each uniaxial strain $\varepsilon$ (Supplementary Figures 35 and 36).

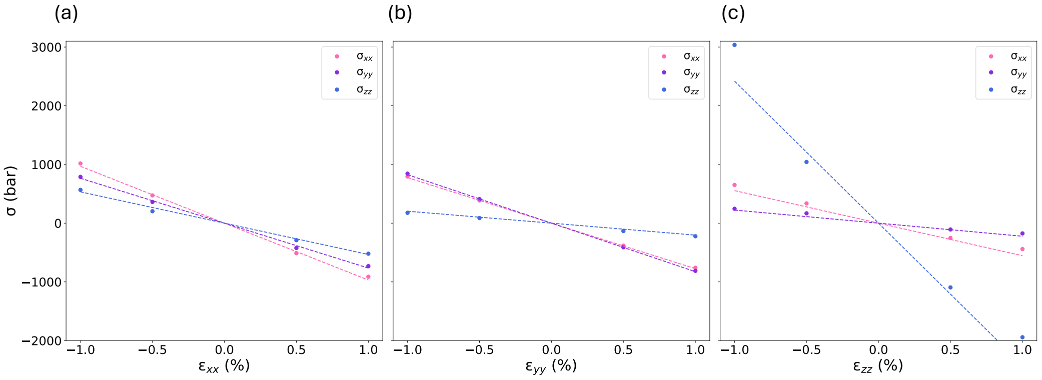

**Supplementary Figure 35: DNTT stress-strain curves.** (a) Normal stresses for uniaxial deformations along x, (b) along x, and (c) along z.

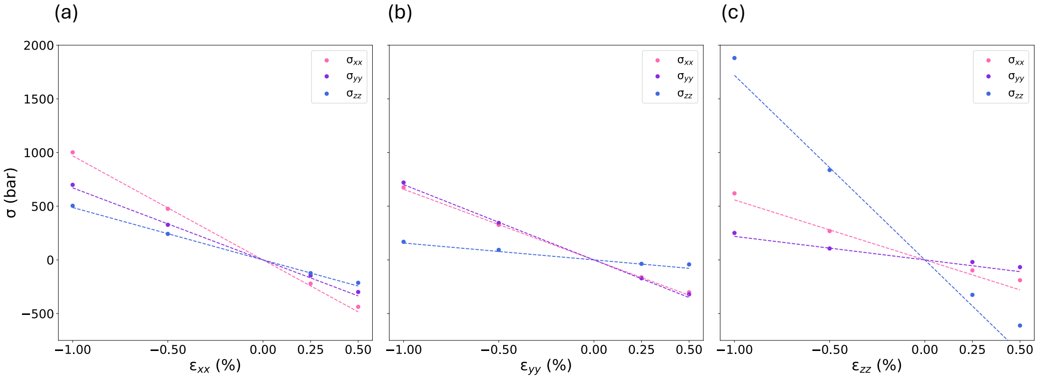

**Supplementary Figure 36: C8-DNTT-C8 stress-strain curves.** (a) Normal stresses for uniaxial deformations along x, (b) along y, and (c) along z.

Using Hooke's law, the mathematical dependence between stress σ and strain ε is given by the stiffness tensor $\boldsymbol{C}$, as according to the equation:

$$\sigma_{ij} = \sum_{i}\sum_{k} C_{ijkl}\,\varepsilon_{kl} \qquad \text{(SE1)}$$

where $i, j, k$ and $l$ can assume values from 1 to 3 and they correspond to reference axes x, y and z, respectively. Given that we only choose to consider normal stresses (so that shear stresses are neglected) and normal strains, and by using the commonly employed Voigt's notation, Hooke's law is simplified as follows:

$$\sigma_i = \sum_{k} C_{ik} \cdot \varepsilon_k \qquad \text{(SE2)}$$

where the stiffness tensor contains only six different elements being a symmetric 3 x 3 matrix (*i.e.* $C_{ik} = C_{ki}$).

$$\begin{pmatrix}\sigma_X\\ \sigma_Y\\ \sigma_Z\end{pmatrix} = \begin{pmatrix}C_{XX} & C_{XY} & C_{XZ}\\ C_{XY} & C_{YY} & C_{YZ}\\ C_{XZ} & C_{YZ} & C_{ZZ}\end{pmatrix}\begin{pmatrix}\varepsilon_X\\ \varepsilon_Y\\ \varepsilon_Z\end{pmatrix} \qquad \text{(SE3)}$$

Since in our simulations we applied only uniaxial strains, we obtain three different strain-stress equations, one for each axis of deformation:

$$\begin{pmatrix}\sigma_X\\ \sigma_Y\\ \sigma_Z\end{pmatrix} = \boldsymbol{C}\begin{pmatrix}\varepsilon_X\\ 0\\ 0\end{pmatrix} \quad \text{(SE4)}$$

$$\begin{pmatrix}\sigma_X\\ \sigma_Y\\ \sigma_Z\end{pmatrix} = \boldsymbol{C}\begin{pmatrix}0\\ \varepsilon_Y\\ 0\end{pmatrix} \quad \text{(SE5)}$$

$$\begin{pmatrix}\sigma_X\\ \sigma_Y\\ \sigma_Z\end{pmatrix} = \boldsymbol{C}\begin{pmatrix}0\\ 0\\ \varepsilon_Z\end{pmatrix} \quad \text{(SE6)}$$

It is then possible to obtain the stiffness tensor ($\boldsymbol{C}$) by employing a total least squared algorithm that was iteratively ran to minimize the function $\chi^2$:

$$\chi^2 = \sum_{i=1}(\boldsymbol{\sigma}^2_{SIM\ i} - \boldsymbol{\sigma}^2_{FIT\ i}) \quad \text{(SE7)}$$

where in $\boldsymbol{\sigma}^2_{SIM\ i}$ were stored the averaged values of the components of the stress from simulations, and $\boldsymbol{\sigma}_{FIT} = \boldsymbol{C}_{FIT} \cdot \boldsymbol{\varepsilon}_{SIM}$ with $\boldsymbol{\varepsilon}_{SIM}$ being the uniaxial deformations applied during the simulation.

From the stiffness tensor, the compliance tensor $\boldsymbol{S}$ can be obtained by inverting the stiffness tensor ($\boldsymbol{S} = \boldsymbol{C}^{-1}$). From the compliance tensor, the Young's modulus $E$ and the Poisson's ratio $\upsilon$ can be extracted as follows:

$$\boldsymbol{C}^{-1} = \boldsymbol{S} = \begin{pmatrix}\frac{1}{E_{XX}} & -\frac{\upsilon_{XY}}{E_{YY}} & -\frac{\upsilon_{XZ}}{E_{ZZ}}\\ -\frac{\upsilon_{YX}}{E_{XX}} & \frac{1}{E_{YY}} & -\frac{\upsilon_{YZ}}{E_{ZZ}}\\ -\frac{\upsilon_{ZX}}{E_{XX}} & -\frac{\upsilon_{ZY}}{E_{YY}} & \frac{1}{E_{ZZ}}\end{pmatrix} \quad \text{(SE8)}$$

From the compliance tensor and the stiffness tensor, Bulk modulus $B$ using, respectively, Voigt and Reuss relations [32] can be calculated:

$$9B_{Voigt} = (C_{XX} + C_{YY} + C_{ZZ}) + 2(C_{XY} + C_{XZ} + C_{YZ}) \quad \text{(SE9)}$$

$$B_{Reuss}{}^{-1} = (S_{XX} + S_{YY} + S_{ZZ}) + 2(S_{XY} + S_{XZ} + S_{YZ}) \quad \text{(SE10)}$$

The values of the stiffness and the compliance tensor components, as well as Young's moduli and the Poisson's ratios for DNTT and C8-DNTT-C8 are reported in Supplementary Table 8.

**Supplementary Table 8: Stiffness and compliance tensors for DNTT and C8-DNTT-C8 and their mechanical properties.**

| | DNTT | C8-DNTT-C8 |
|---|---|---|
| $\boldsymbol{C}$ (GPa) | $\begin{bmatrix}11.2 & 8.68 & 5.97\\ 8.68 & 9.10 & 1.35\\ 5.97 & 1.35 & 29.3\end{bmatrix}$ | $\begin{bmatrix}9.49 & 6.57 & 6.11\\ 6.57 & 7.06 & 2.32\\ 6.11 & 2.32 & 18.5\end{bmatrix}$ |

| | | | |
|---|---|---|---|
| $\boldsymbol{S}$ (GPa$^{-1}$) | | $\begin{bmatrix} 0.454 & -0.422 & -0.0730 \\ -0.422 & 0.503 & 0.0628 \\ -0.0730 & 0.0628 & 0.0461 \end{bmatrix}$ | $\begin{bmatrix} 0.402 & -0.344 & -0.0893 \\ -0.344 & 0.443 & 0.0582 \\ -0.0893 & 0.0582 & 0.0749 \end{bmatrix}$ |
| $E$ (GPa) | $E_{xx}$ | 2.20 | 2.49 |
| | $E_{yy}$ | 1.99 | 2.26 |
| | $E_{zz}$ | 21.7 | 13.1 |
| $\upsilon$ | $\upsilon_{xy}$ | 0.839 | 0.777 |
| | $\upsilon_{xz}$ | 1.58 | 1.17 |
| | $\upsilon_{yx}$ | 0.930 | 0.858 |
| | $\upsilon_{yz}$ | $-1.36$ | $-0.765$ |
| | $\upsilon_{zx}$ | 0.161 | 0.222 |
| | $\upsilon_{zy}$ | $-0.125$ | $-0.131$ |

## FF validation

To validate DNTT's FF, MD simulations using NAMD with a tuned GAFF force field, as described in the Modeling Strategy section, were carried out for a supercell made of 62 DNTT molecules (see Supplementary Figure 38 for the atom type attribution and Supplementary Tables 10, 11, 12, and 13 for the FF parameters). First, the supercell was equilibrated in the *NPT* ensemble at T = 298 K and P = 1 atm for 5 ns. Then, the system was relaxed for 10 ns in the *NVT* ensemble at the same temperature. The values of the cell axes and the density extracted from the latter simulation are reported in Supplementary Table 9. From this equilibrated *NVT* trajectory, we additionally calculated the distances within different molecules of the crystals to verify that the herringbone structure of the crystal was kept intact, as shown in Supplementary Figure 37.

**Supplementary Table 9: Comparison between experimental and MD density and unit cell axes. MD results are obtained from a 5 ns equilibrated NVT trajectory of the DNTT supercell.** $\Delta$V/V and $\Delta\rho/\rho$ correspond to the deviation of the simulated values from the experiments for the cell volume (V) and the density ($\rho$).

| | Experimental (T = 293 K) | Simulation (T = 298 K) |
|---|---|---|
| **a (Å)** | 24.75 | 25.12 |
| **b (Å)** | 30.65 | 31.10 |
| **c (Å)** | 32.42 | 32.90 |
| **$\alpha$ (°)** | 90.00 | 90.00 |
| **$\beta$ (°)** | 92.49 | 92.49 |
| **$\gamma$ (°)** | 90.00 | 90.00 |
| **V (Å$^3$)** | 24566.6 | 25679.0 |
| **$\Delta$V/V** | --- | 0.045 |
| **$\rho$(g/cm$^3$)** | 1.473 [33] | 1.409 |
| **$\Delta\rho/\rho$** | --- | -0.043 |

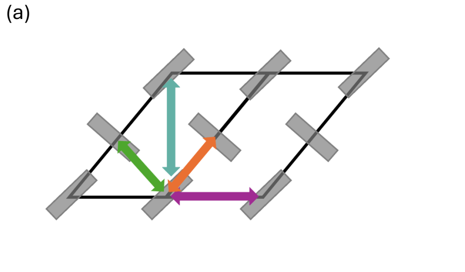


(d)

| | $T_2$ (Å) | $P_b$ (Å) | $T_1$ (Å) | $P_a$ (Å) |
|---|---|---|---|---|
| Experimental (293 K) | 5.184 | 7.662 | 4.886 | 6.187 |
| Simulation (298 K) | 5.28 | 7.79 | 5.02 | 6.28 |
| *Δ*dist./dist. | 0.018 | 0.017 | 0.027 | 0.015 |

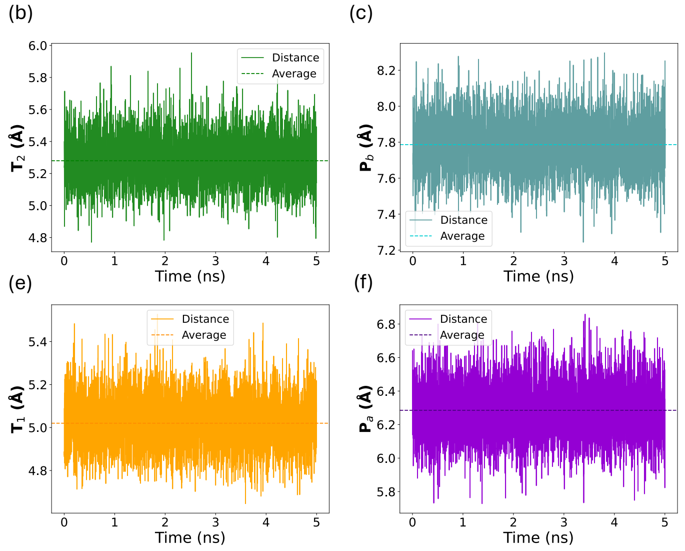


**Supplementary Figure 37: Characteristic distances between DNTT molecules in a herringbone pattern.** (a) Herringbone pattern distances $P_a$, $P_b$, $T_1$, and $T_2$ are highlighted by the purple, blue, orange, and green arrows. (b), (c), (e), and (f) figures correspond to the $T_1$, $P_b$, $T_2$, and $P_a$ distance fluctuations during the last equilibrated 5 ns of the MD simulation. The Table reported in panel (d) reports on the comparison between the distances found in the experimental crystalline structure and the average distances calculated from our simulations. The last row reports the deviation of the simulated characteristic distances with respect to the experiments.

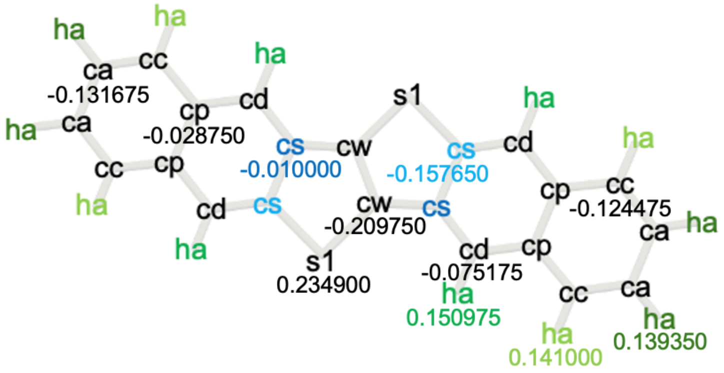


**Supplementary Figure 38: GAFF force field atom types defined for DNTT and their charge computed by the localized bond-charge corrected CM1A (1.14*CM1A-LBCC) charge model [27].**

**Supplementary Table 10: Bond parameters of DNTT**

| | $k_r$ (kcal/mol/Å$^2$) | $r_{eq}$ (Å) |
|---|---|---|
| cw cw | 504.0 | 1.371 |
| cs cs | 418.3 | 1.429 |
| cs cw | 418.3 | 1.429 |
| s1 cw | 277.9 | 1.739 |
| s1 cs | 277.9 | 1.739 |
| cs cd | 504.0 | 1.371 |
| cd cp | 449.9 | 1.406 |
| cd ha | 344.3 | 1.087 |
| cp cp | 418.3 | 1.429 |
| cp cc | 411.7 | 1.434 |
| cc ca | 478.4 | 1.387 |
| ca ca | 478.4 | 1.387 |
| cc ha | 344.3 | 1.087 |
| ca ha | 344.3 | 1.087 |

**Supplementary Table 11: Angle parameters of DNTT**

| | $k_\theta$ (kcal/mol/rad$^2$) | $\theta_{eq}$ (degrees) |
|---|---|---|
| cw s1 cs | 41.420 | 90.360 |
| s1 cw cs | 77.520 | 129.370 |
| s1 cs cs | 80.870 | 112.750 |
| s1 cw cw | 80.870 | 112.750 |
| s1 cs cd | 79.470 | 122.860 |
| cs cs cw | 67.660 | 111.040 |
| cw cs cs | 68.230 | 113.510 |
| cs cd ha | 49.070 | 120.760 |
| cs cd cp | 67.180 | 119.970 |
| cd cp cp | 67.180 | 119.970 |
| cd cp cc | 64.880 | 120.660 |
| cp cp cc | 67.180 | 119.970 |
| cp cc ha | 48.460 | 120.010 |
| cp cc ca | 64.880 | 120.660 |
| cc ca ca | 65.990 | 120.100 |
| cc ca ha | 48.460 | 120.010 |
| ca ca ha | 48.460 | 120.010 |
| ca cc ha | 48.460 | 120.010 |
| cd cs cs | 65.990 | 120.100 |
| cd cs cw | 77.520 | 129.370 |
| cp cd ha | 48.460 | 120.010 |

**Supplementary Table 12: Dihedral parameters of DNTT**

| | $k_\phi$ (kcal/mol) | multiplicity | $\phi$ (degrees) |
|---|---|---|---|
| cs cs cw cw | 2.170 | 2 | 180.0 |
| cs s1 cw cw | 2.170 | 2 | 180.0 |
| cs s1 cw cs | 2.170 | 2 | 180.0 |
| cp cd cs cw | 2.170 | 2 | 180.0 |
| ha cd cs cw | 7.250 | 2 | 180.0 |
| cd cs cs cw | 7.250 | 2 | 180.0 |
| cd cs cw cw | 2.170 | 2 | 180.0 |

| | | | |
|---|---|---|---|
| s1 cs cs cw | 7.250 | 2 | 180.0 |
| cp cd cs cs | 7.000 | 2 | 180.0 |
| ha cd cs cs | 7.250 | 2 | 180.0 |
| cs cs s1 cw | 7.250 | 2 | 180.0 |
| cp cp cd cs | 7.000 | 2 | 180.0 |
| cc cp cd cs | 7.000 | 2 | 180.0 |
| cs cw cw cs | 2.170 | 2 | 180.0 |
| cs cw cw s1 | 2.170 | 2 | 180.0 |
| cd cs cs cd | 7.250 | 2 | 180.0 |
| cd cs cs s1 | 7.250 | 2 | 180.0 |
| ca cc cp cd | 7.000 | 2 | 180.0 |
| ha cc cp cd | 7.250 | 2 | 180.0 |
| ha cd cp cc | 7.250 | 2 | 180.0 |
| cd cs s1 cw | 7.250 | 2 | 180.0 |
| cc cp cp cc | 7.250 | 2 | 180.0 |
| cc cp cp cd | 7.000 | 2 | 180.0 |
| cd cp cp cd | 7.250 | 2 | 180.0 |
| ca ca cc cp | 7.250 | 2 | 180.0 |
| ha ca cc cp | 7.250 | 2 | 180.0 |
| ca cc cp cp | 7.000 | 2 | 180.0 |
| ha cc cp cp | 7.250 | 2 | 180.0 |
| ha cd cp cp | 7.250 | 2 | 180.0 |
| cc ca ca cc | 7.250 | 2 | 180.0 |
| ha ca ca cc | 7.250 | 2 | 180.0 |
| ha cc ca ca | 7.250 | 2 | 180.0 |
| s1 cs cd cp | 7.000 | 2 | 180.0 |
| s1 cs cd ha | 7.250 | 2 | 180.0 |
| s1 cw cw s1 | 7.250 | 2 | 180.0 |
| s1 cw cs cd | 2.170 | 2 | 180.0 |
| s1 cw cs cs | 2.170 | 2 | 180.0 |
| ha ca ca ha | 7.250 | 2 | 180.0 |
| ha ca cc ha | 7.250 | 2 | 180.0 |

**Supplementary Table 13: Non-bonded parameters of DNTT**

| | $\varepsilon$ (kcal/mol) | r (Å) | $\varepsilon_{1-4}$ (kcal/mol) | $r_{1-4}$ (Å) |
|---|---|---|---|---|
| ha | 0.030000 | 1.45900 | 0.015000 | 1.45900 |
| ca | 0.070000 | 1.90800 | 0.035000 | 1.90800 |
| cc | 0.070000 | 1.90800 | 0.035000 | 1.90800 |
| cd | 0.070000 | 1.90800 | 0.035000 | 1.90800 |
| cs | 0.070000 | 1.90800 | 0.035000 | 1.90800 |
| cw | 0.080000 | 1.90800 | 0.040000 | 1.90800 |
| cp | 0.080000 | 1.90800 | 0.040000 | 1.90800 |
| s1 | 0.355000 | 2.00000 | 0.177500 | 2.00000 |

To validate C8-DNTT-C8 FF, MD simulations using NAMD, with a tuned GAFF force field, as described in the Modeling Strategy section, were carried out for a supercell made of 62 DNTT molecules (see Supplementary Figure 40 for the atom types attribution and Supplementary

Tables 15, 16, 17, and 18 for the FF parameters). First, the supercell was equilibrated in the *NPT* ensemble at 298 K for 10 ns. Then, the system was relaxed for 20 ns in the *NVT* ensemble at the same temperature. The values of the cell axes and the density extracted from the latter simulation are extracted and reported in Supplementary Table 14. From this equilibrated *NVT* trajectory, we additionally calculated the distances within the center of mass of different molecules to verify that the herringbone structure of the crystal was kept intact, as shown in Supplementary Figure 39.

**Supplementary Table 14: Comparison between experimental and MD density and unit cell axes. MD results are obtained from a 5 ns equilibrated NVT trajectory of the C8-DNTT-C8 supercell.** $\Delta V/V$ and $\Delta\rho/\rho$ correspond to the deviation of the simulated values from the experiments for the cell volume (V) and the density ($\rho$).

| | Experimental (T = 293 K) | Simulation (T = 298 K) |
|---|---|---|
| **a (Å)** | 23.95 | 24.24 |
| **b (Å)** | 31.44 | 31.82 |
| **c (Å)** | 68.13 | 68.95 |
| **$\alpha$ (°)** | 90.00 | 90.00 |
| **$\beta$ (°)** | 99.86 | 99.86 |
| **$\gamma$ (°)** | 90.00 | 90.00 |
| **V (Å$^3$)** | 50550.7 | 52390.4 |
| **$\Delta V/V$** | --- | 0.036 |
| **$\rho$ (g/cm$^3$)** | 1.123 [34] | 1.103 |
| **$\Delta\rho/\rho$** | --- | -0.018 |

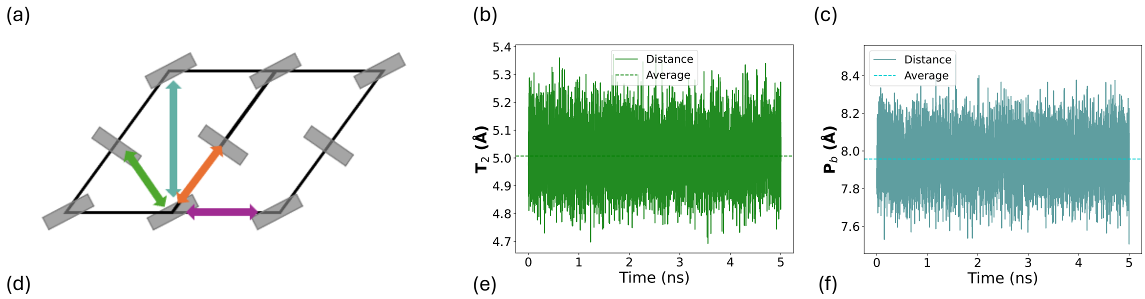


(d)

| | $T_2$ (Å) | $P_b$ (Å) | $T_1$ (Å) | $P_a$ (Å) |
|---|---|---|---|---|
| Experimental (293 K) | 4.941 | 7.861 | 4.941 | 5.988 |
| Simulation (298 K) | 5.01 | 7.96 | 5.01 | 6.06 |
| *Δ*dist./dist. | 0.014 | 0.012 | 0.014 | 0.012 |

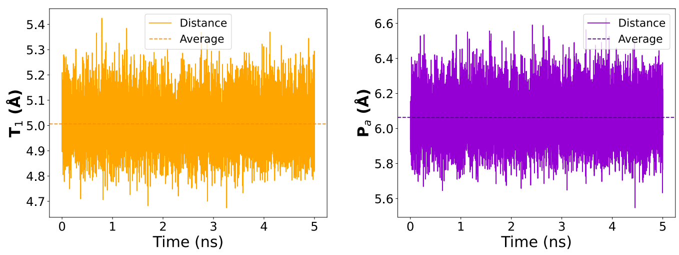


**Supplementary Figure 39: Characteristic distances between C8-DNTT-C8 molecules in a herringbone pattern.** (a) Herringbone pattern distances $P_a$, $P_b$, $T_1$, and $T_2$ are highlighted by the purple, blue, orange, and green arrows. (b), (c), (e), and (f) Figs correspond to the $T_1$, $P_b$, $T_2$, and $P_a$ distance fluctuations during the last equilibrated 5 ns of the MD simulation. The Table reported in panel (d) is related to the comparison between the distances found in the experimental crystalline structure and the average distances calculated from our simulations.

The last row reports the deviation of the simulated characteristic distances with respect to the experiments.

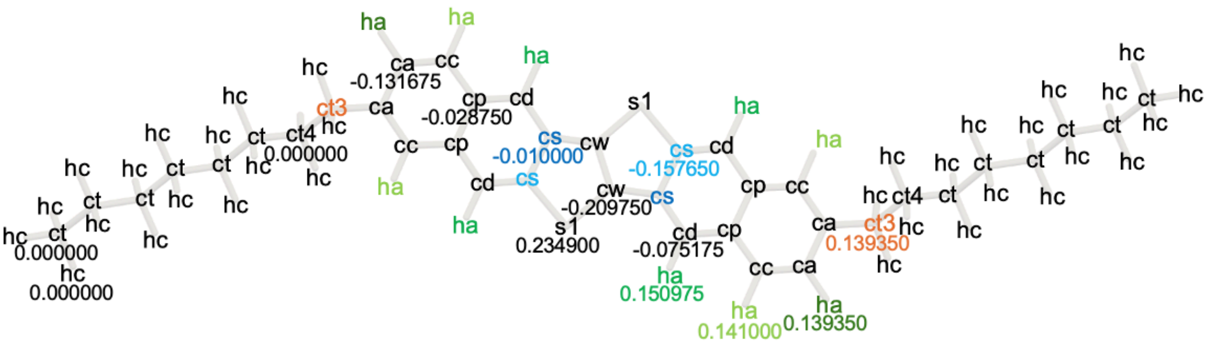


**Supplementary Figure 40: GAFF force field atom types defined for C8-DNTT-C8 and their charge computed by the localized bond-charge corrected CM1A (1.14*CM1A-LBCC) charge model [27].**

**Supplementary Table 15: Bond parameters of C8-DNTT-C8**

| | $k_r$ (kcal/mol/Å$^2$) | $r_{eq}$ (Å) |
|---|---|---|
| cw cw | 504.0 | 1.371 |
| cs cs | 418.3 | 1.429 |
| cs cw | 418.3 | 1.429 |
| s1 cw | 277.9 | 1.739 |
| s1 cs | 277.9 | 1.739 |
| cs cd | 504.0 | 1.371 |
| cd cp | 449.9 | 1.406 |
| cd ha | 344.3 | 1.087 |
| cp cp | 418.3 | 1.429 |
| cp cc | 411.7 | 1.434 |
| cc ca | 478.4 | 1.387 |
| ca ca | 478.4 | 1.387 |
| cc ha | 344.3 | 1.087 |
| ca ha | 344.3 | 1.087 |
| ca ct3 | 317.0 | 1.510 |
| ct3 ct4 | 268.0 | 1.529 |
| ct3 hc | 340.0 | 1.090 |
| ct4 ct | 268.0 | 1.529 |
| ct4 hc | 340.0 | 1.090 |
| ct ct | 268.0 | 1.529 |
| hc ct | 340.0 | 1.090 |

**Supplementary Table 16: Angle parameters of C8-DNTT-C8**

| | $k_\theta$ (kcal/mol/rad$^2$) | $\theta_{eq}$ (degrees) |
|---|---|---|
| cw s1 cs | 41.420 | 90.360 |
| s1 cw cs | 77.520 | 129.370 |
| s1 cs cs | 80.870 | 112.750 |
| s1 cw cw | 80.870 | 112.750 |
| s1 cs cd | 79.470 | 122.860 |
| cs cs cw | 67.660 | 111.040 |
| cw cs cs | 68.230 | 113.510 |
| cs cd ha | 49.070 | 120.760 |

| | | |
|---|---|---|
| cs cd cp | 67.180 | 119.970 |
| cd cp cp | 67.180 | 119.970 |
| cd cp cc | 64.880 | 120.660 |
| cp cp cc | 67.180 | 119.970 |
| cp cc ha | 48.460 | 120.010 |
| cp cc ca | 64.880 | 120.660 |
| cc ca ca | 65.990 | 120.100 |
| cc ca ha | 48.460 | 120.010 |
| ca ca ha | 48.460 | 120.010 |
| ca cc ha | 48.460 | 120.010 |
| cd cs cs | 65.990 | 120.100 |
| cd cs cw | 77.520 | 129.370 |
| cp cd ha | 48.460 | 120.010 |
| cc ca ct3 | 62.610 | 118.060 |
| ca ct3 ct4 | 63.250 | 112.090 |
| ca ct3 hc | 46.960 | 110.150 |
| ca ca ct3 | 62.610 | 118.060 |
| ct3 ct4 ct | 63.210 | 110.630 |
| ct3 ct4 hc | 46.370 | 110.050 |
| ct4 ct3 hc | 46.370 | 110.050 |
| ct4 ct ct | 63.210 | 110.630 |
| ct4 ct hc | 46.370 | 110.050 |
| ct ct4 hc | 46.370 | 110.050 |
| ct ct ct | 63.210 | 110.630 |
| ct ct hc | 46.370 | 110.050 |
| hc ct3 hc | 39.430 | 108.350 |
| hc ct4 hc | 39.430 | 108.350 |
| hc ct hc | 39.430 | 108.350 |

**Supplementary Table 17: Dihedral parameters of C8-DNTT-C8**

| | $k_\phi$ (kcal/mol) | multiplicity | $\phi$ (degrees) |
|---|---|---|---|
| cs cs cw cw | 2.170 | 2 | 180.0 |
| cs s1 cw cw | 2.170 | 2 | 180.0 |
| cs s1 cw cs | 2.170 | 2 | 180.0 |
| cp cd cs cw | 2.170 | 2 | 180.0 |
| ha cd cs cw | 7.250 | 2 | 180.0 |
| cd cs cs cw | 7.250 | 2 | 180.0 |
| cd cs cw cw | 2.170 | 2 | 180.0 |
| s1 cs cs cw | 7.250 | 2 | 180.0 |
| cp cd cs cs | 7.000 | 2 | 180.0 |
| ha cd cs cs | 7.250 | 2 | 180.0 |
| cs cs s1 cw | 7.250 | 2 | 180.0 |
| cp cp cd cs | 7.000 | 2 | 180.0 |
| cc cp cd cs | 7.000 | 2 | 180.0 |
| cs cw cw cs | 2.170 | 2 | 180.0 |
| cs cw cw s1 | 2.170 | 2 | 180.0 |
| cd cs cs cd | 7.250 | 2 | 180.0 |
| cd cs cs s1 | 7.250 | 2 | 180.0 |
| ca cc cp cd | 7.000 | 2 | 180.0 |
| ha cc cp cd | 7.250 | 2 | 180.0 |
| ha cd cp cc | 7.250 | 2 | 180.0 |

| | | | |
|---|---|---|---|
| cd cs s1 cw | 7.250 | 2 | 180.0 |
| cc cp cp cc | 7.250 | 2 | 180.0 |
| cc cp cp cd | 7.000 | 2 | 180.0 |
| cd cp cp cd | 7.250 | 2 | 180.0 |
| ca ca cc cp | 7.250 | 2 | 180.0 |
| ha ca cc cp | 7.250 | 2 | 180.0 |
| ca cc cp cp | 7.000 | 2 | 180.0 |
| ha cc cp cp | 7.250 | 2 | 180.0 |
| ha cd cp cp | 7.250 | 2 | 180.0 |
| cc ca ca cc | 7.250 | 2 | 180.0 |
| ha ca ca cc | 7.250 | 2 | 180.0 |
| ha cc ca ca | 7.250 | 2 | 180.0 |
| s1 cs cd cp | 7.000 | 2 | 180.0 |
| s1 cs cd ha | 7.250 | 2 | 180.0 |
| s1 cw cw s1 | 7.250 | 2 | 180.0 |
| s1 cw cs cd | 2.170 | 2 | 180.0 |
| s1 cw cs cs | 2.170 | 2 | 180.0 |
| ha ca ca ha | 7.250 | 2 | 180.0 |
| ha ca cc ha | 7.250 | 2 | 180.0 |
| X ct3 ca X | 0.000 | 2 | 0.0 |
| X ct3 ct4 X | 0.1556 | 3 | 0.0 |
| ct3 ca cc cp | 7.250 | 2 | 180.0 |
| ct3 ca ca cc | 7.250 | 2 | 180.0 |
| ha ca ca ct3 | 7.250 | 2 | 180.0 |
| ha cc ca ct3 | 7.250 | 2 | 180.0 |
| X ct4 ct X | 0.1556 | 3 | 0.0 |
| X ct ct X | 0.1556 | 3 | 0.0 |

**Supplementary Table 18: Non-bonded parameters of C8-DNTT-C8**

| | ε (kcal/mol) | r (Å) | $\varepsilon_{1\text{-}4}$ (kcal/mol) | $r_{1\text{-}4}$ (Å) |
|---|---|---|---|---|
| ha | 0.030000 | 1.45900 | 0.015000 | 1.45900 |
| ca | 0.070000 | 1.90800 | 0.035000 | 1.90800 |
| cc | 0.070000 | 1.90800 | 0.035000 | 1.90800 |
| cd | 0.070000 | 1.90800 | 0.035000 | 1.90800 |
| cs | 0.070000 | 1.90800 | 0.035000 | 1.90800 |
| cw | 0.080000 | 1.90800 | 0.040000 | 1.90800 |
| cp | 0.080000 | 1.90800 | 0.040000 | 1.90800 |
| s1 | 0.355000 | 2.00000 | 0.177500 | 2.00000 |
| ct3 | 0.066000 | 1.90800 | 0.033000 | 1.90800 |
| ct4 | 0.066000 | 1.90800 | 0.033000 | 1.90800 |
| ct | 0.066000 | 1.90800 | 0.033000 | 1.90800 |
| hc | 0.030000 | 1.48700 | 0.015000 | 1.48700 |

## Structural analysis

As already mentioned in the main paper, using the example of deformation along the x-axis (see Supplementary Figure 41), when the DNTT is deformed along one direction, only the characteristic distances of the crystalline structure with a component along the direction of the deformation will shrink during contraction or expand during elongation. Indeed, we can see in Supplementary Figure 42 that the distances $P_b$, $T_1$, and $T_2$ change during the y-oriented deformation, while $P_a$ and $P_c$ parameters remain unchanged. On the other side, during the deformation along z, the only distance affected is $P_c$, see Supplementary Figure 43. The same dependency from the parameters to the direction of strain application (*i.e.* along x, y and z, in Supplementary Figures 44, 45, and 46, respectively) was observed for the C8-DNTT-C8 derivative.

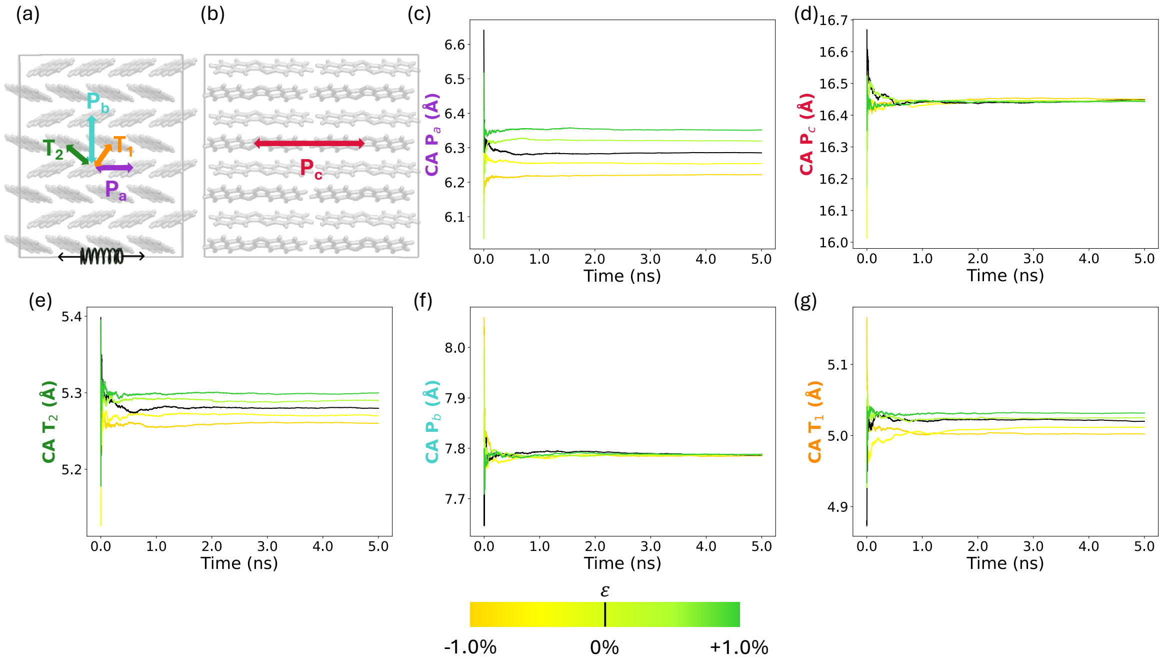


**Supplementary Figure 41: Evolution of the DNTT crystal characteristic distances after the application of uniaxial strain along the x-axis.** (a) shows the supercell in the xy-plane: the characteristic structural distances, namely the $P_a$, $P_b$, $T_1$, and $T_2$, are represented by the purple, blue, orange, and green arrows, respectively. (b) shows the supercell in the yz-plane highlighting the distance $P_c$ (red arrow). (c), (d), (e), (f), and (g) show the cumulative average (CA) of distances $P_a$, $P_c$, $T_2$, $P_b$, and $T_1$, respectively during a 5 ns MD simulation for different strains along the x-axis.

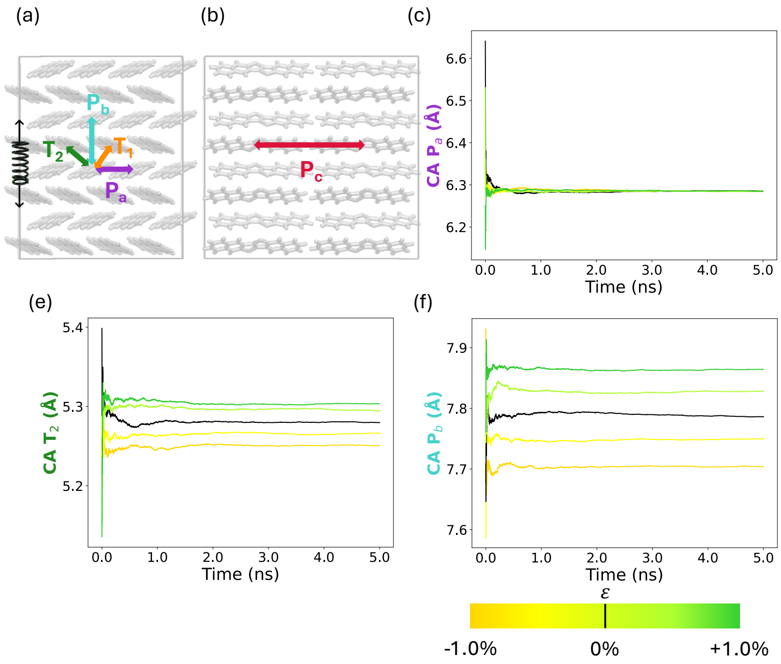


**Supplementary Figure 42: Evolution of the DNTT crystal characteristic distances after the application of uniaxial strain along the y-axis.** (a) shows the supercell in the xy-plane: the characteristic structural distances, namely the $P_a$, $P_b$, $T_1$, and $T_2$, are represented by the purple, blue, orange, and green arrows, respectively. (b) shows the supercell in the yz-plane highlighting the distance $P_c$ (red arrow). (c), (d), (e), (f), and (g) show the cumulative average of distances $P_c$, $P_a$, $T_2$, $P_b$, and $T_1$, respectively during a 5 ns MD simulation for different strains along the y-axis.

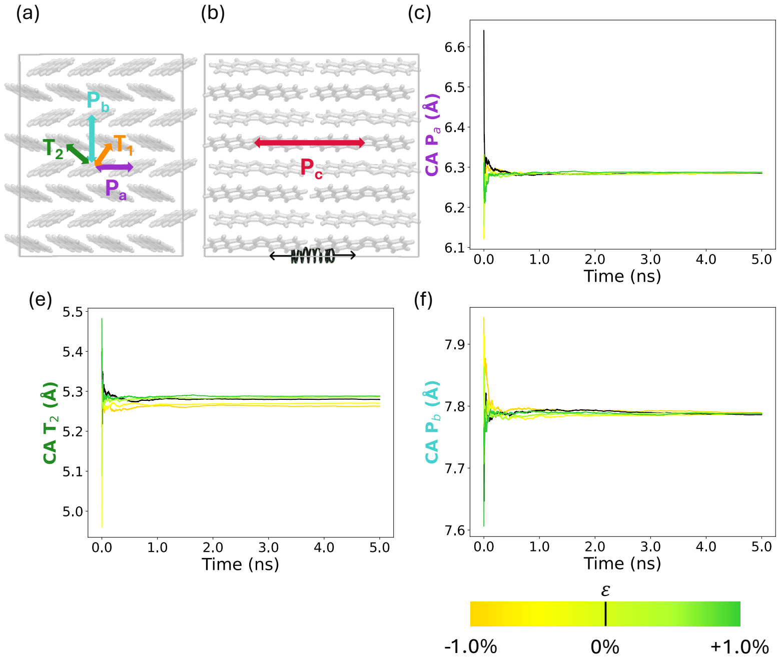


**Supplementary Figure 43: Evolution of the DNTT crystal characteristic distances after the application of uniaxial strain along the z-axis.** (a) shows the supercell in the xy-plane: the characteristic structural distances, namely the $P_a$, $P_b$, $T_1$, and $T_2$, are represented by the purple,

blue, orange, and green arrows, respectively. (b) shows the supercell in the yz-plane highlighting the distance $P_c$ (red arrow). (c), (d), (e), (f), and (g) show the cumulative average of distances $P_c$, $P_a$, $T_2$, $P_b$, and $T_1$, respectively during a 5 ns MD simulation for different strains along the z-axis.

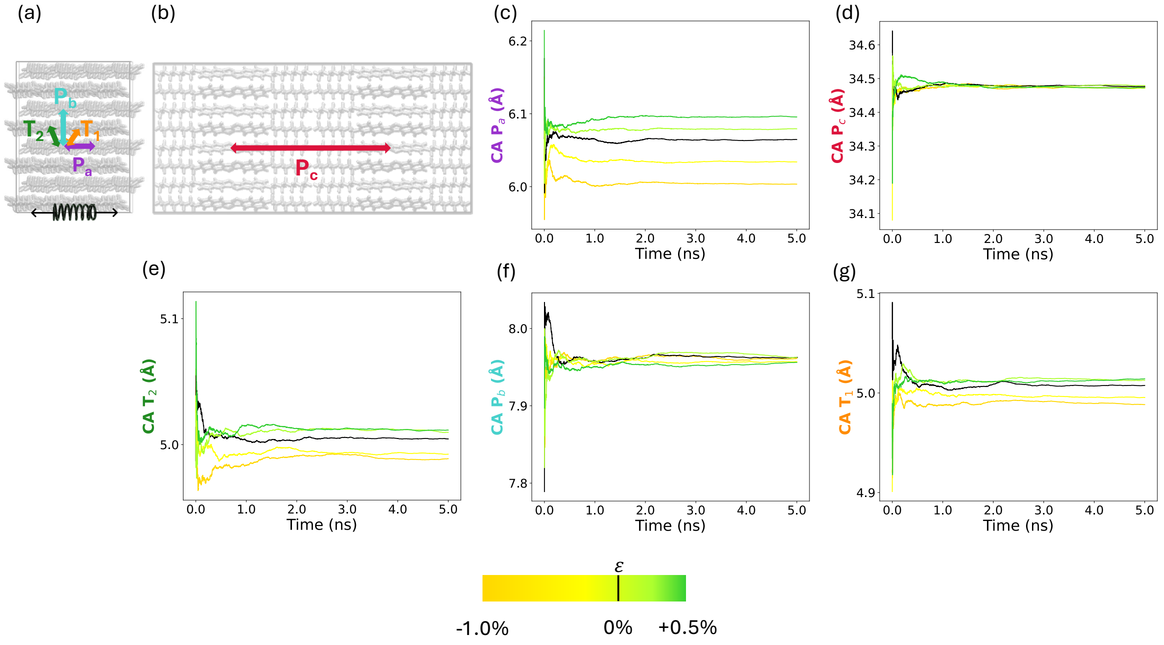


**Supplementary Figure 44: Evolution of the C8-DNTT-C8 crystal characteristic distances after the application of uniaxial strain along the x-axis.** (a) shows the supercell in the xy-plane: the characteristic structural distances, namely the $P_a$, $P_b$, $T_1$, and $T_2$, are represented by the purple, blue, orange, and green arrows, respectively. (b) shows the supercell in the YZ-plane highlighting the distance $P_c$ (red arrow). (c), (d), (e), (f), and (g) show the cumulative average of distances $P_c$, $P_a$, $T_2$, $P_b$, and $T_1$, respectively during a 5 ns MD simulation for different strains along the x-axis.

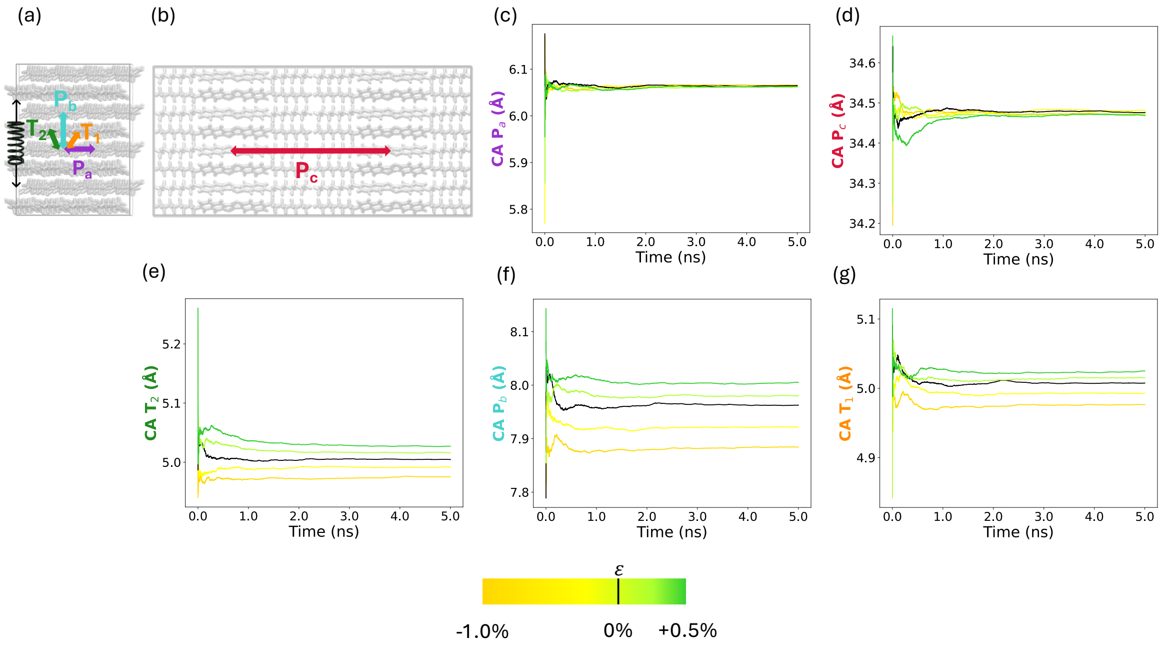

**Supplementary Figure 45: Evolution of the C8-DNTT-C8 crystal characteristic distances after the application of uniaxial strain along the y-axis.** (a) shows the supercell in the xy-plane: the characteristic structural distances, namely the $P_a$, $P_b$, $T_1$, and $T_2$, are represented by the purple, blue, orange, and green arrows, respectively. (b) shows the supercell in the YZ-plane highlighting the distance $P_c$ (red arrow). (c), (d), (e), (f), and (g) show the cumulative average of distances $P_c$, $P_a$, $T_2$, $P_b$, and $T_1$, respectively during a 5 ns MD simulation for different strains along the y-axis.

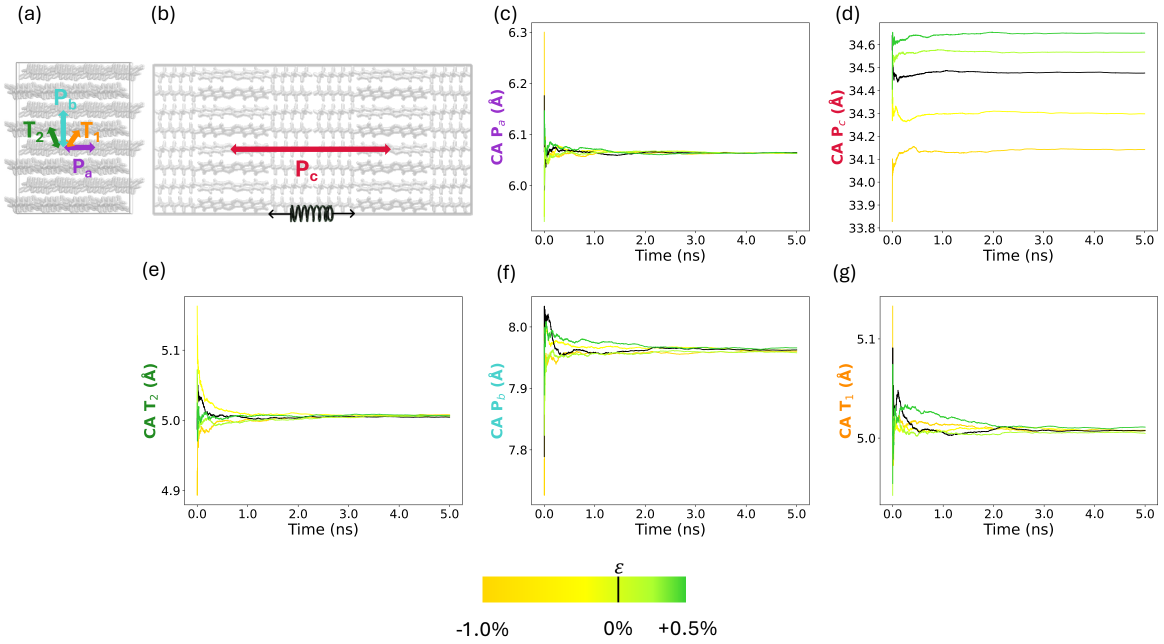

**Supplementary Figure 46: Evolution of the C8-DNTT-C8 crystal characteristic distances after the application of uniaxial strain along the z-axis.** (a) shows the supercell in the xy-plane: the characteristic structural distances, namely the $P_a$, $P_b$, $T_1$, and $T_2$, are represented by the purple,

blue, orange, and green arrows, respectively. (b) shows the supercell in the YZ-plane highlighting the distance $P_c$ (red arrow). (c), (d), (e), (f), and (g) show the cumulative average of distances $P_c$, $P_a$, $T_2$, $P_b$, and $T_1$, respectively during a 5 ns MD simulation for different strains along the z-axis.

We also decided to investigate the evolution of the angles corresponding to the different typical distances of the herringbone structure. While we calculated the distances between the centers of mass, in the case of the angles, we looked at the angles between the planes formed by the central atoms of the DNTT and C8-DNTT-C8 molecules. In this case, all the angles are affected by the deformations along the x, y, and z axes, see Supplementary Figures 47 to 52. This shows a real restructuration of the system during uniaxial strains.

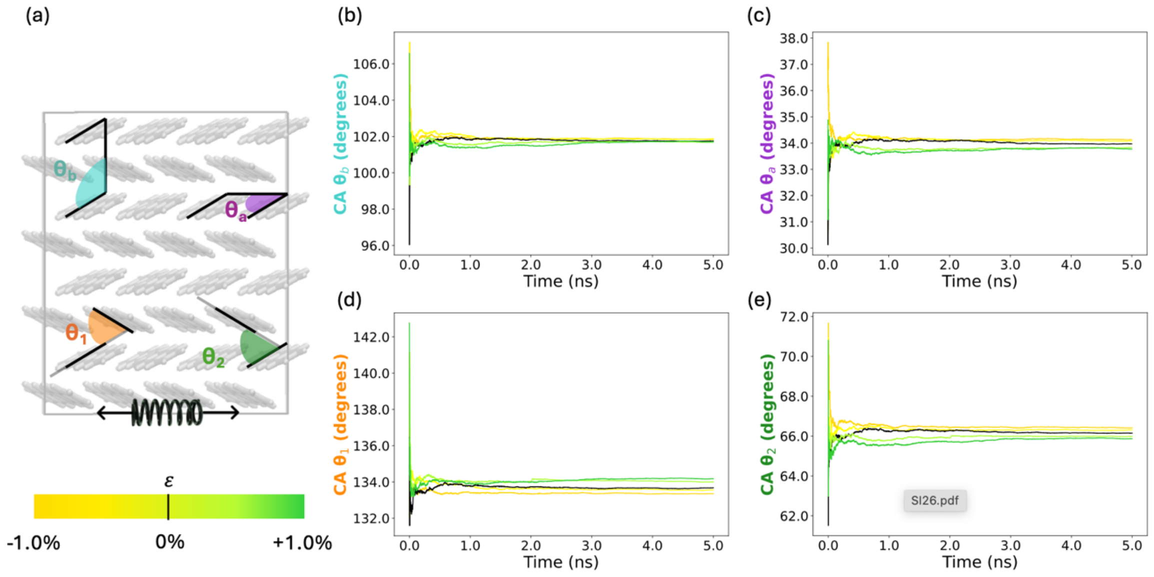


**Supplementary Figure 47: Evolution of the crystal DNTT characteristic angles when the cell is stretched along the x-axis.** (a) shows the supercell in the xy-plane: the characteristic herringbone angles, namely the $\theta_b$, $\theta_a$, $\theta_1$, and $\theta_2$, are represented by the blue, purple, orange, and green angles, respectively. (b), (c), (d), and (e) show the cumulative average of angles $\theta_b$, $\theta_a$, $\theta_1$, and $\theta_2$ during a 5 ns MD simulation for different strains along the x-axis.

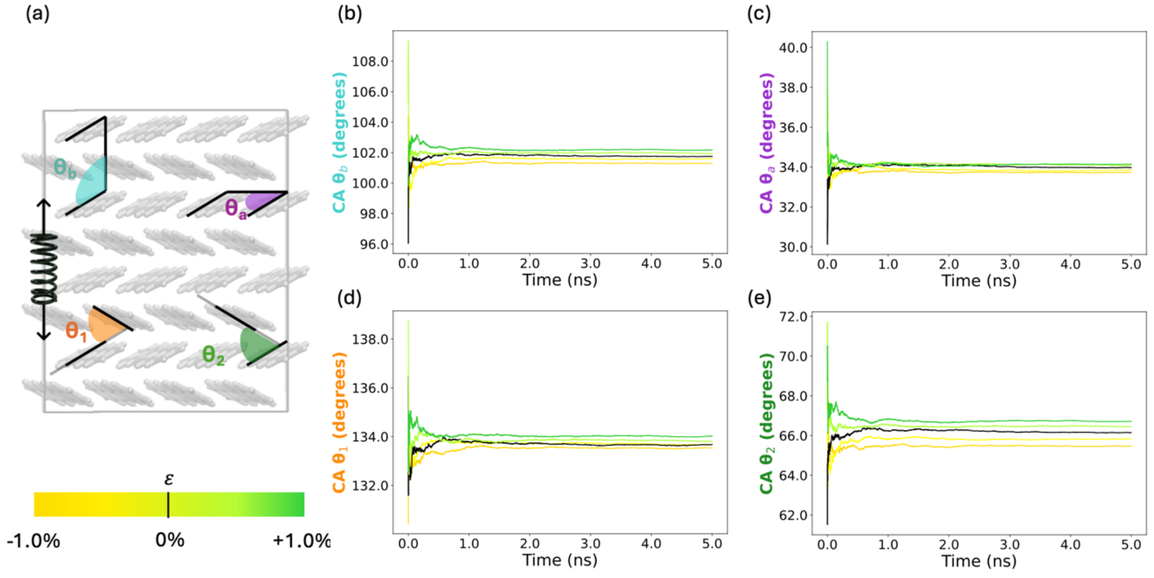

**Supplementary Figure 48: Evolution of the crystal DNTT characteristic angles when the cell is stretched along the y-axis.** (a) shows the supercell in the xy-plane: the characteristic herringbone angles, namely the $\theta_b$, $\theta_a$, $\theta_1$, and $\theta_2$, are represented by the blue, purple, orange, and green angles, respectively. (b), (c), (d), and (e) show the cumulative average angles $\theta_b$, $\theta_a$, $\theta_1$, and $\theta_2$ during a 5 ns MD simulation for different strains along the y-axis.

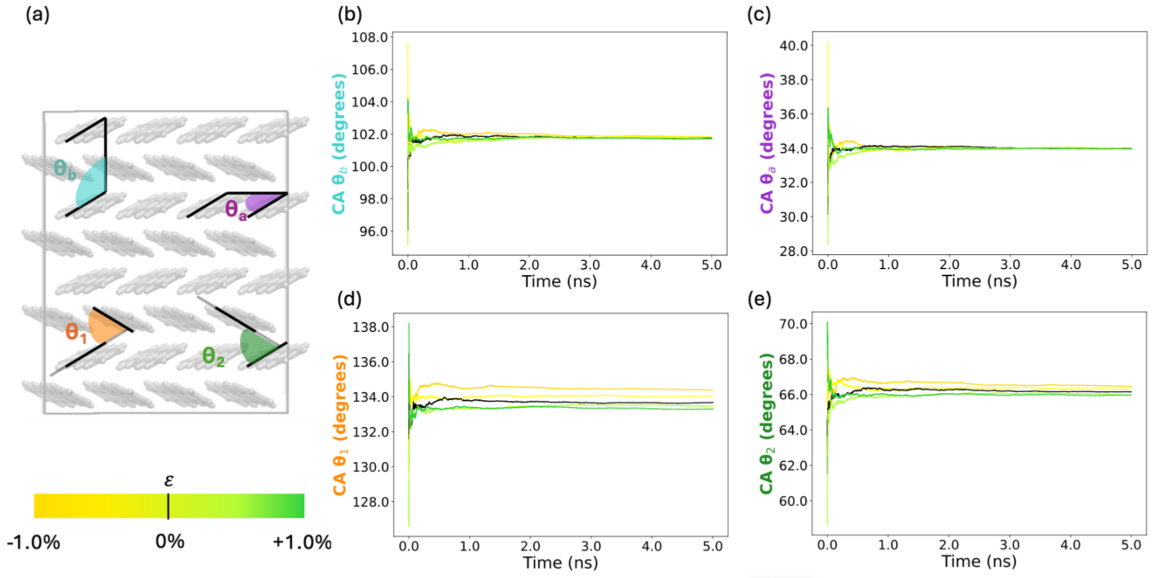


**Supplementary Figure 49: Evolution of the crystal DNTT characteristic angles when the cell is stretched along the z-axis.** (a) shows the supercell in the xy-plane: the characteristic herringbone angles, namely the $\theta_b$, $\theta_a$, $\theta_1$, and $\theta_2$, are represented by the blue, purple, orange, and green angles, respectively. (b), (c), (d), and (e) show the cumulative average of angles $\theta_b$, $\theta_a$, $\theta_1$, and $\theta_2$ during a 5 ns MD simulation for different strains along the z-axis.

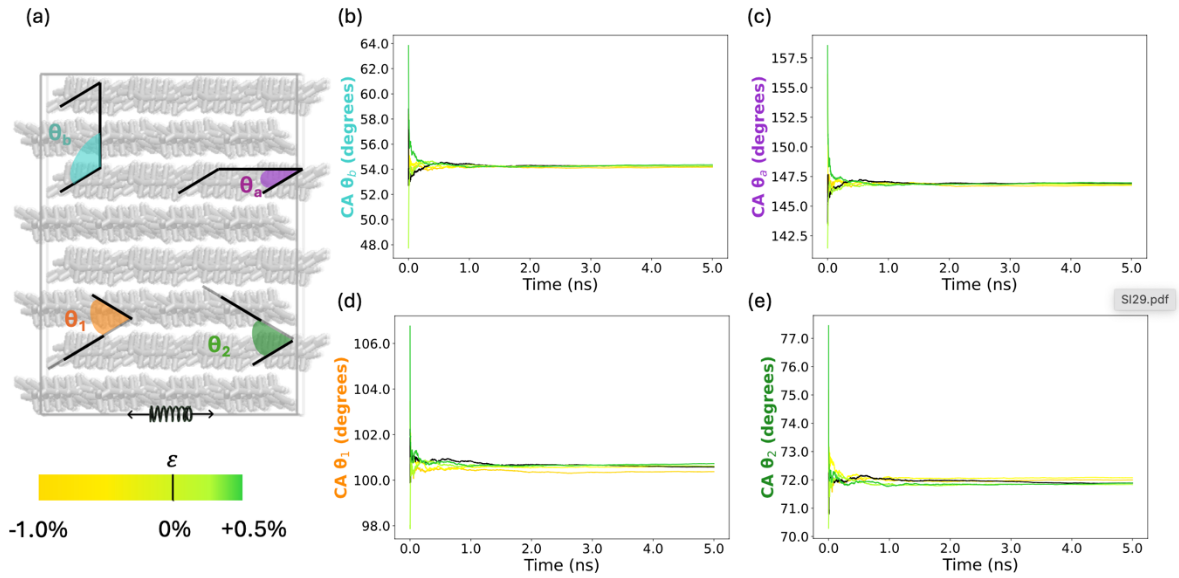


**Supplementary Figure 50: Evolution of the crystal C8-DNTT-C8 characteristic angles when the cell is stretched along the x-axis.** (a) shows the supercell in the xy-plane: the characteristic herringbone angles, namely the $\theta_b$, $\theta_a$, $\theta_1$, and $\theta_2$, are represented by the blue, purple, orange,

and green angles, respectively. (b), (c), (d), and (e) show the cumulative average of angles $\theta_b$, $\theta_a$, $\theta_1$, and $\theta_2$ during a 5 ns MD simulation for different strains along the x-axis.

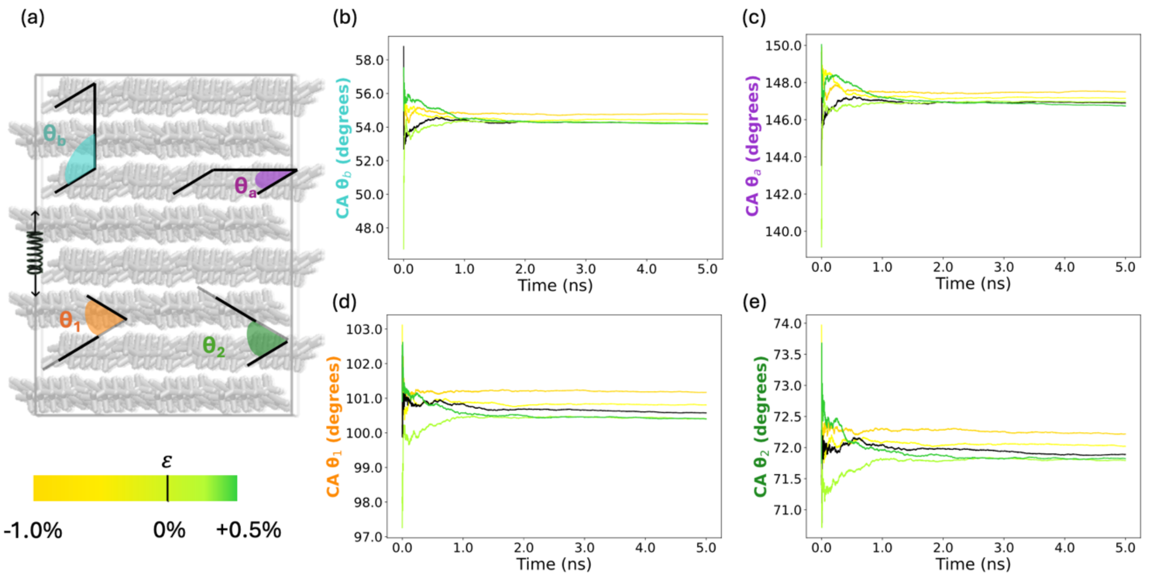


**Supplementary Figure 51: Evolution of the crystal C8-DNTT-C8 characteristic angles when the cell is straightened along the y-axis.** (a) shows the supercell in the xy-plane: the characteristic herringbone angles, namely the $\theta_b$, $\theta_a$, $\theta_1$, and $\theta_2$, are represented by the blue, purple, orange, and green angles, respectively. (b), (c), (d), and (e) show the cumulative average of angles $\theta_b$, $\theta_a$, $\theta_1$, and $\theta_2$ during a 5 ns MD simulation for different strains along the y-axis.

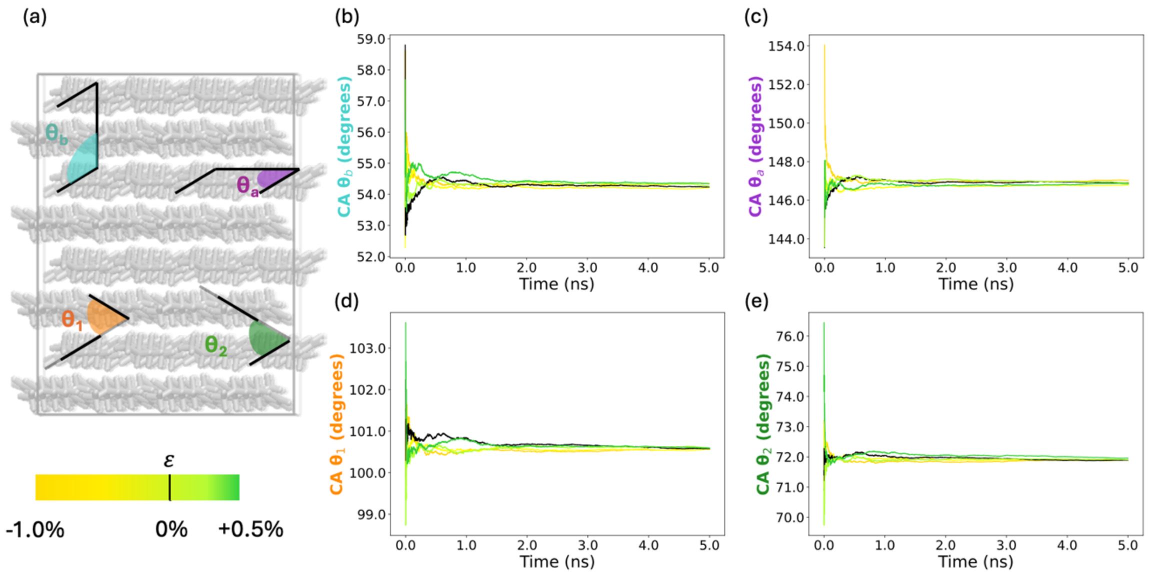


**Supplementary Figure 52: Evolution of the crystal C8-DNTT-C8 characteristic angles when the cell is straightened along the z-axis.** (a) shows the supercell in the xy-plane: the characteristic herringbone angles, namely the $\theta_b$, $\theta_a$, $\theta_1$, and $\theta_2$, are represented by the blue, purple, orange, and green angles, respectively. (b), (c), (d), and (e) show the cumulative average of angles $\theta_b$, $\theta_a$, $\theta_1$, and $\theta_2$ during a 5 ns MD simulation for different strains along the z-axis.

**SI Section 8**
**Transfer (linear regime) and output characteristics of field-effect transistors based on DNTT, C8-DNTT-C8, RR-C8*-DNTT-C8* and SS-C8*-DNTT-C8*.**

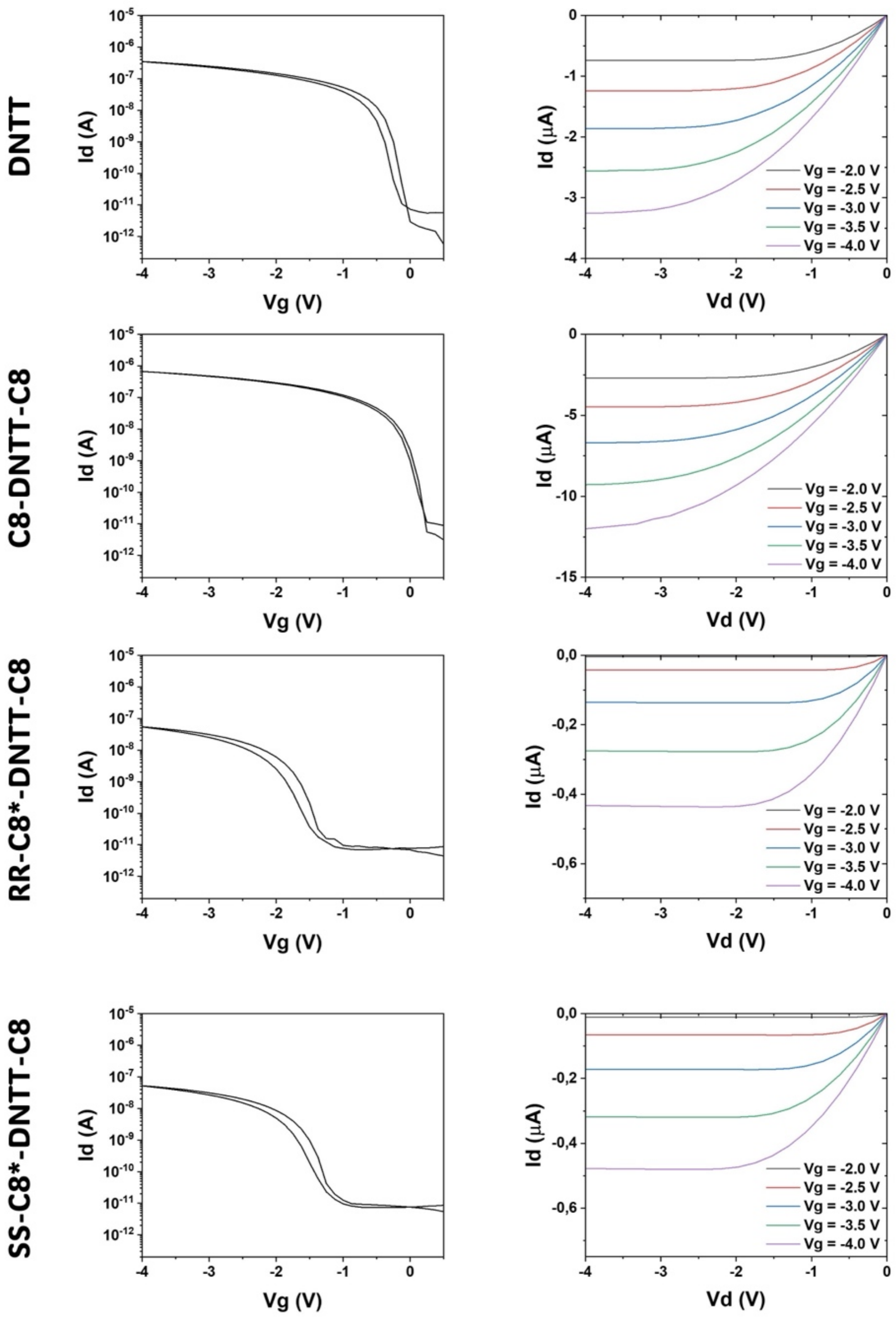


**Supplementary Figure 53** Representative transfer (linear regime $V_d$ = -0.1 V) and output characteristics of bottom gate bottom contact (BGBC) field-effect transistors based on DNTT, C8-DNTT-C8, RR-C8*-DNTT-C8* and SS-C8*-DNTT-C8*.  W/L = 480/215 μm.

**Supplementary Table 19:** Electrical performance of BGBC field-effect transistors in the linear regime

| Compound | μ ($cm^2V^{-1}s^{-1}$) | $V_{th}$ (V) | SS (mV/dec) | $I_{ON}/I_{OFF}$ |
|---|---|---|---|---|
| **DNTT** | 2.3 ± 0.1 | -0.7 ± 0.1 | 100 ± 8 | $1 \times 10^6$ |
| **C8-DNTT-C8** | 4.8 ± 0.3 | -0.4 ± 0.1 | 100 ± 3 | $3 \times 10^5$ |
| **RR-C8*-DNTT-C8*** | 0.5 ± 0.1 | -1.5 ± 0.1 | 130 ± 10 | $2 \times 10^4$ |
| **SS-C8*-DNTT-C8*** | 0.6 ± 0.1 | -1.4 ± 0.1 | 129 ± 2 | $2 \times 10^4$ |

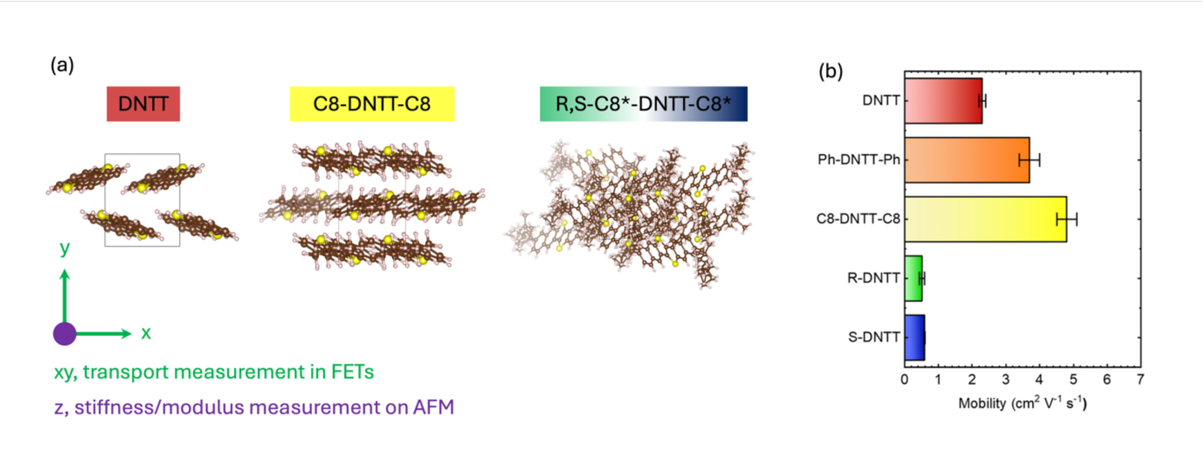


**Supplementary Figure 54 In-plane charge transport in DNTT and its derivatives.** (a) Configuration of molecular packing in the in-plane transport direction. (b) Measured charge carrier mobilities of DNTT and its derivatives.

## Bibliography


[1] M. E. Dokukin and I. Sokolov, “Nanoscale compositional mapping of cells, tissues, and polymers with ringing mode of atomic force microscopy,” *Scientific Reports,* vol. 7, no. 1, p. 11828, 2017.

[2] J. E. Northrup, “Two-dimensional deformation potential model of mobility in small molecule organic semiconductors,” *Appl. Phys. Lett.,* vol. 99, p. 062111 , 2011.

[3] S. Lyonnais, M. Hénaut, A. Neyret, P. Merida, C. Cazevieille, N. Gros, C. Chable-Bessia and D. Muriaux, “Atomic force microscopy analysis of native infectious and inactivated SARS-CoV-2 virions,” *Scientific Reports,* vol. 11, no. 1, p. 11885, 2021.

[4] I. Dobryden, V. V. Korolkov, V. Lemaur, M. Waldrip, H.-I. Un, D. Simatos, L. J. Spalek, O. D. Jurchescu, Y. Olivier, P. M. Claesson and D. Venkateshvaran, “Dynamic self-stabilization in the electronic and nanomechanical properties of an organic polymer semiconductor,” *Nature Communications,* vol. 13, p. 3076, 2022.

[5] S. Kim, Y. Lee, M. Lee, S. An and S.-J. Cho, “ Quantitative Visualization of the Nanomechanical Young’s Modulus of Soft Materials by Atomic Force Microscopy,” *Nanomaterials,* vol. 11, p. 1593, 2021.

[6] L. Wang, H. Wang and X. G. Xu, “Principle and applications of peak force infrared microscopy,” *Chemical Society Reviews,* vol. 51, no. 13, pp. 268-5286, 2022.

[7] W. C. Oliver and G. M. Pharr, “An improved technique for determining hardness and elastic modulus using load and displacement sensing indentation experiments,” *Journal of Materials Research,* vol. 7, no. 6, pp. 1564-1583, 1992.

[8] C. A. Schuh, “Nanoindentation studies of materials,” *Materials Today,* vol. 9, no. 5, pp. 32-40, 2006.

[9] F. Cellini, Y. Gao and E. Riedo, “Å-Indentation for non-destructive elastic moduli measurements of supported ultra-hard ultra-thin films and nanostructures,” *Scientific Reports,* vol. 9, no. 1, p. 4075, 2019.

[10] R. Martinez and L. R. Xu, “ Comparison of the Young's moduli of polymers measured from nanoindentation and bending experiments,” *MRS Communications,* vol. 4, no. 3, pp. 89-93, 2014.

[11] M.-C. Jung, M. R. Leyden, G. O. Nikiforov, M. V. Lee, H.-K. Lee, T. J. Shin, K. Takimiya and Y. Qi, “Flat-lying semiconductor–insulator interfacial layer in DNTT thin films,” *ACS Applied Materials & Interfaces,* vol. 7, no. 3, pp. 1833-1840, 2015.

[12] D. J. Müller and A. Engel, “Atomic force microscopy and spectroscopy of native membrane proteins,” *Nature Protocols,* vol. 2, no. 9, pp. 2191-2197, 2007.

[13] E. Dague, V. Pons, A. Roland, J.-M. Azaïs, S. Arcucci, V. Lachaize, S. Velmont, E. Trevisiol, D. N’Guyen, J.-M. Sénard and C. Galés, “Atomic force microscopy-single-molecule force spectroscopy unveils GPCR cell surface architecture,” *Communications Biology,* vol. 5, no. 1, p. 221, 2022.

[14] H. P. Lang and C. Gerber, “Up close & personal with atoms & molecules,” *Materials Today,* vol. 12, no. 7-8, pp. 18-25, 2009.

[15] J. F. Nye, Physical properties of crystals: their representation by tensors and matrices, Oxford: Clarendon Press, 1957.

[16] V. Blum, R. Gehrke, F. Hanke, P. Havu, V. Havu, X. Ren, K. Reuter and M. Scheffler, "Ab initio molecular simulations with numeric atom-centered orbitals," *Comput. Phys. Commun.,* vol. 180, p. 2175, 2009.

[17] V. Havu, V. Blum, P. Havu and M. Scheffler, "Efficient O(N) integration for all-electron electronic structure calculation using numeric basis functions," *J. Comp. Phys.,* vol. 228, p. 8367, 2009.

[18] V. W. Yu, F. Corsetti, A. Garcia, W. P. Huhn, M. Jacquelin, W. Jia, B. Lange, L. Lin, J. Lu, W. Mi, A. Seifitokaldani, Vazquez-Mayagoitia, C. Yang, H. Yang and V. Blum, "ELSI: A unified software interface for Kohn–Sham electronic structure solvers," *Comput. Phys. Comm.,* vol. 222, p. 267, 2018.

[19] F. Knuth, C. Carbogno, V. Atalla, V. Blum and M. Scheffler, "All-electron formalism for total energy strain derivatives and stress tensor components for numeric atom-centered orbitals," *Computer Physics Communications,* vol. 190, p. 33, 2015.

[20] A. Becke, "On the large-gradient behavior of the density functional exchange energy," *J. Chem. Phys.,* p. 7184, 1986.

[21] J. P. Perdew, K. Burke and M. Ernzerhof, "Generalized Gradient Approximation Made Simple," *Phys. Rev. Lett.,* vol. 77, p. 3865, 1996.

[22] A. Becke and E. R. Johnson, "Exchange-hole dipole moment and the dispersion interaction revisited," *The Journal of Chemical Physics,* vol. 127, p. 154108, 2007.

[23] A. J. A. Price, A. Otero-de-la Roza and E. R. Johnson, "XDM-corrected hybrid DFT with numerical atomic orbitals predicts molecular crystal lattice energies with unprecedented accuracy," *Chem. Sci.,* vol. 14, p. 1252, 2023.

[24] R. Yu, J. Zhu and H. Ye, "Calculations of single-crystal elastic constants made simple," *Comput. Phys. Commun.,* vol. 181, p. 671, 2010.

[25] J. C. Philips, D. J. Hardy, J. D. C. Maia, J. E. Stone, J. V. Ribeiro, R. C. Bernardi, R. Buch, G. Fiorin, J. Hénin, W. Jiang, R. McGreevy, M. C. R. Melo, B. K. Radak, R. D. Skeel and A. Singharoy, "Scalable molecular dynamics on CPU and GPU architechtures with NAMD," *J. Chem. Phys.,* vol. 153, p. 044130, 2020.

[26] J. Wang, R. M. Wolf, J. W. Caldwell, P. A. Kollman and D. A. Case, "Development and testing of a general amber force field," *J. Comput. Chem.,* vol. 25, pp. 1157-1174, 2004.

[27] E. Slezneva, A. Vercouter, G. Schweicher, V. Lemaur, K. Broch, A. Antidormi, K. Takimiya, V. Coropceanu, J.-L. Brédas, C. Melis, J. Cornil and H. Sirringhaus, "Strong SUppression of Thermal Conductivity in the Presence of Long terminal Alkyl Chains in Low-Disorder Molecular Semiconductors," *Adv. Mater.,* vol. 33, p. 2008708, 2021.

[28] T. Yamamoto and K. Takimiya, "Facile Synthesis of Highly π-Extended Heteroarenes, Dinaphtho[2,3-b:2‘,3‘-f]chalcogenopheno[3,2-b]chalcogenophenes, and Their Application to Field-Effect Transistors," *J. Am. Chem. Soc.,* vol. 129, pp. 2224-2225, 2007.

[29] G. Schweicher, G. D'Avino, M. T. Ruggiero, D. J. Harkin, K. Broch, D. Venkateshvaran, G. Liu, A. Richard, C. Ruzié, J. Armstrong, A. R. Kennedy, K. Shankland, K. Takimiya and Y. Geerts, "Chasing the "Killer" Phonon Mode for the Rational Design of Low-

Disorder, High-Mobility Molecular Semiconductors," *Adv. Mater,* vol. 31, p. 1902407, 2019.

[30] U. Essmann, L. Perera, M. L. Berkowitz, T. Darden, H. Lee and L. G. Pedersen, "A smooth particle mesh Ewald method," *J. Chem. Phys.,* vol. 103, p. 8577-8593, 1995.

[31] M. Matta, M. J. Pereira, S. M. Gali, D. Thuau, Y. Olivier, A. Briseno, I. Dufour, C. Ayela, G. Wantz et L. Muccioli, Unusual Electromechanical Response in Rubrene Single Crystals, *Mater. Horiz.,* vol. 5, p. 41-50, 2018.

[32] J.K.D. Verma and B.D. Nag, "On the Elastic Moduli of a Crystal and Voigt and Reuss Relations", *J. Phys. Soc. Japan,* vol. 20, p. 635, 1965.

[33] J.-I. Park, J. W. Chung, J.-Y. Kim, J. Lee, J. Y. Jung, B. Koo, B.-L. Lee, S. W. Lee, Y. W. Jin et S. Y. Lee, Dibenzothiopheno[6,5-b:6',5'-f]thieno[3,2-b]thiophene (DBTTT): High-Performance Small-Molecule Organic Semiconductor for Field-Effect Transistors, *J. Am. Chem. Soc.,* vol. 137, p. 12175-12178, 2015.

[34] Advanced Chemistry Development (ACD/Labs) Software V11.02, 1994-2024. [Online]. Available: https://www.acdlabs.com.
